# Novel Therapeutics Identification for Fibrosis in Renal Allograft Using Integrative Informatics Approach

**DOI:** 10.1038/srep39487

**Published:** 2017-01-04

**Authors:** Li Li, Ilana Greene, Benjamin Readhead, Madhav C. Menon, Brian A. Kidd, Andrew V. Uzilov, Chengguo Wei, Nimrod Philippe, Bernd Schroppel, John Cijiang He, Rong Chen, Joel T. Dudley, Barbara Murphy

**Affiliations:** 1 Department of Genetics and Genomic Sciences, Icahn School of Medicine at Mount Sinai, 770 exington Ave., New York, NY 10065, USA; 2 Division of Nephrology, Department of Medicine, Icahn School of Medicine at Mount Sinai, One Gustave L. Levy Place, New York, NY 10029, USA; 3 Department of Genetics and Genomic Sciences, Icahn School of Medicine at Mount Sinai, 1255 5th Avenue, New York, NY 10029, USA; 4 Section of Nephrology, University of Ulm, Albert-Einstein-Allee 23, Ulm, 89081 Germany; 5 Department of Health Policy and Research, Icahn School of Medicine at Mount Sinai, One Gustave L Levy Place, New York, NY 10029, USA.; 6 Institute for Next Generation Healthcare, Icahn School of Medicine at Mount Sinai.

## Abstract

Chronic allograft damage, defined by interstitial fibrosis and tubular atrophy (IF/TA), is a leading cause of allograft failure. Few effective therapeutic options are available to prevent the progression of IF/TA. We applied a meta-analysis approach on IF/TA molecular datasets in Gene Expression Omnibus to identify a robust 85-gene signature, which was used for computational drug repurposing analysis. Among the top ranked compounds predicted to be therapeutic for IF/TA were azathioprine, a drug to prevent acute rejection in renal transplantation, and kaempferol and esculetin, two drugs not previously described to have efficacy for IF/TA. We experimentally validated the anti-fibrosis effects of kaempferol and esculetin using renal tubular cells *in vitro* and *in vivo* in a mouse Unilateral Ureteric Obstruction (UUO) model. Kaempferol significantly attenuated TGF-β1-mediated profibrotic pathways *in vitro* and *in vivo*, while esculetin significantly inhibited Wnt/β-catenin pathway *in vitro and in vivo*. Histology confirmed significantly abrogated fibrosis by kaempferol and esculetin *in vivo*. We developed an integrative computational framework to identify kaempferol and esculetin as putatively novel therapies for IF/TA and provided experimental evidence for their therapeutic activities *in vitro* and *in vivo* using preclinical models. The findings suggest that both drugs might serve as therapeutic options for IF/TA.

Chronic allograft damage, represented by interstitial fibrosis and tubular atrophy in allografts (IF/TA) is the leading cause of allograft failure despite the improvement in immunosuppression therapies and short-term graft survival^1^,^2^,^3^. Natural history studies in allografts have shown that the rate of progression of IF/TA remains relatively constant, with limited improvements in slowing progressive deterioration of kidney function and extending graft survival^4^,^5^. Also, early chronic histological damage or kidney fibrosis was significantly associated with graft loss as an independent risk factor, even in the absence of progressive disease (e.g., antibody-meditated rejection or glomerulonephritis)^6^. Moreover, the global burden of early chronic histological damage within the first year after renal transplantation greatly affects the outcome of the allografts^6^. The extent of disease progression varies on presentation amongst renal transplant recipients, and evidence of a specific underlying etiology is usually lacking^7^,^8^. Experimental studies in animal models attempting to treat IF/TA demonstrate effects in mitigating renal fibrosis. However, these experimental interventions prove to be difficult to implement in clinical practice, and currently available treatments cannot effectively prevent or revert the progression of IF/TA and improve renal graft function^9^,^10^,^11^,^12^. Chronic Allograft Dysfunction Index (CADI) is a broader histologic assessment for fibrosis in protocol biopsies that consists of 6 histologic parameters: interstitial inflammation in non-fibrotic areas (ii), interstitial fibrosis (IF), mesangial matrix (mm), vascular intimal proliferation (cv), tubular atrophy (TA), and glomerulosclerosis (gs)^13^,^14^.

The pathophysiology of IF/TA is incompletely understood. Notably, IF/TA is also encountered in native kidneys with progressive diseases irrespective of etiology, and associates with functional decline and the development end-stage renal failure. Further, IF/TA in both allograft and native kidneys appear to share common risk loci strongly suggesting common pathogenetic mechanisms involved in renal fibrosis^15^,^16^. Multiple etiological factors may culminate in a common phenotype of IF/TA through several stereotypic phases^17^,^18^. Early injury to the allograft occurs as a result of transplantation-related injury (from ischemia and reperfusion) and consequent non-allospecific inflammation likely leading on to allospecific immune activation^19^. This phase is followed by an enhancement of fibrogenic responses, culminating in the final phase of matrix accumulation and evident tissue fibrosis and the pathologic picture of IF/TA^20^. In both native and allograft-kidneys, fibrogenic processes are driven by well-described pathways involving transforming growth factor (TGF)-β, Wnt/β-catenin, NFκB, Notch, and other growth factors^10^,^21^,^22^,^23^. TGF-β has been implicated in the pathogenesis of IF/TA^24^ through its contribution to the development of EMT^25^,^26^,^27^,^28^, and via downstream *Smad* signaling^11^,^29^. Therefore, TGF-β-signaling pathway may serve as a potential target for treating IF/TA in native and allograft kidneys^20^,^30^,^31^. TGF-β is known to induce the expression of Wnt/β-catenin superfamily members and cross-talk with the Wnt/β-catenin, and has been described as a promising new target for the treatment of fibrosis^10^,^22^,^16^,^32^ Therefore, the TGF-β and Wnt/β-catenin pathways are attractive therapeutic targets for the slowing or preventing the progression of chronic allograft damage^11^,^21^.

The growing body of data from molecular profiling studies of IF/TA offers new opportunities for drug and biomarker discovery. Past studies of fibrosis in allograft show that kidney allograft biopsy and peripheral blood exhibit identifiable coordinated expression changes at the mRNA level^33^,^34^,^35^,^36^,^37^. However, many of the existing studies are single-center studies with often limited sample sizes, and comparison between studies raises challenges due to the variability of microarray platform, patient sample, and experiment protocols^38^. In this study, we developed a computational framework for utilizing publicly available data sets obtained NCBI Gene Expression Omnibus (GEO). Specifically, we obtained gene expression (i.e., transcriptome) datasets deposited into GEO from 6 different transplant centers. Our approach entails a systematic integration of the of genomic data using a meta-analysis technique followed by a drug repositioning approach for identifying novel therapeutic agents and targets for IF/TA.

We hypothesized that a molecular signature derived from a meta-analysis of multi-center independent gene expression data sets would yield a more robust molecular descriptor of IF/TA, which would improve therapeutic discovery by a computational drug repurposing approach. We identified a robust common transcriptional response in IF/TA consisting of 85 significantly expressed genes in IF/TA vs. non-IF/TA identified across multiple studies. Then, building on our previous systematic drug repurposing work^39^, we compared these 85 IF/TA gene signatures to the reference drug expression signatures from the Connectivity Map (cMAP)^40^ to infer possible therapeutic indications based on their expression. We identified two compounds, kaempferol and esculetin, that our method predicts will perturb the expression levels of the 85-gene signature specific to IF/TA in a therapeutic direction. We validated the therapeutic effects of these compounds using *in vitro* and *in vivo* models. We found that the two drugs independently exhibit anti-fibrotic effects in an experimental model of renal fibrosis. Further, we identified potential cellular pathways and transcriptional mechanisms that facilitate the therapeutic effects of these compounds in models of IF/TA. Our findings present evidence that our computational strategy can uncover new uses for drugs for IF/TA as well as highlight novel targets and pathways for development of IF/TA therapies.

## Results

### Meta-analysis of transplant datasets reveals known molecular mechanisms of IF/TA

We obtained raw data for six high-throughput gene expression studies from both peripheral blood and biopsy samples of renal transplant patients, with biopsy-proven IF/TA defined by CADI or IF/TA scores (S. Table 1). We used the high CADI or moderated and severe IF/TA phenotypes reported from the corresponding studies to increase the signal and identify clinically significant IF/TA. The resulting data set contained a total of 275 samples from 3 different microarray platforms on 2 tissues after manually curating datasets. All probe sets were re-annotated to the most recent NCBI Entrez gene identifier (Gene ID) by AILUN (ref. 41, http://ailun.ucsf.edu) then mapped by Gene ID, yielding 23,762 unique common genes across six platforms. We then performed quantile-quantile normalization for all 6 datasets to compensate systematic technical variation for samples^42^.

**Figure 1:**
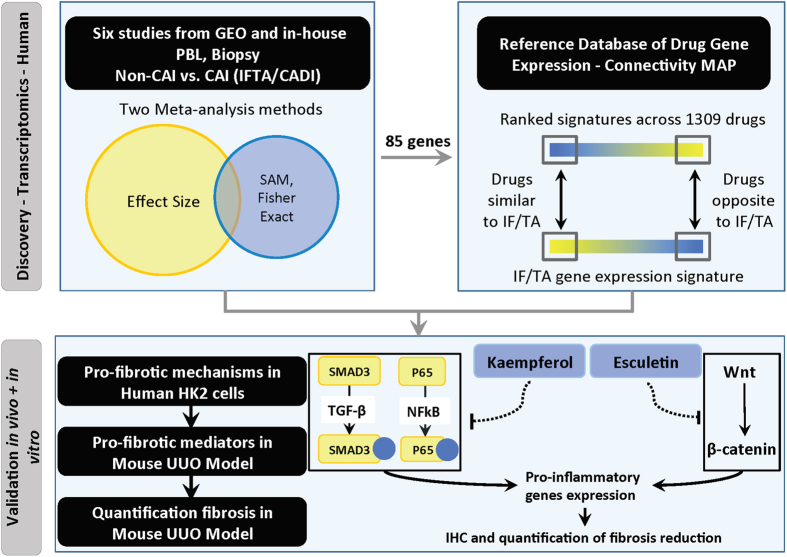
Work Flow of Identifying the Drug Targets through Integrative Informatics Approach.

To derive a consensus molecular signature of differentially expressed genes in IF/TA across multiple data sets, we applied two meta-analysis methods to the normalized data (See methods, Fig. 1). First, we calculated a meta-effect size for each gene, weighted by the variance in the effect size within each of the six studies. We identified 996 genes that were measured in at least 4 out of 6 datasets and were both up or down regulated in IF/TA with false discovery rate (FDR) ≤ 5%. Secondly, we calculated enrichment of meta-SAM q values (≤10%) by the Fisher Exact test to identify the genes significantly expressed in all 6 datasets. We identified 510 genes that were significantly expressed in IF/TA with at least 3 out of 6 datasets with P ≤ 5%. Based on two meta-analysis methods, we have identified a total of 85 overlapped genes significantly specific to IF/TA. Up and down-regulated genes show consistence in gene expression for the IF/TA and non-IF/TA samples across the 6 different datasets by heatmap (S. Figure 1A–F). Meta-effect size, meta-SAM q values, effect size for each dataset, and SAM q values for each dataset were shown in S. Table 2A.

**Figure 2:**
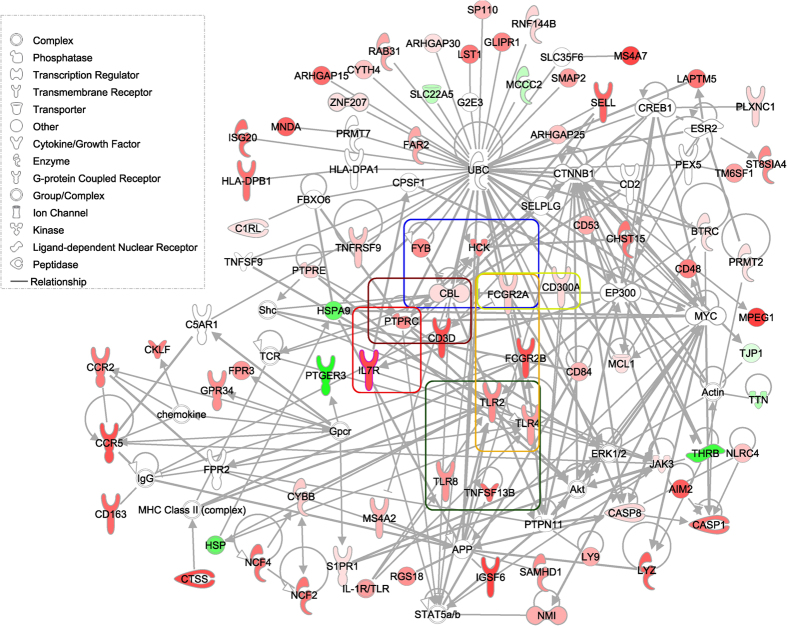
IPA regulatory network using 75 of the 85 genes specific to IF/TA. We used 85 significantly expressed genes by two meta-analysis methods as input to IPA to create a gene-gene interaction network. We chose the direct relationship option in the IPA to create the interaction networks, resulting in 75 genes. The gradient of the red and green represent the positive and negative meta-effect size respectively. We highlighted the IF/TA biological relevant molecules which were significantly associated with communication between innate and adaptive immune cells in green rectangle, Fcγ receptor-mediated phagocytosis in macrophages and monocytes in blue rectangle, B cell development in red rectangle, T cell receptor signaling in dark red rectangle, dendritic cell maturation in orange rectangle, and natural killer cell signaling in yellow rectangle.

**Figure 3:**
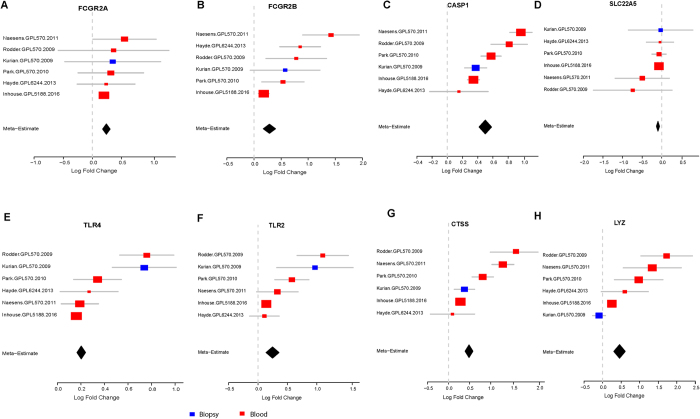
8 genes associated with kidney failure and tubular toxicity. Forest plots are presented as in **3**(**A**–**H**) in 6 independent studies consisting of 275 kidney transplant samples. Meta effect size was in black diamond, and biopsy and peripheral blood samples were in red and blue respectively.

We performed pathway and network analysis for the 85 genes using QIAGEN’s Ingenuity Pathway Analysis (IPA, QIAGEN Redwood City, http://www.qiagen.com/ingenuity) and discovered that 75 genes (88%, 75/85) were involved in immune cell trafficking, inflammatory response, cell death and survival, and cellular growth and proliferation biological functions (Fig. 2). The genes include major histocompatibility complex class II, interleukins, chemokine receptors, toll-like receptors, and T cell receptors, which are well known to be involved in kidney injury^43^,^44^,^45^,^46^,^47^,^48^. Similarly, canonical pathways for the 85 genes were related to the immune response, including dendritic (DC) cells, T cells, B cells, natural killer (NK) cells, Toll-like receptor, and NFkB signaling pathway (S. Table 2B). Three molecules, *UBC, MYC*, and *CTNNB1*, were connected with the most nodes, and associated with Wnt/β-catenin signaling pathway. *BTRC*, which is up-regulated in IF/TA, binds *UBC* and increases transcriptional activity of human *β-catenin (CTNNB1*) protein (Fig. 2). Eight genes related to kidney toxicity, including kidney failure (*CASP1, FCGR2A, FCGR2B, TLR2, TLR4*, P = 0.003), damage of renal tubule (*TLR2, TLR4*, P = 0.01), proximal tubular toxicity (*CTSS, LYZ, SLC22A5*, P = 0.007) and their expression across 6 datasets were not confounded by tissue types (Fig. 3A–H). The 85 genes are significantly enriched (10× median intensity across all tissues; http://www.BioGPS.org)^49^,^50^ in different blood cells, namely CD14+ Monocytes (25, P_adj_ = 2.25E-27), CD19+ B cells (12, P_adj_ = 3.41E-11), DC Cells (12, P_adj_ = 3.55E-10), CD4+ T cells (10, P_adj_ = 3.21E-8), CD8+ T cells (10, P_adj_ = 3.86E-8), and CD56+ NK cells (8, P_adj_ = 3.23E-5).

### Computational prediction and assessment of novel drugs for IF/TA

We used the 85 IF/TA-specific genes to prioritize a library of 1,309 drug induced transcriptional profiles^40^ according to their predicted ability to perturb genes in the IF/TA signature, thus deriving a therapeutic ranking score for each compound. Score significance was estimated by generating an empirical score distribution for connections between IF/TA and 1,000 permuted drug signatures (see Methods). Compounds predicted to induce a state that is negatively correlated with IF/TA, (negative therapeutic ranking score, P ≤ 0.05) were shortlisted for further analysis. Among the strongest therapeutic predictions for IF/TA was azathioprine (the 133^rd^ rank, score = −0.53, P = 0.04), which is known to prevent acute rejection in renal transplantation^51^ and treat pulmonary fibrosis^52^ (S. Figure 2). We performed a literature review of the most highly ranked drugs, and excluded those with profiles that would be impediments to their clinical application in IF/TA, such as having severe neurological and psychiatric side effects (S. Table 3). We identified two drugs, kaempferol and esculetin, that had acceptable toxicity profiles and that were known to induce biological perturbations relevant to fibrosis. Kaempferol (3,5,7-trihydroxy-2-(4-hydroxyphenyl)-4H-1-benzyopyran-4-one, the 85^th^ rank, score = −0.57, P = 0.018), is a naturally occurring flavanol-type flavonoid and found in natural food such as fruits and vegetables^53^. It suppresses TGF-β-triggered Epithelial to Mesenchymal Transition (EMT) a possible mechanism for the generation of myofibroblast that contribute to fibrosis, and myofibroblast formation with concomitant restoration of E-cadherin expression, expression reduction of N-cadherin and α-SMA and ECM component deposition^54^. Additionally it has been used as a target for therapeutic strategies in preventing airway fibrotic diseases^54^ and schistosoma egg-induced hepatic fibrosis^55^. The second compound, esculetin (6,7-dihydroxycoumarin, the 37^th^ rank, score = −0.52, P = 0.005), is a naturally occurring plant coumarin derivative and antioxidant^56^. It has been reported to decrease fibrosis and glomerulosclerosis in diabetic nephropathy by inhibiting the PPARy/TGF-β pathway and reversing expression of *Bmp6* and *Mmp13*^57^. We show the key genes (from the 85 IF/TA genes) that represent the leading edge of the connectivity scores with kaempferol and esculetin in S. Figure 3. A complete list of significant compounds driven by 85 IF/TA genes was shown in S. Table 3.

Further, our analysis found that kaempferol and esculetin were likely to exhibit positive therapeutic effects in IF/TA beyond anti-fibrosis activity. As the 85 genes were derived from transplantation datasets and, likely incorporated signatures of ongoing allo-responses, we investigated whether kaempferol and esculetin could have specific effects on immune cells. We applied a recent developed immune-cell specific drug repurposing approach^58^ to evaluate putative immune modulatory actions of kaempferol and esculetin. Using this methodology we can identify specific immune cell subsets a drug may potentially influence. By comparing differences in immune cell signatures with transcriptional changes from drug treatment, we examined which specific immune cell subsets would be influenced by kaempferol or esculetin, and whether these interactions were likely to activate or suppress cells involved in an immune response to transplantation. This analysis predicted that esculetin would inhibit both adaptive and innate immune cells including CD4 T cells (P_adj_ = 9.76E-15), CD8 T cells (P_adj_ = 3.68E-13), dendritic (DC) cells (P_adj_ = 0.00015), B cells (P_adj_ = 1.64E-4), and natural killer (NK) cells (P_adj_ = 0.0015). Similarly, we predicted that kaempferol inhibit DC cells (P_adj_ = 6.24E-6), NK cell (P_adj_ = 3.18E-5), and NKT cell (P_adj_ = 0.00035) activation. These finding are consistent with the cell types enriched in the 85 gene meta-analysis signature for IF/TA, and suggest that the two drugs have a potential role in the suppression of deleterious immune response in IF/TA^59^. Thus, kaempferol and esculetin were considered as potential candidates for further validation because of their high therapeutic ranking score, lack of reported toxicity in humans, and scientific literature supporting their therapeutic effects in experimental settings similar to IF/TA (S. Figure 2, S. Table 3).

### Kaempferol and esculetin attenuate pro-fibrotic pathways *in vitro*

**Figure 4:**
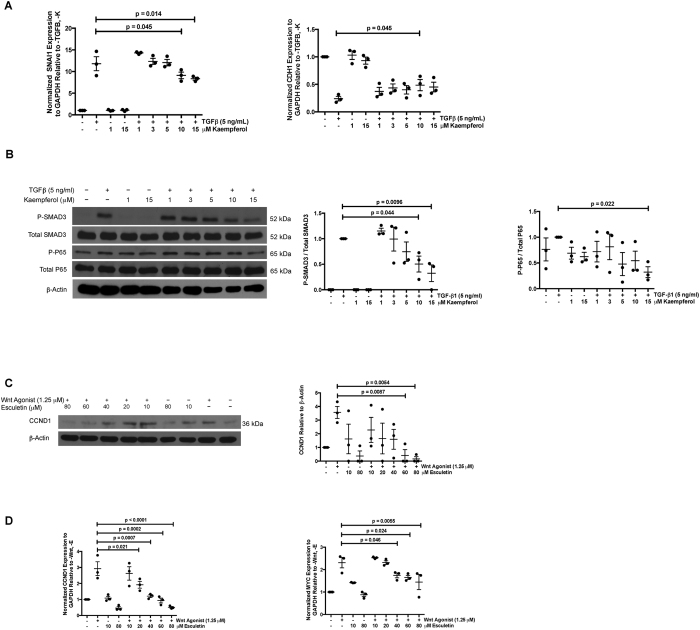
Validation of kaempferol and esculetin through TGF-β and Wnt/β-catenin pathways respectively in HK2 cells. (**A**) *SNAI1* and *CDH1* gene expression determined by RT-PCR (normalized to *GAPDH*) for cells treated with kaempferol (1–15 μM) for 12 hours followed by 12 hours of kaempferol in the presence of TGF-β1 (5 ng/ml). (**B**) Western blots and associated densitometries for *P-P65*, total *P65, P-SMAD3*, total *SMAD3* and *β-Actin* for HK2 cells treated with kaempferol (1–15 μM) for 16–24 hours followed by 20 minutes of kaempferol in the presence of TGF-β1 (5 ng/ml). Quantifications were shown next to western blots. (**C**) Western blot and associated densitometry for *CCND1* and *β-Actin* for HK2 cells treated with esculetin (10–80 μM) for 16 hours followed by 36 hours of esculetin in the presence of Wnt-agonist (1.25 μM). Quantifications were next to western blot. (**D**) *CCND1* and *MYC* gene expression determined by Q-PCR (normalized to *GAPDH*) for cells treated with esculetin (10–80 μM) for 16 hours followed by 8 hours of esculetin in the presence of Wnt-agonist (1.25 μM). N = 3 in each arm. Western blot experiments on (**B**) and (**C**) were run under the same experimental conditions. Data were represented in mean and standard error of the mean.

We performed an analysis to identify the cellular pathways that were significantly perturbed by kaempferol and esculetin using Human kidney 2 (HK2) cell lines. Given kaempferol’s inhibition of TGF-β signaling in airway fibrosis^54^, we investigated whether kaempferol could also decrease the effects of TGF-β signaling in renal tubular cells. HK2 cells were cultured in 0.5% nutrient medium with 1, 3, 5, 10, and 15 μM kaempferol for 24 hours. TGF- β (5 ng/ml) was added during the last 12-hours for gene expression studies. Recent data has shown *Snai1* to be a key downstream zinc finger transcriptional repressor essential to TGF-β mediated EMT in renal tubular cells^44^,^60^. We observed that treatment with 10 and 15 μM kaempferol significantly reduced the TGF-β1-mediated expression of *SNAI1* (N = 3 in each condition, P = 0.045 and 0.014), and correspondingly reversed the down regulation of *CDH1*, an epithelial cell marker known to be repressed by *SNAI1*^61^ (N = 3 in each condition, P = 0.045) (Fig. 4A). Next we examined the phosphorylation of *SMAD3*, a central signaling event in the canonical TGF-β pathway^62^,^63^. Human kidney 2 (HK2) cells were cultured with kaempferol as above, and then stimulated with TGF-β1 for 20 minutes. *SMAD3* phosphorylation was decreased when treated with 10 and 15 μM kaempferol (N = 3 in each condition, P = 0.0044 and 0.0096 respectively) (Fig. 4B). Additionally, since kaempferol has previously been found to inhibit NFkB, a known mediator of inflammatory signaling^64^, we incidentally observed that 15 μM kaempferol decreased the phosphorylation of NFkB P65 (P-P65) relative to total P65 in renal tubular cells (N = 3 in each condition, P = 0.022, Fig. 4B). These data suggest that kaempferol has inhibitory effects on TGF- β signaling in renal tubular cells.

Esculetin’s inhibition of canonical Wnt signaling through direct binding to β-catenin has been previously established in human colon cancer cells^65^, thus, we examined the effect of esculetin on HK2 cells stimulated with Wnt agonist. HK2 cells were cultured in 0.5% nutrient medium with 10, 20, 40, 60, and 80 μM esculetin for 16 hours, followed by 8 hours of Wnt agonist stimulation to examine transcriptional changes, and 36 hours of Wnt agonist stimulation to examine long-term alterations in protein abundance. After 36 hours of Wnt agonist stimulation, 60 and 80 μM esculetin decreased the protein levels of *CCND1* (N = 3 in each condition, P = 0.0087 and 0.0054 respectively, Fig. 4C), a well-known target of Wnt signaling^66^. After 8 hours of exposure to Wnt agonist, 20–80 μM esculetin decreased *CCND1* mRNA expression (N = 3 in each condition, P = 0.021, 0.0007, 0.0002, and <0.0001 respectively) and 40–80 μM esculetin decreased *MYC* transcripts (N = 3 in each condition, P = 0.046, 0.024, and 0.0055) (Fig. 4D). These data suggest inhibition of Wnt/β-Catenin signaling by esculetin in renal tubular cells.

Moreover, HK2 cell viability was not affected by either 1–15 μM kaempferol or 10–80 μM esculetin applied for 24 hours based on the trypan blue exclusion method (S. Figure 4A and B).

### Kaempferol and esculetin inhibit renal interstitial fibrosis *in vivo*

**Figure 5:**
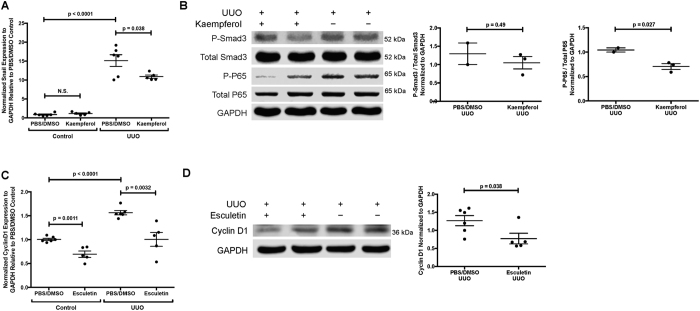
Kaempferol and esculetin inhibit pro-fibrotic mediators in 7 days UUO model. (**A**) *Snai1* gene expression of kaempferol treated mice (N = 5) compared with PBS/DMSO-treated mice (N = 6) in UUO model and controls. (**B**) Western blots in UUO model for *P-Smad3*, total *Smad3, P-p65*, total *p65*, and *GAPDH* between kaempferol treated mice (N = 3) and PBS/DMSO treated mice (N = 2). Quantifications were shown next to western blots. (**C**) *Cyclin D1* gene expression of esculetin treated mice (N = 5) compared with PBS/DMSO treated mice (N = 6) in UUO model and controls. (**D**) Western blots in UUO model for *Cyclin d1* and *GAPDH* between esculetin treated mice (N = 5) and PBS/DMSO treated mice (N = 6). Western blot experiments on (**B**) and (**D**) were run under the same experimental conditions. Quantifications were shown next to western blots. Data were represented in mean and standard error of the mean.

We then evaluated renal fibrogenesis *in vivo* with kaempferol and esculetin in a mouse unilateral ureteric obstruction model (UUO), a model of renal interstitial fibrosis. Kaempferol or esculetin treated BALB-C mice were subjected to UUO surgery as described earlier^16^. Tissues were harvested on day 7 post UUO and analyzed for gene-expression, histology and immunohistochemistry (IHC). PBS/DMSO-treated mice were used as controls. *Snai1* was significantly over expressed in UUO samples compared with controls (n = 6, P < 0.0001), and there was no significant difference for controls when treated with kaempferol. We demonstrated that kaempferol significantly decreased the expression the key transcriptional factor *Snai1* (N = 5, P = 0.038, Fig. 5A) compared with PBS/DMSO-treated mice (N = 6), suggesting inhibition of TGF-signaling, *in vivo*. Further, phosphorylation of *P65* was significantly inhibited by Western blot, in lysates from UUO-kidneys treated with kaempferol, compared to controls (Fig. 5B). Similarly, *cyclin d1* was significantly over expressed in UUO samples compared with controls (n = 5, P < 0.0001), and *cyclin d1* was significantly decreased in controls as well as UUO kidneys when treated with esculetin compared to vehicle (N = 6, P = 0.0011 for controls; and N = 5, P = 0.0032 for UUO, Fig. 5C), and a significant reduction in *cyclin d1* protein production (N = 5, P = 0.038) (Fig. 5D) compared with PBS/DMSO-treated mice (N = 5), thus lending further support to esculetin’s inhibition of Wnt signaling as a potential mechanism of its anti-fibrotic effect. There was no significant difference for P-Smad3, total Smad3, P-P65 and total P65 between PBS/DMSO vs. PBS/DMSO treated with kaempferol. Similarly, there was no significant difference for Cyclin D1 for esculetin, suggesting neither drug has effects on controls samples (S. Figure 5).

**Figure 6:**
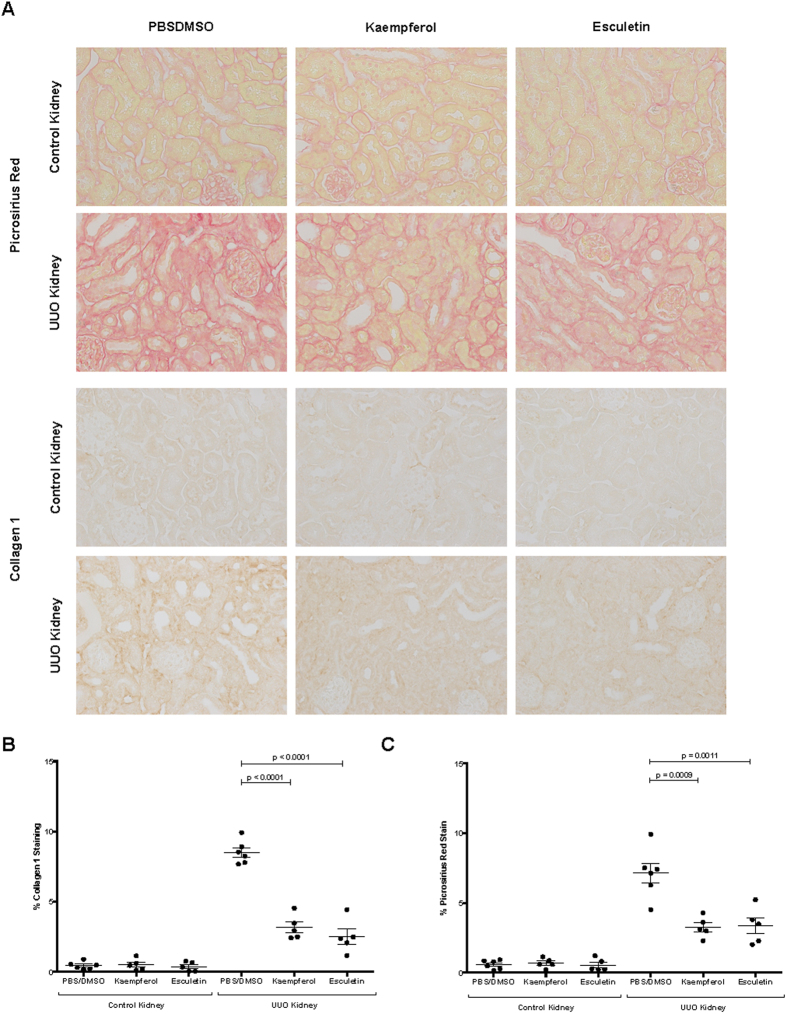
Kaempferol and esculetin reduce renal fibrosis in 7 days UUO model. (**A**) Representative images of picrosirius red stain (*collagen 1* and *collagen 3*) and *collagen 1* IHC of renal cortex. (**B**) Quantification of *collagen 1* IHC of renal cortex between UUO kidney and control kidney with PBS/DMSO (N = 6) or kaempferol treatment (N = 5). (**C**) Quantification of Picrosirius red (collagen 1 and collagen 3) stained renal cortex between UUO kidney and control kidney with PBS/DMSO (N = 6) or esculetin treatment (N = 5). Note: 10–15 random hpfs/animal, original magnification, x40. Data were represented in mean and standard error of the mean.

UUO kidneys from mice treated with kaempferol had a significant reduction in interstitial collagens and renal fibrosis by picrosirius red staining (P = 0.0009, Fig. 6A,C), and *Collagen-1* production compared with controls by IHC (P < 0.0001, Fig. 6A,B). Similarly, mice treated with esculetin had a significant reduction in interstitial fibrosis by picrosirius red staining (P = 0.0011, Fig. 6A,C) and anti-collagen 1 IHC staining (P < 0.0001, Fig. 6A,B). These data support our computational inferences, and confirm our *in vitro* findings demonstrating the beneficial *in vivo* effects of Kaempferol and Esculetin on renal fibrogenesis.

## Discussion

IF/TA is a progressive and irreversible process that represents the major barrier to long-term renal allograft survival. Despite increased efforts in therapeutic discovery, there remain unmet clinical needs for effective strategies to treat IF/TA. In this study, we aimed to identify novel therapeutic targets to potentially ameliorate IF/TA through an integrative computational framework. We hypothesized that a robust molecular signature from a meta-analysis of multi-center independent gene expression data sets integrated with a computational drug repurposing approach would suggest novel therapeutic targets for rational drug discovery and design.

Though several previous studies explored transcriptional patterns associated with IF/TA in renal transplantation, their small sample sizes limits their ability to capture the molecular heterogeneity of IF/TA^16^,^33^,^34^,^35^,^36^,^37^. Furthermore, experimental confounders, such as the variability of microarray platforms and experiment protocols in different centers, can confound the interpretation of transcriptional findings across studies. Our strategy employed a two-step meta-analysis of 275 biopsy and peripheral blood samples from publicly available microarray datasets. Additionally, we have performed similar statistical methods of SAM and effective size to identify significant gene list, then to identify the signified drugs from CMap for each of the 6 datasets. Interestingly, there are very few overlapped drugs across 4–5 datasets and none among all 6 datasets (S. Figure 6). Therefore, we proposed that meta-analysis of publicly available data from multiple centers can deliver a robust gene signature which implicitly accounts for the underlying molecular heterogeneity of IF/TA, the variability of the host response, differences in treatment protocols, and other clinical and sample confounding factors^67^. Our approach significantly increases sample size and ameliorates single study bias by integrating 6 independent datasets from 6 institutions. Our meta-analysis identified 85 genes that were associated with IF/TA. This meta-analysis signature was enriched for pathways and biological functions reflective of known IF/TA pathophysiology as well as potentially novel factors. We then sought to discover novel therapeutic relationships between drug compounds and IF/TA using a computational drug repurposing approach based on expression signatures of small molecule compounds^40^. We proposed that drugs that have a potentially therapeutic effect will be able to reverse the differential expression of the 85 IF/TA-specific gene set during drug exposure in a modeled cell line^39^. We selected two drugs for *in vitro* and *in vivo* validations, kaempferol and esculetin, two plant-derived compounds that were ranked at the top of the 1,309 compounds.

The therapeutic agents identified and experimentally validated in our study could form the basis of safer therapeutic strategies for IF/TA. Most patients still receive calcineurin inhibitor based immuosuppression protocols post kidney transplantation^68^. Studies have shown that the majority of patients receiving tacrolimus at 5 years post kidney transplantation develop CAN (≥Banff grade I) and with evidence of calcineurin inhibition (CNI) nephrotoxicity^19^. CNI nephrotoxicity plays an important role in late histologic injury and ongoing decline in renal function^19^. Therefore, avoiding adverse side effects of immunosuppressive therapy, especially nephrotoxicity, is a primary goal in designing an effective approaches for the prevention of IF/TA. Kaempferol and esculetin could potentially be administered as part of the long-term immunosuppression protocol, creating a balance between decreasing interstitial fibrosis without increasing immunological risk. Recent studies have identified plant-derived compounds that are effective as adjuvant therapies in the treatment of chronic conditions such as chronic HCV infection. One such example is ladanein (BJ486K), a flavonoid related to kaempferol^69^,^70^.

We demonstrate here that kaempferol and esculetin abrogated profibrotic molecular processes *in vitro* and *in vivo*, and decrease renal interstitial fibrosis. From our data, kaempferol’s anti-fibrotic effects are in part mediated by its effects on TGF-β signaling pathway. However, further studies are needed to study the exact site of interaction between Kaempferol and TGF-β signaling. While reduced phosphorylation of *smad3* suggests an effect on canonical-SMAD signaling, the incidental effects on P-P65 suggest a non-canonical pathway effect, whereas *Snai1* can be induced in response to TGF-β by both canonical and non-canonical downstream signals^71^. Indeed, *Snai1* is a widely implicated central transcription factor in EMT gene expression induced by TGF-β/smad3 signaling in fibrosis models^44^,^60^,^61^,^72^. While kaempferol decreased NFkB activation *in vivo*, we did not see a significant difference in inflammatory genes known to be mediated by NFkB activation (data not shown). Esculetin’s anti-fibrotic effects seemed to be mediated by its reduction of Wnt signaling due to its amelioration of *cyclin d1* gene expression and protein levels in the mouse UUO. *Cyclin d1* is a crucial regulator of Wnt-regulated organism development^66^. When Wnt binds its receptor, β-catenin is released to translocate from the cytoplasm to the nucleus and stimulates *cyclin d1* gene transcription^73^. This was further supported by esculetin’s reduction of Wnt-induced gene expression and protein levels *in vitro* using Wnt-agonist stimulated HK2 cells.

The 85 genes specific to IF/TA were significantly enriched for T cells, B cells, monocytes, NK, and DC cells and signaling pathways were involved in communication between innate immune (DC and NK cells) and adaptive immune cells (B and T cells), B cell development, DC maturation, B/T/NK cell receptor signaling, as well as several macrophages related immune response pathways (S. Table 2B). Previous studies indicate that macrophages mediate endothelial cell cytotoxicity leading to loss of renal microvasculature^74^, are predictive of IF/TA development in an early biopsy^75^, and that M2-type macrophages promote the development of interstitial fibrosis in IF/TA^76^,^77^. These studies support that macrophages play a significant role for IF/TA^9^. Our findings also support previous reports that the high CADI genes were enriched in proliferation of T and B cells, NK cell activation, and DC cell migration^34^, as well as two tolerance (TOL) biomarker lists (TOL vs. CAN) where NK and DC cell types are enriched in both liver and kidney transplantation^78^,^79^. Interestingly, kaempferol and esculetin were predicted to antagonize activations of T, B, NK, and DC cells. This concordance illustrates the complex interplay with potential therapeutic drugs between innate and adaptive immune responses in IF/TA after renal transplantation^80^. The subtle inflammatory infiltrates in IF/TA are important to the scarring process and for ultimate graft outcome^81^. Importantly, our transcriptomic data supports the growing theory that immune cell types such as T, B cells^82^, NK cells^83^,^84^, dendritic cells^85^,^86^,^87^ are involved in IF/TA and may be driving the immunological processes after transplantation, and possible therapeutic interventions such as kaempferol and esculetin could be applied to target these cell types. However, these predicted immune cell-specific inhibitory effects of kaempferol and esculetin cannot be confirmed or ruled out from our murine UUO model, a commonly used model for examining tubulointerstitial fibrosis^88^,^89^. While inflammation contributes to injury and fibrosis in this model, the abrogatory effects on fibrosis with kaempferol and esculetin observed here suggest a benefit that is independent of an allo-immune response and could indeed signal a wider potential role for these drugs in other chronic kidney diseases. Further studies using animal models of chronic allograft damage could specifically delineate the additional effects of these drugs on allo-immunity driven processes, although murine IF/TA models have suffered from lack of standardized technique and high complications^90^,^91^.

One potential limitation of our study is that it did not explore the entire space of therapeutic compounds. The current version of cMAP^40^ contains only 1,309 compounds on more than 7,000 expression profiles representing profiling from culture cell lines. The total coverage of compounds and limited drug perturbation gene expression data limited the drug search space. However, the compounds represented in cMAP have been used to identify numerous other novel therapeutic indications for existing drugs^39^,^92^. Another option is to extend this approach to other small molecule signature libraries, such as the Library of Integrated Network-based Cellular Signatures (LINCS) (http://www.ncbi.nlm.nih.gov/geo/query/acc.cgi?acc=GSE70138). This would allow evaluation of approximately 20,000 additional compounds, however these signatures are imputed from direct measurement of only 978 “Landmark” genes, rather than full microarray signatures used in the current study.

In conclusion, our current study is the first study of identifying novel therapeutic opportunities for IF/TA treatment through an integrative computational framework of transcriptome meta-analysis and drug repositioning approaches. We demonstrate that by expanding the sources of samples, tissue types, and various platforms from independent studies for IF/TA, we are able to identify a robust, biologically relevant, IF/TA-specific signature that can be useful for therapeutic repositioning. Kaempferol and esculetin have independently exhibited anti-fibrotic mechanisms in a model of renal fibrosis, and have abrogated cellular pathways and gene expressions involved in IF/TA and could be potentially used as novel adjuvant therapies that minimize side effects. Furthermore, our computational framework can be applied to address other unmet needs in transplantation, nephrology, and fibrosis.

## Materials and Methods

### Microarray datasets collection, preprocessing, and integration

We obtained 5 kidney transplant microarray datasets from GEO and 1 in-house microarray dataset (S. Table 1). Each dataset was manually curated to select biopsy or peripheral blood samples from Homo sapiens organism. For each study, we used the clinical and phenotypic descriptors reported by the corresponding original studies. We selected IF/TA samples confirmed by protocol biopsy as follows: high CADI scores at 3 months (n = 1), 6 months (n = 3), and ≥1 year (n = 21) from Naesens study^34^; moderate and severe IF/TA (n = 22) at 1 year from Kurian study^36^; nonspecific IF/TA (n = 17) from Hayde study^33^; IF/TA II and III (n = 18) ≥1 year from Rodder study^37^; IF/TA and IF/TA+i (n = 40) at 1 year from Park study^35^; high CADI scores at 1 year (n = 6) from our in-house study (GSE74313). The human samples used in our study were approved by Institutional Review Board approval at Mount Sinai Hospital (05-1013), and all methods and analyses were conducted in accordance with this approval. Informed consent was obtained from all subjects used in this study. Stables, normal and low CADI samples with matched time points post-transplant in each study were served as non-IF/TA.

We performed quality assessment of the raw microarray datasets obtained from GEO. All probe sets on three different platforms (Affymetrix Human Genome U133 Plus 2.0, Affymetrix Human Exon 1.0 ST, and Affymetrix Human Gene 1.0 ST) were re-annotated to the most recent NCBI Entrez Gene Identifiers (Gene IDs) by AILUN (ref. 41, http://ailun.ucsf.edu). We normalized each data set using quantile-quantile normalization^42^, and used Gene IDs to cross map genes among three different platforms.

### Microarray meta-analysis

We analyzed the six microarray datasets using two different meta-analysis methods: (1). Combining the effect sizes across the studies (equation 1). (2). Combining SAM q values across the studies (equation 2) (Fig. 1). The first method estimated the effect size for each gene in each data set. We summarized the effect size using a fixed effect inverse-variance model. We combined the study-specific effect sizes for each gene into one meta-effect size (*f*_*meta*_)using a linear combination of effect sizes (*f*_*i*_) by weighting each effect size by the inverse of the variance (*w*_*i*_) in the corresponding study. It indicates that studies with less intra-study variation (noise) contribute more to the overall estimate of meta-effect size^93^. We then calculated the false discovery rate (FDR)^94^ for multiple hypotheses testing for each gene (equation 1). We used the FDR ≤ 5% as cutoff for selecting significant genes. For the second meta-analysis method, we used Significance Analysis of Microarrays (SAM)^95^ to identify the significantly expressed genes with q-value < 10% between IF/TA and non-IF/TA phenotypes in each study. Then, we performed the Fisher-Exact test for calculating the probability of obtaining the number of studies with the significant genes by the hypergeometric distribution (equation 2). We used p ≤ 0.05 as cutoff for selecting significant genes.


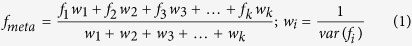



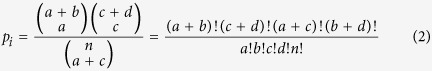


where *a* is the number of positive experiments for gene *i, b* is sum of the number of positive experiments for all other genes, *c* is the number of negative experiments for gene *i*, and *d* is the number of negative experiments for all other genes.

We used Ingenuity Pathway Analysis (IPA, http://www.ingenuity.com, Redwood city, CA) to identify the significant signaling pathways for the overlapped genes from two meta-analyses. We chose −log_10_P > 1.3 as a threshold for identifying significant pathways in IPA. We used BioGPS^49^,^50^ to identify the blood cell types in which the overlapped genes were highly expressed. A gene was highly expressed in a blood cell type if its expression in a given blood cell type was greater than 10 times its median expression over all tissues. We used hypergeometric test to determine whether the proportion of the overlapped genes in each cell type was statistically significant or not. The P-values from hypergeometric test were corrected for multiple hypotheses using Benjamini-Hochberg correction.

### Drug repositioning approach

We employed a computational drug repurposing approach to identify compounds predicted to ameliorate molecular states associated with IF/TA based on the concordance of drug-induced gene expression profiles from Connectivity Map (CMap)^40^. Each IF/TA signature was used to query CMap, a large library of drug induced transcriptional profiles^40^. We merged the 6,100 individual experiments into a single representative signature for the 1,309 unique small molecule compounds, according to the prototype-ranked list method^96^. For each unique compound, a modified Kolmogorov-Smirnov (KS) score was calculated^40^, summarizing the transcriptional relationship to the IF/TA signature, quantifying the tendency for those genes to be concordantly over- or under-expressed in the context of a given compound. Significance of individual scores was estimated by generating an empirical KS score distribution from the query network to 1,000 permuted drug signatures.

We hypothesized that if a IF/TA state is signified by a specific set of genome-wide transcriptional expression changes, and if exposure to a particular drug causes the reverse set of changes in a model cell line, then that drug has the potential to have a therapeutic effect on IF/TA^39^. We thus generated a ranked list of potential treatments for IF/TA from compounds predicted to induce an anti-correlated state to IF/TA (P ≤ 0.05)^39^,^97^ (Fig. 1).

We used Immune Pharmacology Map (IP-Map)^98^, a system-wide interaction map between drugs and immune cells by matching 1,309 drug perturbation profiles in the CMap^40^ to 304 immune cell state changes that we curated from the Immunological Genome compendia (ImmGen)^99^, to evaluate the significance of our predicted drug-cell type interactions to potentially underline the affected immune cell types. We generated random drug perturbation profiles for each compound and repeated the analysis 1,000 times for each immune cell state change. We used the generalized Pareto distribution to model the P-value distribution and calculated the extreme P-values calculation based on the distribution of permutation scores^100^. We reported adjusted P-values by performing the Benjamini Hochberg multi-hypothesis testing.

### Cell culture

Human kidney 2 (HK2) cells, which are immortalized proximal tubular epithelial cell (PTC) lines from normal adult human kidney, were cultured in RPMI-1640 medium (Corning). Upon reaching approximately 80% confluence, they were treated with indicated concentrations of kaempferol (EMD Millipore®), esculetin (Sigma®), or vehicle (0.15% DMSO for kaempferol and 0.5% DMSO for esculetin) in 0.5% nutrient medium. Cells were subsequently treated with 5 ng/ml TGF-β1 R&D Biosystems), 1.25 μM Wnt agonist AG-L-67051 (Santa Cruz Biotech), or respective vehicle at the indicated times. We assessed cell viability with kaempferol or esculetin treatment after 24 hours with Trypan Blue (MP Biomedicals LLC) using standard protocol^101^.

### Western blot

Cells were lysed using a 1% Triton lysis buffer with added protease, phosphorylation, and phosphatase inhibitors. Protein extracts were resolved using 8–10% Tris-glycine SDS-Polyacrylamide gels. The following antibodies (Cell Signaling) were used to detect proteins of interest: *anti-cyclin-d1, anti-P-smad3, anti-total smad3, anti-phospho-P65, anti-total P-65, anti-β-actin*, and *anti-GAPDH*. Indicated bands were quantified using Image-J or Image Studio Lite^102^,^103^.

### Real Time PCR

RNA was extracted using TRIzol Reagent (Life Technologies) and reversed transcribed using the Superscript III First-Strand Synthesis System (Life Technologies) following standard protocols. Real Time PCR (Applied Biosystems 7500) with the indicated primers was used to generate amplification curves that were analyzed using the ΔΔCT method with *GAPDH* as a house-keeping gene and universal RNA (human universal RNA; Agilent Technologies) as a control. Primer sequences used in PCR are provided in S. Table 4.

### Fibrotic mouse model

We used mouse unilateral ureteric obstruction (UUO) model to study the development of renal interstitial fibrosis. We obtained the *Balb/c* mice from Jackson Laboratory (Accession number: 000651)^89^. We followed the NIH Guide for the Care and Use of Laboratory Animals, and all methods and analyses were conducted in accordance with this guide. Eight week-old male *Balb/c* mice were administered 10 mg/kg/day of kaempferol (N = 5) or esculetin (N = 5) dissolved in 2.5% dimethylsulfoxide (DMSO) in Phosphate-buffered saline (PBS) beginning 2 days prior to undergoing UUO, and continued daily until the mice were sacrificed at 7 days post-UUO. Doses and modes of administration for the two compounds were chosen based on published literature^104^,^105^. Control mice were administered 2.5% DMSO in PBS (n = 6). Prior to the UUO, mice were anesthetized with an i.p. injection of 100 mg/kg ketamine and 16 mg/kg xylazine in PBS. 5–0 silk was used to completely ligate the left ureter 0.5 cm distal to the kidney with a total procedure time of 35–45 minutes. At 7 days post-UUO, the mice were perfused with PBS and tissue samples from the UUO kidney and contralateral kidney were collected for histology, RT-PCR, and Western Blot analysis.

### Histological evaluation

Kidney tissue was immersed in formalin followed by paraffin fixation on slides performed by the Biorepository and Pathology CORE facility at Mount Sinai. Slides were then deparaffinized and subjected to immunohistochemistry (IHC) using anti-collagen 1 (Southern Biotech, 1:50) or picrosirius red stain (Abcam) using anti-collagen 1 and anti-collagen 3. IHC was performed using the Vectastain ABC avidin-biotin kit and developed using DAB substrate (Vector Laboratories). Stained sections were visualized using the Zeiss Axioplan 2 microscope, and 10–15 randomly selected high-power fields (hpfs; original magnification x40) of the renal cortex were used to quantify the percentage of Picrosirius red stain or *COL1A1* (collagen 1) using Image J^102^,^103^.

All experimental protocols used in our study were approved by Icahn School of Medicine at Mount Sinai. We performed ANOVA for multiple comparisons and two-tailed T-test for unpaired comparisons with P ≤ 0.05 as significant level for experimental validation. Mean ± standard error of mean was reported for quantification results. All statistics were computed by R 2.15.1^106^.

## Additional Information

**How to cite this article**: Li, L. *et al*. Novel Therapeutics Identification for Fibrosis in Renal Allograft Using Integrative Informatics Approach. *Sci. Rep.* **7**, 39487; doi: 10.1038/srep39487 (2017).

**Publisher's note:** Springer Nature remains neutral with regard to jurisdictional claims in published maps and institutional affiliations.

## Supplementary Material

Supplementary Material
