# Supplementary material for: Novel Therapeutics Identification for Fibrosis in Renal Allograft Using Integrative Informatics Approach
Source: Sci Rep. 2017 Jan 4;7:39487. doi: 10.1038/srep39487 (PMC5209709; doi:10.1038/srep39487)
Supplement: Supplementary Material [file srep39487-s1.pdf]

Novel Therapeutics Identification for Fibrosis in Renal Allograft Using Integrative Informatics Approach

Li Li[1*], Ilana Greene[2*], Benjamin Readhead[1], Madhav C Menon[2], Brian A Kidd[1], Andrew V Uzilov[3], Chengguo Wei[2], Nimrod Philippe[2], Bernd Schroppel[2], John Cijiang He[2], Rong Chen[3], Joel T Dudley[1,4*#], Barbara Murphy[2*#]

1. Department of Genetics and Genomic Sciences, Icahn School of Medicine at Mount Sinai, 770 Lexington Ave., New York, NY 10065

2. Division of Nephrology, Department of Medicine, Icahn School of Medicine at Mount Sinai, One Gustave L. Levy Place, New York, NY 10029

3. Department of Genetics and Genomic Sciences, Icahn School of Medicine at Mount Sinai, 1255 5th Avenue, New York, NY 10029

4. Department of Health Policy and Research, Icahn School of Medicine at Mount Sinai, One Gustave L Levy Place, New York, NY 10029

*Equal contributions

# Correspondence to:

Barbara Murphy, MD.
Annenberg Building Floor 5-02
1468 Madison Avenue
New York, NY 10029
Tel: 212-241-4200
Fax: 212-241-1560
Barbara.murphy@mssm.edu

Joel Dudley, PhD.
770 Lexington Ave
15th Floor
New York, NY 10065
Tel: 212-659-8656
Joel.dudley@mssm.edu

Microarray data repository: GSE74313

SUPPLEMENTAL MATERIALS

S. Table 1: 6 microarray datasets including 5 public available datasets from GEO.

S. Table 2A: A list of 85 genes from two meta-analysis methods.

S. Table 2B: Canonical pathways for 85 genes specific to IF/TA.

S. Table 3: Rank order of the significant compounds (p<=0.05) driven by 85 IF/TA genes.

S. Table 4: A list of primers used for RT PCR experiments.

S. Figure 1: Heatmap of 85 genes' expression across 6 different datasets.

S. Figure 2: 1309 compounds ranking score driven by 85 IF/TA genes.

S. Figure 3: Key genes out of 85 IF/TA genes drive the identification of kaempferol and esculetin.

S. Figure 4: HK2 cell viability by Kaempferol and Esculetin.

S. Figure 5: Pro-fibrotic mediators on control samples by western blot.

S. Figure 6: Venn diagram of overlapped drugs across 6 drug lists specific to each dataset.

S. Table 1: 6 microarray datasets including 5 public available datasets from GEO.

GSE	Platform	GPL ID	Author, Journal, Yr	Tissue	STA	CAI (CADI/IFTA)	Total
GSE12187	HU133+	GPL570	Kurian et al, PlosOne, 2009	PBL	26	22	48
GSE25902	HU133+	GPL570	Naesens et al, KI, 2010	BX	48	25*	73
GSE44131	HG1.0ST	GPL6244	Hayde et al, cJASN, 2013	BX	12	17	29
GSE9493	HU133+	GPL570	Rodder et al, AJT, 2009	BX	20	18	38
GSE22459	HU133+	GPL570	Park et al, JASN, 2010	BX	25	40	65
GSE74313	HT1.0ST	GPL5188	In-House Data, 2016	BX	16	6*	22

S. Table 2A: A list of 85 genes from two meta-analysis methods.

GeneID	Symbol	Meta EffectSize	FDR (Meta-EffectSize)	FisherExactP (Meta-SAM)	Naesens.EffectSize	Naesens.EffectSizeP	Naesens.SAMq	Hayde.EffectSize	Hayde.EffectSizeP	Hayde.SAMq	Hayde.EffectSize
834	CASP1	1.48	1.50E-04	5.00E-02	1.96	1.72E-38	0.00E+00	1.11	4.38E-01	5.99E-01	1.76
841	CASP8	1.13	4.46E-02	3.04E-02	1.16	2.40E-01	1.30E-02	-1.00	9.45E-01	9.90E-01	1.26
867	CBL	1.10	2.85E-02	5.00E-02	1.19	1.34E-02	1.30E-02	1.09	2.59E-02	2.74E-01	1.33
915	CD3D	1.64	4.92E-02	5.00E-02	3.76	2.87E-07	1.69E-05	1.28	1.41E-01	4.22E-01	2.15
962	CD48	1.45	1.12E-03	5.00E-02	2.00	2.00E-01	0.00E+00	1.41	4.44E-02	3.15E-01	1.40
963	CD53	1.28	5.00E-05	7.06E-03	1.81	2.58E-02	1.69E-05	1.25	1.59E-01	4.38E-01	1.69
1234	CCR5	1.61	3.58E-03	5.00E-02	3.63	5.75E-14	0.00E+00	1.04	4.10E-01	6.47E-01	2.15
1520	CTSS	1.66	1.34E-02	7.06E-03	2.45	3.65E-22	0.00E+00	1.07	7.23E-01	6.99E-01	2.94
1536	CYBB	1.17	4.47E-03	5.00E-02	1.53	1.47E-02	0.00E+00	1.18	3.42E-01	5.56E-01	1.40
1901	S1PR1	1.10	3.59E-02	5.00E-02	1.20	1.65E-01	4.14E-02	1.02	6.49E-01	6.76E-01	1.27
2124	EVI2B	1.65	4.92E-03	5.00E-02	2.93	8.67E-15	0.00E+00	1.44	4.29E-02	3.13E-01	2.06
2206	MS4A2	1.26	3.19E-02	5.00E-02	1.28	3.99E-04	1.06E-02	1.66	2.11E-05	8.13E-02	1.31
2212	FCGR2A	1.19	0.00E+00	5.00E-02	1.47	4.67E-02	1.69E-05	1.18	3.53E-01	5.61E-01	1.29
2213	FCGR2B	1.63	2.09E-02	5.00E-02	2.68	9.90E-08	1.69E-05	1.81	8.99E-06	7.12E-02	1.72
2359	FPR3	1.33	3.80E-04	5.00E-02	1.54	1.55E-03	1.63E-03	1.12	4.59E-01	6.08E-01	1.74
2533	FYB	1.37	1.13E-03	5.00E-02	1.82	2.11E-19	8.00E-05	1.24	2.23E-01	4.87E-01	1.48
2857	GPR34	1.40	2.62E-02	5.00E-02	1.55	9.10E-03	4.12E-02	1.07	6.65E-01	6.82E-01	2.20
3055	HCK	1.39	1.45E-02	5.00E-02	1.90	1.20E-04	3.34E-03	1.23	8.47E-03	2.07E-01	1.92
3115	HLA-DPB1	1.49	1.14E-02	3.04E-02	3.11	3.52E-09	0.00E+00	1.20	5.57E-03	3.77E-01	2.48
3275	PRMT2	1.05	8.00E-05	5.00E-02	1.05	3.97E-01	1.99E-02	-1.01	9.10E-01	7.40E-01	1.04
3313	HSPA9	-1.13	4.63E-02	3.04E-02	-1.26	1.16E-03	3.38E-03	-1.00	9.57E-01	9.90E-01	-1.12
3431	SP110	1.22	2.25E-02	5.00E-02	1.36	9.51E-19	1.69E-05	1.08	3.21E-01	5.45E-01	1.25
3575	IL7R	1.66	3.79E-02	5.00E-02	2.21	8.91E-02	1.69E-05	1.28	3.46E-01	5.58E-01	2.43
3604	TNFRSF9	1.18	3.23E-02	5.00E-02	1.30	9.79E-04	3.72E-02	-1.09	4.22E-01	5.93E-01	1.29
3669	ISG20	1.51	1.32E-02	7.06E-03	1.78	1.91E-04	2.91E-02	1.14	5.57E-02	3.34E-01	2.52
3718	JAK3	1.05	2.57E-02	5.00E-02	1.20	2.52E-01	0.00E+00	1.07	5.07E-01	6.28E-01	1.15
4063	LY9	1.26	5.00E-02	5.00E-02	1.65	5.04E-03	1.71E-03	1.15	1.45E-01	4.25E-01	1.34
4069	LYZ	1.58	2.01E-02	5.00E-02	2.53	7.98E-04	0.00E+00	1.51	6.43E-02	3.43E-01	3.31
4170	MCL1	1.05	0.00E+00	5.00E-02	-1.06	4.25E-01	5.82E-02	-1.03	6.45E-01	6.75E-01	1.07
4332	MNDA	1.55	1.86E-03	5.00E-02	1.82	2.89E-06	2.70E-04	1.22	3.31E-02	2.90E-01	2.12
4688	NCF2	1.48	1.06E-02	5.00E-02	1.69	8.45E-04	1.08E-02	1.11	4.30E-01	5.96E-01	2.19

4689	NCF4	1.50	2.38E-03	5.00E-02	1.66	2.10E-07	1.70E-03	1.34	1.96E-02	2.55E-01	2.30
5733	PTGER3	-1.19	6.76E-03	5.00E-02	-1.49	1.34E-09	5.92E-03	-1.46	1.32E-03	1.44E-01	-1.21
5788	PTPRC	1.37	1.82E-02	5.00E-02	2.40	1.24E-03	0.00E+00	1.39	1.34E-01	4.15E-01	1.33
5791	PTPRE	1.14	9.11E-03	5.00E-02	1.41	4.91E-02	0.00E+00	1.22	9.95E-03	2.13E-01	1.35
6039	RNASE6	1.78	7.46E-03	5.00E-02	2.77	9.33E-13	0.00E+00	1.33	2.99E-02	2.84E-01	2.64
6402	SELL	1.55	2.80E-02	5.00E-02	3.43	6.60E-08	0.00E+00	1.21	2.48E-01	5.03E-01	1.26
6584	SLC22A5	-1.06	6.00E-05	5.00E-02	-1.41	1.59E-01	7.00E-05	-1.03	7.95E-01	7.16E-01	-1.67
7068	THRB	-1.16	1.06E-02	5.00E-02	-1.21	5.22E-05	5.20E-03	-1.19	9.08E-03	2.08E-01	-1.29
7082	TJP1	-1.03	1.80E-04	5.00E-02	1.03	8.79E-01	5.01E-02	-1.08	1.71E-01	4.46E-01	1.07
7097	TLR2	1.39	2.79E-02	5.00E-02	1.26	6.76E-02	1.32E-01	1.08	3.94E-01	5.81E-01	2.14
7099	TLR4	1.31	4.25E-03	5.00E-02	1.14	1.87E-02	1.38E-01	1.21	3.18E-02	2.89E-01	1.69
7273	TTN	-1.06	0.00E+00	5.00E-02	-1.18	2.54E-02	5.46E-02	1.38	5.37E-01	6.38E-01	-1.13
7756	ZNF207	1.04	4.26E-02	5.00E-02	1.13	1.46E-02	1.78E-04	-1.01	7.06E-01	6.40E-01	1.01
7805	LAPTM5	1.48	9.78E-03	5.00E-02	2.21	4.40E-10	4.32E-04	1.17	3.51E-01	5.61E-01	2.34
7903	ST8SIA4	1.40	9.00E-05	5.00E-02	1.77	2.91E-07	1.69E-05	1.20	2.01E-01	4.71E-01	1.53
7940	LST1	1.46	2.75E-03	5.00E-02	2.39	4.64E-41	0.00E+00	1.33	5.50E-09	1.22E-01	1.54
8832	CD84	1.24	1.26E-03	7.06E-03	1.33	8.27E-03	4.40E-05	1.26	8.24E-02	3.65E-01	1.45
8945	BTRC	-1.01	1.66E-02	5.00E-02	-1.02	7.63E-01	7.19E-02	-1.02	6.02E-01	6.61E-01	-1.02
9111	NMI	1.26	7.46E-03	5.00E-02	1.38	1.56E-05	1.41E-03	-1.02	8.59E-01	7.30E-01	1.71
9332	CD163	1.54	2.84E-03	5.00E-02	1.43	1.49E-04	3.62E-02	1.18	2.90E-01	5.29E-01	2.93
9447	AIM2	1.59	5.40E-04	5.00E-02	2.27	2.23E-04	4.64E-03	1.15	1.65E-01	4.42E-01	2.08
9938	ARHGAP25	1.18	1.48E-03	5.00E-02	1.74	1.69E-02	1.69E-05	1.09	2.86E-01	5.27E-01	1.24
10154	PLXNC1	1.15	0.00E+00	5.00E-02	1.18	5.20E-03	9.81E-03	1.13	2.08E-01	4.77E-01	-1.02
10261	IGSF6	1.70	2.83E-02	7.06E-03	1.51	1.11E-02	4.64E-02	1.32	1.23E-01	4.04E-01	2.49
10673	TNFSF13B	1.50	1.53E-02	7.06E-03	1.88	2.87E-12	1.69E-05	1.16	2.52E-01	5.05E-01	1.75
11010	GLIPR1	1.41	2.88E-02	5.00E-02	1.56	1.61E-04	1.60E-04	1.10	5.71E-01	6.50E-01	2.17
11031	RAB31	1.28	2.31E-02	5.00E-02	1.14	2.63E-01	9.95E-03	1.24	1.72E-02	2.44E-01	1.62
11314	CD300A	1.14	3.00E-05	5.00E-02	1.18	5.01E-01	1.69E-05	1.12	8.43E-02	3.68E-01	1.32
25939	SAMHD1	1.37	9.87E-03	5.00E-02	1.65	4.66E-06	0.00E+00	1.16	3.70E-01	5.71E-01	1.98
27074	LAMP3	1.63	4.46E-02	5.00E-02	3.79	2.41E-08	0.00E+00	1.13	2.30E-01	4.91E-01	1.57
27128	CYTH4	1.18	4.06E-02	5.00E-02	1.13	6.98E-01	2.70E-03	1.32	2.19E-02	2.63E-01	1.06
51192	CKLF	1.44	1.97E-02	3.04E-02	1.49	1.67E-11	8.01E-04	-	-	-	1.97
51279	C1RL	1.06	4.33E-03	5.00E-02	1.17	6.70E-02	4.20E-02	1.06	5.36E-01	6.38E-01	1.21
51311	TLR8	1.36	9.85E-03	5.00E-02	1.35	3.58E-01	4.40E-05	1.12	1.86E-01	4.59E-01	1.67
51338	MS4A4A	1.53	9.32E-03	5.00E-02	1.74	6.36E-10	4.32E-04	1.09	6.34E-01	6.71E-01	2.79

51363	CHST15	1.47	4.34E-02	5.00E-02	1.92	3.87E-08	0.00E+00	1.15	1.98E-01	4.69E-01	1.91
53346	TM6SF1	1.31	1.32E-02	5.00E-02	1.35	3.54E-03	2.65E-02	1.12	3.67E-01	5.69E-01	1.96
55711	FAR2	1.28	1.49E-02	5.00E-02	1.31	5.38E-04	2.01E-02	1.06	4.24E-01	5.93E-01	1.41
55843	ARHGAP15	1.51	1.37E-02	5.00E-02	2.57	2.15E-11	0.00E+00	1.47	8.02E-03	2.05E-01	1.81
58475	MS4A7	1.71	3.90E-02	3.04E-02	2.06	1.30E-25	0.00E+00	-	-	-	2.49
58484	NLRC4	1.19	2.21E-02	5.00E-02	1.28	4.41E-03	1.25E-02	1.09	9.03E-02	3.74E-01	1.62
64087	MCCC2	-1.06	0.00E+00	5.00E-02	-1.09	4.52E-01	4.49E-03	-1.11	1.16E-01	5.14E-01	-1.23
64231	MS4A6A	1.57	1.29E-03	7.06E-03	2.20	6.48E-07	0.00E+00	1.37	2.72E-02	2.78E-01	2.11
64407	RGS18	1.41	1.45E-02	5.00E-02	2.29	3.59E-06	2.70E-04	1.06	4.16E-01	5.91E-01	1.43
64744	SMAP2	1.30	1.35E-02	7.06E-03	1.49	3.61E-04	7.25E-03	1.16	1.12E-01	3.94E-01	1.43
120425	AMICA1	1.79	3.17E-02	5.00E-02	3.16	1.28E-12	0.00E+00	1.43	1.74E-02	2.46E-01	1.67
137835	TMEM71	1.41	7.54E-03	5.00E-02	1.83	1.17E-05	6.64E-04	1.17	1.82E-01	4.56E-01	1.64
205717	KIAA2018	-1.08	0.00E+00	5.00E-02	-1.09	3.63E-02	6.54E-02	-1.06	1.63E-01	4.42E-01	-1.19
219972	MPEG1	1.71	2.59E-02	5.00E-02	3.01	3.97E-19	0.00E+00	1.42	5.55E-02	3.34E-01	2.14
255488	RNF144B	1.15	0.00E+00	5.00E-02	1.25	1.03E-01	1.39E-02	-1.05	7.60E-01	7.08E-01	1.21
256380	SCML4	1.23	3.60E-02	5.00E-02	1.78	5.61E-07	0.00E+00	1.08	2.07E-01	4.76E-01	1.01
257106	ARHGAP30	1.09	2.40E-04	7.06E-03	1.42	2.26E-01	1.69E-05	1.08	3.01E-01	5.34E-01	1.22
728970	PPP1R2P4	1.58	3.20E-02	1.38E-02	2.38	1.60E-13	0.00E+00	-	-	-	1.35
729230	CCR2	1.40	1.00E-04	5.00E-02	2.38	2.06E-01	0.00E+00	1.64	1.43E-05	1.47E-01	1.56

Rodder.EffectSizeP	Rodder.SAMq	Park.EffectSize	Park.EffectSizeP	Park.SAMq	Kurian.EffectSize	Kurian.EffectSizeP	Kurian.SAMq	InHouse.EffectSize	InHouse.EffectSizeP	InHouse.SAMq
2.44E-11	7.02E-03	1.49	1.622E-17	7.14E-02	1.29	4.70E-07	9.26E-02	1.32	4.69E-26	4.11E-01
3.28E-04	4.19E-02	1.14	0.0276366	5.98E-01	1.22	2.15E-03	9.26E-02	1.09	3.65E-08	4.14E-01
6.02E-02	4.69E-05	1.02	0.7342042	3.48E-01	1.29	3.16E-03	9.26E-02	1.08	1.78E-26	5.87E-01
7.32E-03	1.28E-02	2.03	0.0002012	8.26E-02	-1.14	5.19E-01	3.45E-01	1.36	7.68E-08	4.77E-01
2.99E-01	8.06E-03	1.78	9.95E-08	7.14E-02	1.12	6.82E-01	1.05E-01	1.37	4.96E-29	3.93E-01
8.58E-03	1.11E-03	1.45	0.0006462	7.14E-02	1.15	6.02E-02	9.26E-02	1.24	1.94E-43	4.28E-01
3.23E-03	8.47E-03	1.93	9.994E-05	7.14E-02	1.49	2.48E-02	9.56E-02	1.13	3.14E-07	5.88E-01
7.54E-08	4.69E-05	1.76	2.667E-10	7.14E-02	1.30	2.24E-03	9.26E-02	1.30	5.02E-33	1.33E-01
8.46E-02	1.32E-03	1.21	0.0616804	8.26E-02	1.04	5.13E-01	3.36E-01	1.18	2.75E-49	4.10E-01
2.18E-02	2.83E-02	1.12	0.1354724	5.64E-01	-1.16	3.74E-01	9.26E-02	1.12	7.52E-12	5.23E-01
1.51E-05	3.05E-03	1.93	1.065E-09	7.14E-02	1.15	1.47E-02	9.33E-02	1.20	6.38E-09	4.32E-01
1.53E-01	4.31E-02	1.05	0.6968614	6.72E-01	1.03	6.73E-01	3.15E-01	1.36	2.03E-07	2.37E-01
4.53E-01	4.09E-03	1.25	0.271527	7.14E-02	1.27	4.06E-01	9.26E-02	1.19	5.48E-13	3.11E-01
6.24E-03	1.72E-02	1.45	0.0072816	2.89E-01	1.49	8.04E-02	1.30E-01	1.18	2.25E-04	2.35E-01
8.07E-03	4.94E-03	1.49	5.894E-06	7.66E-02	1.02	8.54E-01	9.90E-02	1.31	1.48E-16	3.94E-01
1.68E-04	8.53E-03	1.49	1.085E-07	7.14E-02	1.14	2.40E-03	9.37E-02	1.19	4.38E-24	4.27E-01
5.12E-07	3.59E-04	1.43	2.277E-05	7.14E-02	1.23	1.54E-01	1.74E-01	1.28	6.83E-12	3.12E-01
2.50E-04	3.83E-03	1.20	0.048177	4.85E-01	2.07	4.83E-03	9.26E-02	1.11	1.85E-06	5.45E-01
2.37E-04	2.17E-03	1.81	3.453E-05	7.14E-02	-1.00	9.68E-01	9.90E-01	1.17	4.36E-13	4.99E-01
4.21E-01	5.38E-02	1.03	0.4584499	3.06E-01	1.17	1.69E-02	9.26E-02	1.05	5.57E-09	6.23E-01
1.21E-01	1.47E-02	-1.11	5.456E-05	2.26E-01	-1.49	1.34E-05	9.26E-02	-1.04	1.50E-18	6.12E-01
1.32E-03	1.18E-02	1.17	2.227E-07	2.58E-01	1.46	9.03E-10	9.26E-02	1.06	3.13E-10	6.02E-01
1.76E-04	2.86E-03	2.79	1.738E-09	7.14E-02	1.13	3.85E-01	2.88E-01	1.26	9.86E-24	5.02E-01
7.82E-03	5.57E-02	1.23	0.1955519	2.37E-01	1.34	1.15E-01	9.26E-02	1.14	3.87E-03	3.64E-01
1.55E-05	2.69E-03	2.15	2.511E-07	7.14E-02	1.39	1.38E-05	9.26E-02	1.06	1.01E-03	5.65E-01
3.62E-01	1.28E-02	1.07	0.4047723	1.90E-01	1.02	8.97E-01	9.26E-02	1.05	6.53E-04	5.00E-01
5.57E-02	5.90E-02	1.71	9.543E-05	7.14E-02	1.13	1.99E-01	1.00E-01	1.07	2.58E-04	4.88E-01
1.17E-06	1.72E-03	1.96	0.0034934	7.69E-02	-1.07	2.82E-01	2.50E-01	1.25	3.54E-19	3.73E-01
4.68E-01	9.19E-02	1.09	0.0952688	3.97E-01	-1.04	6.76E-01	9.26E-02	1.06	6.70E-08	6.39E-01
3.33E-04	3.33E-03	1.58	0.0002726	1.02E-01	2.21	6.64E-04	9.26E-02	1.25	1.60E-11	5.23E-01
2.79E-05	1.32E-03	1.50	0.0008793	1.53E-01	1.78	3.37E-03	9.26E-02	1.22	2.05E-11	2.88E-01

5.19E-04	3.97E-04	1.27	0.0031913	3.06E-01	2.16	3.72E-05	9.26E-02	1.20	1.36E-07	4.53E-01
5.56E-02	9.24E-03	-1.13	0.0026458	4.20E-01	-1.07	3.43E-02	9.26E-02	-1.09	8.87E-14	4.93E-01
8.03E-03	2.45E-02	1.81	1.277E-13	7.14E-02	-1.01	8.40E-01	1.43E-01	1.24	1.29E-48	3.64E-01
6.42E-03	6.13E-03	1.10	0.0981638	4.38E-01	1.15	1.87E-01	9.26E-02	1.10	1.15E-08	3.73E-01
4.94E-07	2.36E-04	1.69	0.0001786	8.74E-02	1.68	2.94E-02	9.81E-02	1.29	4.34E-41	4.27E-01
4.54E-01	2.74E-01	1.77	9.604E-05	7.17E-02	1.47	1.04E-03	9.26E-02	1.20	5.45E-15	5.19E-01
1.48E-01	7.65E-05	-1.05	0.4552738	6.76E-01	-1.02	9.44E-01	9.26E-02	-1.06	1.75E-06	5.82E-01
3.37E-04	5.16E-03	-1.19	1.767E-06	2.70E-01	-1.10	1.19E-01	9.26E-02	-1.07	1.49E-14	7.06E-01
6.76E-01	8.35E-02	1.03	0.3948668	7.44E-01	-1.09	5.60E-01	9.26E-02	-1.03	7.45E-07	5.98E-01
7.59E-07	4.46E-04	1.49	9.427E-05	8.41E-02	1.96	3.44E-03	9.26E-02	1.13	1.73E-05	5.69E-01
9.55E-11	2.43E-03	1.27	0.0010152	7.14E-02	1.67	9.71E-08	9.26E-02	1.15	7.23E-51	4.75E-01
4.31E-01	3.81E-03	-1.09	0.0497088	6.30E-01	-1.01	9.43E-01	9.26E-02	-1.06	9.39E-64	5.42E-01
9.03E-01	3.57E-02	1.05	0.0044177	4.92E-01	1.07	5.13E-01	9.26E-02	1.04	3.63E-47	6.56E-01
5.33E-09	3.29E-04	1.56	0.0006293	7.14E-02	1.09	9.88E-02	9.26E-02	1.18	2.89E-14	4.47E-01
2.64E-07	3.71E-03	1.29	0.0002549	1.94E-01	1.67	1.26E-02	9.26E-02	1.26	9.34E-13	3.63E-01
4.70E-08	4.12E-03	1.34	9.33E-11	1.22E-01	1.30	1.35E-09	9.26E-02	1.19	4.38E-03	4.29E-01
1.26E-08	3.64E-03	1.20	0.0359696	7.14E-02	1.05	2.05E-01	9.26E-02	1.26	6.09E-23	3.82E-01
9.11E-01	1.04E-02	1.01	0.8713032	5.32E-01	-1.06	1.64E-01	9.26E-02	-1.00	2.14E-01	7.46E-01
5.04E-06	1.97E-03	1.25	0.0014231	2.28E-01	1.28	3.25E-03	9.26E-02	1.17	8.74E-18	5.03E-01
1.84E-10	4.69E-05	1.55	8.55E-08	1.10E-01	1.62	1.89E-05	9.26E-02	1.25	3.72E-36	3.01E-01
5.35E-04	4.17E-03	1.58	0.0100464	3.04E-01	2.22	2.75E-03	9.26E-02	1.42	2.29E-14	3.90E-01
1.41E-02	3.97E-02	1.24	0.366159	7.69E-02	1.33	6.16E-03	9.26E-02	1.17	6.31E-19	5.66E-01
9.09E-01	1.69E-02	1.09	0.2974857	5.23E-01	1.27	3.81E-02	9.26E-02	1.15	5.12E-19	3.73E-01
1.10E-05	7.57E-04	2.15	1.1E-05	7.14E-02	2.55	4.17E-03	9.26E-02	1.20	2.76E-10	5.49E-01
6.15E-04	8.19E-03	1.70	1.6E-09	7.14E-02	1.47	5.60E-02	9.26E-02	1.23	1.97E-07	4.21E-01
3.13E-25	2.02E-04	1.41	1.425E-11	7.14E-02	1.24	1.27E-04	9.26E-02	1.15	4.62E-10	4.50E-01
1.97E-10	1.05E-03	1.28	1.56E-06	1.75E-01	1.43	1.45E-05	9.26E-02	1.08	3.26E-03	6.23E-01
1.45E-01	6.68E-03	1.24	0.0750109	1.62E-01	1.40	8.78E-02	9.26E-02	1.12	2.90E-05	3.95E-01
4.40E-06	1.84E-04	1.39	3.053E-10	1.22E-01	1.24	2.02E-02	9.26E-02	1.11	1.21E-44	5.07E-01
1.21E-01	9.45E-02	2.45	0.0002425	8.26E-02	1.44	5.21E-02	1.11E-01	1.12	3.05E-06	6.05E-01
7.67E-01	1.72E-02	1.13	0.2568122	7.14E-02	1.34	7.64E-05	9.26E-02	1.11	1.29E-06	4.64E-01
3.59E-19	2.02E-04	1.19	9.94E-05	3.00E-01	1.61	9.89E-09	9.26E-02	1.14	4.23E-17	4.88E-01
5.79E-01	1.70E-03	1.14	0.0727428	5.22E-01	1.38	4.23E-01	9.26E-02	1.06	1.27E-03	6.49E-01
1.43E-01	9.68E-04	1.40	0.0004105	1.86E-01	2.05	1.43E-05	9.26E-02	1.25	3.45E-04	5.17E-01
5.79E-10	4.69E-05	1.49	4.503E-11	7.14E-02	1.26	3.54E-01	9.26E-02	1.25	6.42E-25	3.64E-01

1.39E-06	8.64E-04	1.24	0.0322117	4.36E-01	2.46	3.05E-03	9.26E-02	1.09	1.16E-09	5.07E-01
2.71E-05	1.75E-03	1.12	0.4090502	7.42E-01	1.57	4.95E-03	9.26E-02	1.19	1.42E-10	4.72E-01
2.40E-04	1.74E-02	1.33	0.000259	2.71E-01	1.94	4.69E-05	9.26E-02	1.09	5.41E-11	5.59E-01
3.30E-03	1.16E-02	1.64	0.0001269	8.26E-02	1.02	8.17E-01	4.47E-01	1.23	5.78E-42	4.57E-01
7.54E-11	1.08E-04	1.90	3.438E-14	7.14E-02	1.18	2.81E-01	1.05E-01	1.28	1.87E-23	3.73E-01
2.31E-04	1.83E-02	1.11	0.561277	3.47E-01	1.44	3.34E-01	9.26E-02	1.14	2.11E-06	5.07E-01
7.85E-02	2.39E-03	-1.08	0.0935367	5.11E-01	-1.11	2.62E-01	9.26E-02	-1.06	1.28E-11	4.88E-01
1.15E-07	1.47E-04	1.46	2.194E-05	7.14E-02	1.53	2.39E-10	9.26E-02	1.24	4.27E-28	1.33E-01
4.64E-02	6.39E-02	1.49	0.0001208	8.77E-02	1.14	6.01E-01	3.76E-01	1.45	9.76E-11	3.01E-01
3.05E-02	5.27E-02	1.31	3.445E-05	8.61E-02	1.70	7.93E-04	9.26E-02	1.11	3.27E-09	5.07E-01
1.30E-02	2.51E-02	1.85	0.0003543	9.93E-02	2.09	2.27E-03	9.26E-02	1.23	9.21E-14	4.80E-01
5.91E-02	5.79E-02	1.63	0.0191466	3.47E-01	1.49	1.13E-03	9.26E-02	1.23	4.21E-06	5.40E-01
9.21E-02	5.81E-03	-1.06	0.0884065	4.01E-01	1.01	9.28E-01	9.26E-02	-1.08	7.35E-13	4.24E-01
1.02E-05	6.13E-03	1.60	1.541E-06	8.74E-02	1.50	9.51E-03	1.19E-01	1.19	1.55E-11	6.11E-01
7.71E-02	2.18E-02	1.08	0.2805431	7.68E-01	1.20	1.27E-01	9.26E-02	1.15	5.56E-30	4.09E-01
9.45E-01	1.21E-01	1.42	0.0034641	7.14E-02	1.28	3.05E-08	9.26E-02	1.10	4.89E-05	4.55E-01
1.93E-01	1.56E-02	1.20	0.2426463	7.66E-02	1.16	2.42E-01	9.26E-02	1.08	2.24E-06	4.37E-01
5.76E-02	5.14E-02	1.54	8.982E-08	7.14E-02	1.21	2.01E-01	2.72E-01	-	-	-
2.15E-02	1.29E-02	1.38	0.2610982	1.04E-01	1.87	1.48E-02	9.26E-02	1.37	8.89E-23	3.39E-01

S. Table 2B: Canonical pathways for 85 genes specific to IF/TA.

**Pathways**	**P-value**	**Genes**
TREM1 Signaling	6.0256E-07	TLR2,TLR4,TLR8,CASP1,FCGR2B,NLRC4
Production of Nitric Oxide and Reactive Oxygen Species in Macrophages	9.33254E-06	TLR2,TLR4,LYZ,NCF2,CYBB,NCF4,JAK3
Primary Immunodeficiency Signaling	6.16595E-05	IL7R,PTPRC,JAK3,CD3D
**NF-κB Signaling**	7.94328E-05	TLR2,TLR4,TLR8,BTRC,CASP8,TNFSF13B
phagosome formation	9.54993E-05	TLR2,TLR4,FCGR2A,TLR8,FCGR2B
Role of Pattern Recognition Receptors in Recognition of Bacteria and Viruses	0.000177828	TLR2,TLR4,TLR8,CASP1,NLRC4
Systemic Lupus Erythematosus Signaling	0.000295121	PTPRC,CBL,FCGR2A,FCGR2B,CD3D,TNFSF13B
Altered T Cell and B Cell Signaling in Rheumatoid Arthritis	0.00047863	TLR2,TLR4,TLR8,TNFSF13B
**Communication between Innate and Adaptive Immune Cells**	0.000537032	TLR2,TLR4,TLR8,TNFSF13B
**Fcγ Receptor-mediated Phagocytosis in Macrophages and Monocytes**	0.000588844	CBL,FCGR2A,HCK,FYB
MSP-RON Signaling Pathway	0.000891251	TLR2,TLR4,CCR2
**PI3K Signaling in B Lymphocytes**	0.001949845	PTPRC,TLR4,CBL,FCGR2B
IL-10 Signaling	0.002754229	CCR5,FCGR2A,FCGR2B
Colorectal Cancer Metastasis Signaling	0.002884032	TLR2,TLR4,PTGER3,TLR8,JAK3
**Toll-like Receptor Signaling**	0.003388442	TLR2,TLR4,TLR8
Tec Kinase Signaling	0.004168694	TLR4,MS4A2,HCK,JAK3
**Role of NFAT in Regulation of the Immune Response**	**0.005495409**	**FCGR2A,MS4A2,FCGR2B,CD3D**
**Dendritic Cell Maturation**	0.006309573	**TLR2,TLR4,FCGR2A,FCGR2B**
**T Cell Receptor Signaling**	0.007585776	PTPRC,CBL,CD3D
Cytotoxic T Lymphocyte-mediated Apoptosis of Target Cells	0.007585776	CASP8,CD3D
**B Cell Development**	0.008709636	IL7R,PTPRC
fMLP Signaling in Neutrophils	0.01	FPR3,NCF2,CYBB
Sphingosine-1-phosphate Signaling	0.01	CASP1,S1PR1,CASP8
iNOS Signaling	0.014125375	TLR4,JAK3
IL-12 Signaling and Production in Macrophages	0.018197009	TLR2,TLR4,LYZ
Glucocorticoid Receptor Signaling	0.026915348	HSPA9,CD163,JAK3,CD3D

S. Table 2B: Canonical pathways for 85 genes specific to IF/TA.

**Pathways**	**P-value**	**Genes**
TREM1 Signaling	6.0256E-07	TLR2,TLR4,TLR8,CASP1,FCGR2B,NLRC4
Production of Nitric Oxide and Reactive Oxygen Species in Macrophages	9.33254E-06	TLR2,TLR4,LYZ,NCF2,CYBB,NCF4,JAK3
Primary Immunodeficiency Signaling	6.16595E-05	IL7R,PTPRC,JAK3,CD3D
**NF-κB Signaling**	7.94328E-05	TLR2,TLR4,TLR8,BTRC,CASP8,TNFSF13B
phagosome formation	9.54993E-05	TLR2,TLR4,FCGR2A,TLR8,FCGR2B
Role of Pattern Recognition Receptors in Recognition of Bacteria and Viruses	0.000177828	TLR2,TLR4,TLR8,CASP1,NLRC4
Systemic Lupus Erythematosus Signaling	0.000295121	PTPRC,CBL,FCGR2A,FCGR2B,CD3D,TNFSF13B
Altered T Cell and B Cell Signaling in Rheumatoid Arthritis	0.00047863	TLR2,TLR4,TLR8,TNFSF13B
**Communication between Innate and Adaptive Immune Cells**	0.000537032	TLR2,TLR4,TLR8,TNFSF13B
**Fcγ Receptor-mediated Phagocytosis in Macrophages and Monocytes**	0.000588844	CBL,FCGR2A,HCK,FYB
MSP-RON Signaling Pathway	0.000891251	TLR2,TLR4,CCR2
**PI3K Signaling in B Lymphocytes**	0.001949845	PTPRC,TLR4,CBL,FCGR2B
IL-10 Signaling	0.002754229	CCR5,FCGR2A,FCGR2B
Colorectal Cancer Metastasis Signaling	0.002884032	TLR2,TLR4,PTGER3,TLR8,JAK3
**Toll-like Receptor Signaling**	0.003388442	TLR2,TLR4,TLR8
Tec Kinase Signaling	0.004168694	TLR4,MS4A2,HCK,JAK3
**Role of NFAT in Regulation of the Immune Response**	**0.005495409**	**FCGR2A,MS4A2,FCGR2B,CD3D**
**Dendritic Cell Maturation**	0.006309573	**TLR2,TLR4,FCGR2A,FCGR2B**
**T Cell Receptor Signaling**	0.007585776	PTPRC,CBL,CD3D
Cytotoxic T Lymphocyte-mediated Apoptosis of Target Cells	0.007585776	CASP8,CD3D
**B Cell Development**	0.008709636	IL7R,PTPRC
fMLP Signaling in Neutrophils	0.01	FPR3,NCF2,CYBB
Sphingosine-1-phosphate Signaling	0.01	CASP1,S1PR1,CASP8
iNOS Signaling	0.014125375	TLR4,JAK3
IL-12 Signaling and Production in Macrophages	0.018197009	TLR2,TLR4,LYZ
Glucocorticoid Receptor Signaling	0.026915348	HSPA9,CD163,JAK3,CD3D

Role of JAK1 and JAK3 in γc Cytokine Signaling	0.027542287	IL7R,JAK3
CCR5 Signaling in Macrophages	0.033113112	CCR5,CD3D
Role of Macrophages, Fibroblasts and Endothelial Cells in Rheumatoid Arthritis	0.033884416	TLR2,TLR4,TLR8,TNFSF13B
Granulocyte Adhesion and Diapedesis	0.035481339	FPR3,SELL,CKLF
**B Cell Receptor Signaling**	0.036307805	PTPRC,FCGR2A,FCGR2B
Pathogenesis of Multiple Sclerosis	0.036307805	CCR5
Leucine Degradation I	0.036307805	MCCC2
Leukocyte Extravasation Signaling	0.047863009	NCF2,CYBB,NCF4
OX40 Signaling Pathway	0.051286138	CD3D,HLA-DPB1
Apoptosis Signaling	0.052480746	CASP8,MCL1
cAMP-mediated signaling	0.0616595	RGS18,PTGER3,S1PR1
Granzyme B Signaling	0.064565423	CASP8
Huntington's Disease Signaling	0.067608298	HSPA9,CASP1,CASP8
Rac Signaling	0.069183097	NCF2,CYBB
iCOS-iCOSL Signaling in T Helper Cells	0.072443596	PTPRC,CD3D
**Natural Killer Cell Signaling**	0.075857758	**FCGR2A,CD300A**
Type I Diabetes Mellitus Signaling	0.075857758	CASP8,CD3D
Phospholipase C Signaling	0.075857758	FCGR2A,FCGR2B,CD3D
CD28 Signaling in T Helper Cells	0.085113804	PTPRC,CD3D
Gαi Signaling	0.087096359	PTGER3,S1PR1
phagosome maturation	0.087096359	NCF2,CYBB
Protein Ubiquitination Pathway	0.087096359	CBL,HSPA9,BTRC
LXR/RXR Activation	0.089125094	TLR4,LYZ
G-Protein Coupled Receptor Signaling	0.089125094	RGS18,PTGER3,S1PR1
PI3K/AKT Signaling	0.089125094	JAK3,MCL1
Atherosclerosis Signaling	0.091201084	LYZ,CCR2
Tumoricidal Function of Hepatic Natural Killer Cells	0.09332543	CASP8

S. Table 3: Rank order of the significant compounds (p<=0.05) driven by 85 IF/TA genes

Ranking	Compound	RankScore	-Log(P)	PubChem	SideEffect (SIDER, OFFSIDES) (1-3)
1	methyldopate	-0.84391487	8	17276	Dizziness; drowsiness; dry mouth; headache; weakness
2	fluticasone	-0.81787979	8	444036	rheumatism:erythema:Pancreas:cholecystitis:ear pain:psoriasis:allergic contact dermatitis:contact dermatitis:acneiform eruptions:acne:cushingoid:growth suppression:ear infection:muscle stiffness:Decreased bone density:hypertension:pruritus:aches:Pain in extremity:nasal irritation:urticaria:tenderness:throat irritation:dry mouth:increased intraocular pressure:lump:anaphylactoid reactions:polyps:nasal burning:sleep disorder:plaque psoriasis:secondary infection:folliculitis:colitis:aphonia:face edema:cutaneous hypersensitivity:perennial allergic rhinitis:herpes simplex:facial telangiectasia:nausea:eczema:conjunctivitis:menopause:gastroenteritis viral:post nasal drip:postoperative complications:musculoskeletal pain:striae:glycosuria:Pneumocystis:gastrointestinal infection:sinus headache:hypercorticism:numbness:pneumonia:sinusitis:hypopigmentation:warts:blurred vision:oropharyngeal candidiasis:nasal dryness:cough:hemorrhage:hypersensitivity:chest congestion:caries:hyperglycemia:edema:Oral irritation:dizziness:Interactions:discomfort:Nasopharyngitis:eruptions:hoarseness:pharyngitis:fatigue:common cold:rhinorrhea:respiratory tract infection:secondary adrenocortical insufficiency:diarrhea:crusting:diabetes:bronchitis:oral moniliasis:spasm:oral lesions:moniliasis:paralysis:ulcer:anaphylaxis:viral infection:leukopenia:vaginal moniliasis:bacterial infections:abdominal discomfort:malaise:vasculitis:weight gain:rash:immunosuppression:agitation:stiffness:irritability:tongue disorder:pelvic inflammatory disease:osteoporosis:plaque:osteoporotic fracture:laryngitis:dry skin:nervousness:mouth ulceration:Stinging skin:inflammation:giddiness:nasal discharge:rigidity:skin infections:skin discoloration:skin irritation:flu:cyst:skin atrophy:hematoma:cataract:swelling:glaucoma:gastrointestinal moniliasis:lacrimation:dyspepsia:dysmenorrhea:sneezing:palpitations:stomach:numbness of fingers:stinging:blepharoconjunctivitis:perioral dermatitis:herpes:ecchymosis:nasal ulcer:hyperkinesia:migraine:cramps:vaginitis:fungal infection:infection:nasal septal perforation:atrophy:pustular psoriasis:angioedema:impetigo:blisters:hypertrichosis:Bone loss:nasal congestion:otitis media:lightheadedness:sore throat:epistaxis:thrombocytopenia:paradoxical bronchospasm:tightness of the throat:exacerbation of asthma:miliaria:gastroenteritis:throat infection:vulvovaginitis:wheezing:swollen throat:aggressive:otitis:abdominal pain:goiter:dyspnea:eye irritation:eczema herpeticum:irritant dermatitis:menstrual irregularities:insomnia:sepsis:protein allergy:vomiting:anxiety:telangiectasia:rash

3	cefaclor	-0.72444256	8	40958	lymphadenopathy:erythema:back pain:ear pain:renal impairment:decreased hemoglobin:confusion:elevated bilirubin:lymphopenia:pruritus:hemolytic anemia:urticaria:anemia:aplastic anemia:anaphylactoid reactions:paresthesia:decreased calcium:cough increased:face edema:pain:MCV:decreased platelet:nausea:syncope:conjunctivitis:abnormal liver function:nephropathy:elevated total bilirubin:leukorrhea:leukopenia:sinusitis:constipation:chills:interstitial nephritis:flatulence:hypersensitivity:somnolence:alkaline phosphatase increased:penicillin allergy:edema:dizziness:Urine analysis abnormal:nocturia:gamma glutamyl transpeptidase increased:asthenia:vertigo:diarrhea:decreased albumin:dysuria:seizures:anaphylaxis:tremor:lung disorder:neck pain:decreased sodium:malaise:rash:jaundice:agitation:respiratory disorder:heart failure:congestive heart failure:nervousness:moniliasis:sodium increased:SGPT increased:genital pruritus:sickness:agranulocytosis:cholestatic jaundice:creatine phosphokinase increased:nephritis:potassium increased:cholestasis:dysmenorrhea:peripheral edema:asthma:palpitations:pancytopenia:colitis:pseudomembranous colitis:cough:prolonged prothrombin time:anorexia:hypertonia:hyperkinesia:lactic dehydrogenase increased:vaginitis:lymphocytosis:superinfection:erythema multiforme:angioedema:rhinitis:toxic epidermal necrolysis:gastrointestinal symptoms:proteinuria:eruptions:vaginal moniliasis:thrombocytopenia:hepatitis:hemorrhage:gastritis:neutropenia:toxic nephropathy:dyspepsia:abdominal pain:chest pain:dyspnea:creatinine increased:hypotension:insomnia:epidermal necrolysis:vomiting:sweating:anxiety:maculopapular rash:myalgia:arthralgia:vasodilation:hallucinations:menstrual disorder:arthritis:eosinophilia:serum sickness:headache
4	methoxamine	-0.79674788	7.53648	6082	

5	letrozole	-0.72083589	5.72033	3902	erythema:back pain:fever:hypercalcemia:myocardial infarction:urinary tract infection:Musculoskeletal System Disorders:leg pain:portal vein thrombosis:hypertension:urinary frequency:elevated lipids:pruritus:bone pain:urticaria:elevated liver enzymes:dry mouth:effusion:chest pain:Chest wall pain:pain:memory impairment:Pain in extremity:hyperplasia:nausea:tumor:thrombophlebitis:hemorrhagic strokes:nephropathy:anorexia:musculoskeletal pain:pleural:vesicular rash:flushing:hypesthesia:lethargy:cerebrovascular accident:alopecia:Metabolic Disorders:menopause:blurred vision:cough:somnolence:endometrial cancer:lymphedema:edema:dizziness:Dependent edema:fatigue:weakness:endometrial hyperplasia:Endocrine Disorders:asthenia:vertigo:pleural effusion:diarrhea:myocardial ischemia:pulmonary embolism:epidermal necrolysis:respiratory disorder:hypercholesterolemia:anaphylaxis:arterial thrombosis:embolism:hemiparesis:Gastrointestinal Disorders:viral infection:leukopenia:malaise:weight loss:weight gain:rash:constipation:paresthesia:irritability:Bone loss:coronary heart disease:Connective Tissue Disorders:osteoporosis:heart failure:stomatitis:skin disorder:nervousness:vaginal irritation:vascular disease:thrombosis:Nervous System Disorders:sickness:flu:Mediastinal Disorders:abdominal pain:heart disease:Psychiatric Disorders:tachycardia:dyspepsia:Infestations:estrogen deficiency:ischemia:peripheral edema:palpitations:phlebitis:leg edema:thromboembolic events:Thoracic:Breast Disorders:BMD:increased appetite:decreased appetite:infection:erythema multiforme:angioedema:vein thrombosis:toxic epidermal necrolysis:hot flushes:cancer pain:hepatitis:hemorrhage:thromboembolism:effects of estrogen:sweating:cardiovascular disease:infarction:transient ischemic attack:vaginal discharge:dyspnea:vaginal hemorrhage:vaginal bleeding:angina:arthritis:insomnia:taste perversion:dysesthesia:thirst:vomiting:anxiety:maculopapular rash:breast pain:rash erythematous:arm pain:cataract:myalgia:arthralgia:eye irritation:increased sweating:night sweats:headache

6	articaine	-0.68016796	4.86377	32169	lymphadenopathy:back pain:ear pain:gingivitis:numbness:rhinitis:paresthesia:increased salivation:obstruction:hyperesthesia:otitis media:cough:hypersensitivity:skin disorder:nervousness:hemorrhage:somnolence:ulcer:mouth ulceration:pruritus:elevated blood pressure:sinus pain:edema:trismus:tingling:dizziness:constipation:otitis:abdominal pain:myalgia:facial paralysis:gum hemorrhage:pharyngitis:injection site pain:swelling:tachycardia:dyspepsia:asthenia:taste perversion:diarrhea:dysmenorrhea:burning sensation:vomiting:osteomyelitis:stomatitis:persistent cough:face edema:neuropathy:pain:dry mouth:paralysis:salivation:ecchymosis:tooth disorder:nausea:syncope:migraine:arthralgia:thirst:aspiration:neck pain:glossitis:drowsiness:infection:sinus congestion:headache:malaise:palpitations
7	dacarbazine	-0.67904164	4.8412	5353562	rash:Pancreas:extravasation:photosensitivity reaction:paresthesia:myelosuppression:fever:blurred vision:facial paresthesia:eruptions:stomatitis:hypersensitivity:thrombocytopenia:confusion:elevated liver enzymes:neutropenia:flu:urticaria:injection site pain:phototoxicity:fatigue:postural hypotension:arrhythmia:drug eruption:cancer:diarrhea:vomiting:eosinophilia:seizures:pain:vascular occlusion:hepatic necrosis:anaphylaxis:nausea:anorexia:alopecia:photosensitivity:myalgia:necrosis:leukopenia:hypotension:febrile neutropenia:fixed drug eruption:flushing:malaise:headache

8	carbimazole	-0.6625842	4.51838	31072	abdominal tenderness|Abnormal LFTs|abnormal movements|acute brain syndrome|adrenal neoplasm|aggression|agitated|alkalosis hypochloraemic|allergic dermatitis|alveolitis|anaemia of malignant disease|anaesthesia|anger|antipsychotic drug level increased|apathy|areflexia|arterial pressure NOS decreased|arteriovenous fistula thrombosis|ascites|asystole|Bacterial infection|bacterial toxaemia|basal cell carcinoma|basedow's disease|bile duct obstruction|biliary fibrosis|bipolar disorder|blood bilirubin increased|blood calcium|blood magnesium decreased|blood sodium increased|blunted affect|bone cancer metastatic|bone debridement|bone disorder|brain oedema|burning sensation|capillary leak syndrome|Carcinoma of Prostate|cardiac enlargement|cardiac output decreased|Cardiomyopathy|cardiovascular insufficiency|cerebral atherosclerosis|cerebral atrophy|cerebrovascular insufficiency|chronic active hepatitis|chronic left ventricular failure|circumstance or information capable of leading to medication error|clostridial infection|coagulation time prolonged|cognitive disorder|colonic obstruction|computerised tomogram|confabulation|consciousness fluctuating|c-reactive protein increased|csf lymphocyte count increased|death|depressed level of consciousness|depressed mood|dermatitis seborrheic|diabetes mellitus inadequate control|dizziness exertional|drug exposure during pregnancy|drug interaction|dyslogia|dysphemia|dystonia|ear haemorrhage|electrocardiogram qt corrected interval|electromechanical dissociation|elevated erythrocyte sedimentation rate|Embolism pulmonary|encephalopathy|Eosinophil Count Increased|excessive sleepiness|Extrasystoles Ventricular|facial spasm|filariasis|flu|gastric polyps|gastrointestinal bleed|general physical health deterioration|gingival disorder|goiter|grip strength|gynaecomastia|haemodialysis|hepatic enzyme abnormal|Hepatic failure|hepatitis|Hepatitis A|hepatitis acute|hepatitis B|hepatitis b positive|hepatotoxicity|hip arthroplasty|Hypercapnia|hyperparathyroidism primary|hyperpigmentation|hypersexuality|hypertonia|Hypomagnesaemia|hypoparathyroidism|hyporeflexia|illogical thinking|impaired self-care|inadequate diet|infected skin ulcer|Infection Upper Respiratory|insulinoma|intention tremor|kidney failure|lethargy|mallory-weiss syndrome|miosis|mononucleosis heterophile test positive|muscle swelling|muscle weakness|musculoskeletal chest pain|musculoskeletal stiffness|narcotic intoxication|negativism|Nephrogenic diabetes insipidus|neumonia|neutropenic sepsis|oral mucosal disorder|oropharyngeal pain|orthopnea|osteomyelitis|paranoid personality disorder|parathyroid tumour benign|Parkinson|peripheral embolism|peripheral sensory neuropathy|photosensitive

9	bethanechol	-0.6271433	3.86773	2370	eructation:seizures:bronchoconstriction:salivation:feeling of warmth:sweating:discomfort:colicky:tachycardia:lacrimation:borborygmi:asthma:miosis:diarrhea:urinary urgency:abdominal cramps:pain:nausea:cramps:colic:hypotension:sensations of heat:flushing:malaise:headache
10	neostigmine b	-0.62015572	3.7466	8246	
11	pipemidic acid	-0.61586913	3.67344	4831	
12	laudanosine	-0.60000219	3.41031	15548	

13	risperidone	-0.59066355	3.26106	5073	gastritis:influenza like illness:lymphadenopathy:ear pain:rectal hemorrhage:increased salivation:psoriasis:decreased hemoglobin:apnea:delusions:twitching:blood cholesterol increased:synostosis:lymphopenia:testosterone decreased:head titubation:aches:dry mouth:anemia:hypoproteinemia:anaphylactoid reactions:ejaculation disorder:delayed ejaculation:urinary retention:nuchal rigidity:extrasystoles:tumor:alopecia:conjunctivitis:paranoid reaction:bradycardia:anorexia:ocular hyperemia:papular rash:glycosuria:rigors:ECG abnormal:stridor:gingivitis:sarcoidosis:chills:somnolence:decreased sweating:nightmares:Musculoskeletal System Disorders:fatigue:stupor:Endocrine Disorders:eye infection:akinesia:vertigo:tinnitus:diabetes:bronchitis:ejaculation failure:female breast pain:enlargement:hypercholesterolemia:hemoptysis:embolism:tremor:ST segment depression:choking:menstrual disorder:eructation:jaundice:aphasia:paresthesia:dyskinesia:cheilitis:swelling of the lips:involuntary movements:chest congestion:nasal edema:eye rolling:vaginal discharge:posture abnormal:cerebral ischemia:hypersomnia:torticollis:gynecomastia:bronchopneumonia:glaucoma:tachycardia:creatine phosphokinase increased:lacrimation:Infestations:sinus tachycardia:ischemia:palpitations:obesity:balance disorder:dysphagia:delirium:incontinence:dystonia:QT prolonged:drowsiness:Connective Tissue Disorders:erythema multiforme:rhinitis:sluggishness:eruptions:crying abnormal:oligomenorrhea:muscle tightness:hepatitis:otitis:viral infection:lip:buttock pain:epistaxis:cholelithiasis:angina:artery occlusion:insomnia:respiratory disorder:liver fatty:rhabdomyolysis:deafness:warts:decreased serum iron:loss of consciousness:mood disorders:impotence:decreased visual acuity:Central Nervous System Disorders:diabetic coma:fever:geriatric:kidney pain:acne:esophagitis:leg pain:hypertension:hyperuricemia:photopsia:lichenoid dermatitis:diabetic ketoacidosis:urticaria:water intoxication:perineal pain:arrhythmia:death:ear disorder:agitation:generalized rash:reflux:delayed menstrual period:abnormal lacrimation:face edema:pain:Pain in extremity:metrorrhagia:decreased libido:subcutaneous abscess:nausea:eczema:changes in ECG:xerophthalmia:hypokalemia:leukorrhea:hypesthesia:localized infection:Disorders Nasal:pharyngolaryngeal pain:cough:skin ulcer:fatigability:coldness:rales:hemorrhoids:Immune System
14	alprostadil	-0.58104059	3.1116	5280723	

15	tubocurarine [illegible]	-0.57866766	3.07542	6000	abdominal cramps|addiction|adenitis|agitation|agranulocytosis|alkaline phosphatase increased|alopecia|anorexia|anxiety|arrest|arrhythmia|ataxia|black tongue|blurred vision|bradycardia|breast enlargement|bronchospasm|cerebrovascular accident|cholinergic crisis|choreoathetoid movements|coma|confusion|congestive heart failure|constipation|cramps|death|decreased libido|delusions|diarrhea|disorientation|distended bladder|distention|diuresis|dizziness|drowsiness|drug fever|dry mouth|edema|elevated liver function tests|enlargement|eosinophilia|epigastric distress|extrapyramidal symptoms|fatigue|fever|fibrillation|flushing|galactorrhea|grand mal|gynecomastia|hallucinations|headache|heart block|heart failure|hepatitis|hyperacusis|hyperkinesia|hypertension|hypomania|hypotension|ileus|im potence|incoordination|increased intraocular pressure|infarction|insomnia|intoxication|jaundice|lightheadedness|malaise|malignant syndrome|mental depression|muscarinic effects|mydriasis|myocardial infarction|myoclonus|nausea|neuroleptic malignant|neuropathy|nightmares|nocturia|numbness|palpitations|paralytic ileus|paresthesia|parotid swelling|peculiar taste|peripheral neuropathy|petechiae|pressure of speech|pruritus|psychosis|purpura|rash|salivation|seizures|shock|SIADH|stomatitis|sudd en death|sweating|swelling|tachycardia|Tachycardia Nodal|testicular swelling|thrombocytopenia|tingling|tinnitus|tonic - clonic seizures|tremor|urinary frequency|urinary retention|urticaria|ventricular extrasystoles|ventricular fibrillation|ventricular tachycardia|vomiting|weakness|weight gain
16	niclosamide	-0.75496217	3	4477	
17	tolnaftate	-0.73858675	3	5510	abdominal cramps|agitation|angina|anorexia|anxiety|confusion|cramps|dizziness|drowsiness|drug eruption|dysuria|elevated blood pressure|headache|insomnia|mydriasis|nausea|palpitations|rash|shock|spasm|sweating| tachycardia|tension|vertigo|vomiting|weakness
18	Prestwick-108	-0.70726167	3	93054	

19	doxycycline	-0.67299763	3	54671203	ear infection:back pain:aches:tenderness:dry mouth:BUN increased:proctitis:tooth sensitivity:gum hemorrhage:pain:distention:tooth disorder:low back pain:upset:post nasal drip:common cold:neck pain:leukopenia:sinus headache:gingivitis:photosensitivity reaction:sore throat:elevated blood pressure:Interactions:discomfort:gastric pain:tension:tension headache:rhinorrhea:sore gums:jaw pain:essential hypertension:moniliasis:inflammation:anxiety:vaginal moniliasis:fistula:Nasopharyngitis:Bone loss:aphthous stomatitis:stomatitis:skin infections:SGOT increased:dyspepsia:dysmenorrhea:stomach:tooth loss:cough:cramps:myalgia:fungal infection:premenstrual tension:sinus congestion:abdominal pain upper:periodontal disease:shoulder pain:nasal congestion:toothache:abdominal distention:abdominal pain:Periapical abscess:pharyngolaryngeal pain:insomnia:periodontal abscess:sore mouth:pulpitis:upset stomach:acid indigestion:arthralgia:flu symptoms:sinus infection:headache:esophagitis:hypertension:hemolytic anemia:urticaria:anemia:nausea:photosensitivity:sinusitis:purpura:anaphylactoid purpura:toxic nephropathy:hypersensitivity:edema:lupus:intracranial hypertension:diarrhea:bronchitis:ulcer:anaphylaxis:glossitis:rash:prolonged periods:sickness:pericarditis:swelling:exfoliative dermatitis:dermatitis:dysphagia:anorexia:esophageal ulcer:erythema multiforme:angioedema:toxic epidermal necrolysis:pseudotumor cerebri:thrombocytopenia:neutropenia:systemic lupus erythematosus:enterocolitis:epidermal necrolysis:vomiting:esophageal:abscess:Bulging fontanels:eosinophilia:serum sickness

20	syrosingopine	-0.56713105	2.90331	6769	abdominal pain|abnormal dreams|abnormal vision|aches|agranulocytosis|alopecia|anorexia|anxiety|arrest|arterial thrombosis|arthralgia|AV block|back pain|blurred vision|bradycardia|bronchospasm|catatonic|chest pain|colitis|confusion|congestive heart failure|conjunctivitis|constipation|cough|dermatitis|diarrhea|disorientation|dizziness|drowsiness|dry eyes|dyspepsia|dyspnea|dysuria|edema|elevated bilirubin|emotional lability|exfoliative dermatitis|eye itching|eye pain|fatigue|fever|flatulence|flushing|Gastrointestinal Disorders|hallucinations|headache|heartburn|heart failure|hot flushes|hyperkinesia|hypesthesia|hypotension|impotence|insomnia|ischemic colitis|laryngospasm|lightheadedness|loss of appetite|low cardiac output|lupus|malaise|Mediastinal Disorders|memory loss|mental depression|mesenteric arterial thrombosis|myalgia|nausea|Nervous System Disorders|nocturia|pain|palpitations|paresthesia|peripheral coldness|pharyngitis|pleuritic pain|pneumonia|postural hypotension|PR interval|pruritus|psychosis|purpura|rash|rash erythematous|Raynaud|respiratory distress|rhinitis|SGOT increased|shivering|sinus arrest|somnolence|sore throat|sweating|syncope|systemic lupus erythematosus|tenderness|Thoracic|thrombocytopenic purpura|thrombosis|tinnitus|urticaria|vascular disease|vertigo|visual disturbances|vivid dreams|vomiting|weakness|weight gain|wheezing
21	indoprofen	-0.56496588	2.8717	3718	
22	lisuride	-0.55861255	2.78025	28864	
23	ketotifen	-0.73276988	2.69897	3827	weight gain:rash:erythema multiforme:irritability:erythema:fever:eyelid swelling:epistaxis:thrombocytopenia:hepatitis:nervousness:hemorrhage:Stevens Johnson Syndrome:elevated liver enzymes:hypertension:sleep disturbances:tingling:dizziness:flu:urticaria:abdominal pain:dry mouth:eyelid disorder:insomnia:blood in stool:diarrhea:vomiting:stinging:pain:dry eyes:nausea:syncope:increased appetite:conjunctival hyperemia:sedation:infection:cystitis:headache
24	lanatoside C	-0.72786055	2.69897	3879	
25	decitabine	-0.69600787	2.69897	451668	

26	memantine	-0.67636757	2.69897	4054	extrapyramidal syndrome:lymphadenopathy:decreased visual acuity:back pain:diplopia:death:supraventricular tachycardia:myocardial infarction:involuntary muscle contractions:acne:cholestatic hepatitis:confusion:urinary tract infection:Stevens Johnson Syndrome:macular degeneration:urinary incontinence:hypertension:pruritus:abnormal renal function:eye pain:urticaria:blepharitis:renal failure:anemia:renal insufficiency:dyskinesia:neuralgia:gastroenteritis:hemiplegia:paresthesia:sleep disorder:reflux:hepatic failure:ileus:abnormal lacrimation:urinary retention:pain:Pain in extremity:ABNORMAL RENAL FUNCTION TEST:Hepatobiliary Disorders:organic brain syndromes:nausea:changes in ECG:syncope:corneal opacity:eczema:delirium:conjunctivitis:paranoid reaction:bradycardia:anorexia:atrial fibrillation:fever:tardive dyskinesia:deep vein thrombosis:leukopenia:cerebrovascular accident:alopecia:agranulocytosis:pneumonia:QT prolonged:apnea:necrosis:blurred vision:cellulitis:delusions:purpura:Endocrine Disorders:cough:conjunctival hemorrhage:thrombotic thrombocytopenic purpura:hypersensitivity:skin ulcer:somnolence:alkaline phosphatase increased:hyperglycemia:increased appetite:edema:hyponatremia:nightmares:dizziness:ataxia:detachment:asthenia:Nasopharyngitis:decreased appetite:fatigue:aspiration pneumonia:stupor:respiratory tract infection:asthma:vertigo:diarrhea:tinnitus:diabetes:bronchitis:pulmonary embolism:encephalopathy:gastrointestinal hemorrhage:pulmonary edema:dysuria:hyperlipidemia:intracranial hemorrhage:cerebral hemorrhage:hemoptysis:anxiety:embolism:tremor:Gastrointestinal Disorders:neurosis:hypesthesia:retinal detachment:Urinary System Disorders:vascular disease:malaise:weight loss:Psychiatric Disorders:rash:incoordination:constipation:aphasia:agitation:emotional lability:apathy:hematuria:Connective Tissue Disorders:lethargy:heart failure:skin disorder:nervousness:spasticity:ulcer:abnormal gait:diverticulitis:libido increased:SGPT increased:thrombosis:Nervous System Disorders:myopia:acute renal failure:retinal hemorrhage:flu:Mediastinal Disorders:arrest:hypothermia:abdominal pain:grand mal:cataract:glaucoma:dermatitis:lacrimation:creatinine increased:convulsions:disorientation:peripheral edema:torsade de pointes:pancytopenia:dysphagia:suicidal ideation:colitis:Thoracic:myoclonus:thrombophlebitis:incontinence:hypertonia:hyperkinesia:esophageal ulcer:hypoglycemia:irritability:heart disease:aggressive reaction:macula:xerophthalmia:thrombocytopenic purpura:infection:hallucinations:AV

27	etodolac	-0.6723495	2.52288	3308	gastritis:erythema:lymphadenopathy:renal impairment:rectal hemorrhage:fever:myocardial infarction:hematemesis:esophagitis:dizziness:confusion:elevated liver enzymes:epigastric pain:hypertension:abdominal distention:urinary frequency:pruritus:hemolytic anemia:urticaria:renal failure:dry mouth:anemia:jaundice:renal insufficiency:arrhythmia:anaphylactoid reactions:death:bronchitis:hepatic failure:colitis:nausea:syncope:pulmonary infiltrates:photosensitivity:meningitis:thirst:duodenitis:flushing:leukorrhea:leukopenia:cerebrovascular accident:alopecia:conjunctivitis:pneumonia:sinusitis:hyperpigmentation:chills and fever:necrosis:blurred vision:chills:purpura:interstitial nephritis:flatulence:hypersensitivity:somnolence:hyperglycemia:edema:cholestatic hepatitis:discomfort:BUN increased:gastric pain:pharyngitis:fatigue:taste perversion:asthenia:vertigo:diarrhea:abnormal renal function:burning sensation:tinnitus:kidney calculus:gastrointestinal hemorrhage:dysuria:desquamation:anaphylaxis:leukocytoclastic vasculitis:tremor:peptic ulcer:bleeding time increased:glossitis:shock:cystitis:abdominal discomfort:malaise:heartburn:vasculitis:rash:eructation:constipation:photophobia:paresthesia:bronchospasm:deafness:hematuria:ulcerative stomatitis:heart failure:congestive heart failure:stomatitis:nervousness:ulcer:peeling:agranulocytosis:papillary necrosis:cutaneous vasculitis:oliguria:tachycardia:cholestatic jaundice:dermatitis:dyspepsia:convulsions:vesiculobullous rash:nephritis:asthma:palpitations:gastric ulcer:pancytopenia:ecchymosis:anorexia:intestinal ulcer:visual disturbances:polyuria:aplastic anemia:infection:irritability:erythema multiforme:angioedema:rhinitis:toxic epidermal necrolysis:coma:exfoliative dermatitis:pancreatitis:proteinuria:cardiospasm:thrombocytopenia:hepatitis:hemorrhage:neutropenia:sweating:respiratory depression:infarction:abdominal pain:taste loss:dyspnea:hypotension:insomnia:sepsis:hearing loss:epidermal necrolysis:vomiting:anxiety:maculopapular rash:epidermal necrosis:abnormal stools:hepatic necrosis:myalgia:arthralgia:hallucinations:eosinophilia:melena:uterine hemorrhage:headache
28	myosmine	-0.65081977	2.52288	442649	
29	Gly-His-Lys	-0.63532509	2.52288	16219366	

30	5149715	-0.61267108	2.52288	5149715	agranulocytosis|amenorrhea|anaphylaxis|ataxia|breast carcinoma|carcinoma|confusion|cramps|diarrhea|drowsiness|drug eruption|eosinophilia|epidermal necrolysis|eruptions|fever|gastric hemorrhage|gastritis|gynecomastia|headache|hemorrhage|hyperkalemia|hypersensitivity|lethargy|menstrual irregularities|nausea|postmenopausal bleeding|rash|renal failure|toxic epidermal necrolysis|ulcer|urticaria|vasculitis|vomiting
31	chlortetracycli	-0.53580177	2.46745	54682468	abdominal pain|abnormal liver function|acute interstitial nephritis|agitation|agranulocytosis|allergic contact dermatitis|altered mental status|anemia|angina|angioedema|anorexia|aphasia|aplastic anemia|apprehension|arrest|arthralgia|asthenia|asthma|ataxia|atrial fibrillation|AV block|blackwater fever|blindness|blurred vision|bradycardia|bronchospasm|Bullous dermatitis|cerebral ischemia|chest pain|chills|Clotting Disorders|coma|confusion|contact dermatitis|convulsions|cutaneous vasculitis|deafness|delirium|dermatitis|diarrhea|diplopia|disorientation|disseminated intravascular coagulation|dizziness|drug eruption|dyspnea|dystonic reactions|ecchymosis|ECG abnormal|edema|epidermal necrolysis|eruptions|erythema|erythema multiforme|esophagitis|exfoliative dermatitis|fatigue|fever|fibrillation|fixed drug eruption|flushing|gastric irritation|gastrointestinal distress|granulomatous hepatitis|headache|hearing loss|heartburn|hemoglobinuria|hemolytic anemia|hemolytic uremic syndrome|hemorrhage|hepatitis|hypersensitivity|hypocalcemia|hypoglycemia|hypokalemia|hypomagnesemia|hypoprothrombinemia|hypotension|Idiopathic thrombocytopenic purpura|ileus|Interactions|interstitial nephritis|ischemia|jaundice|leukopenia|lightheadedness|liver function tests abnormal|lupus|lymphadenopathy|muscle weakness|myalgia|mydriasis|nausea|necrosis|nephritis|nervousness|neuritis|neutropenia|night blindness|nodules|optic neuritis|pain|palpitations|pancytopenia|petechiae|photophobia|photosensitivity|photosensitivity reaction|pigmentation|pneumonia|polymorphic ventricular tachycardia|postural hypotension|pruritus|pulmonary edema|purpura|rash|renal failure|renal impairment|seizures|shock|sicca|sudden loss of vision|suicide|sweating|syncope|systemic lupus erythematosus|systemic vascular resistance|tachycardia|thrombocytopenia|thrombocytopenic purpura|thrombotic thrombocytopenic purpura|tinnitus|torsade de pointes|toxic epidermal necrolysis|tremor|uremic syndrome|urticaria|uveitis|U wave|vascular resistance|vasculitis|vasodilation|ventricular extrasystoles|ventricular fibrillation|ventricular tachycardia|vertigo|visual disturbances|visual field defect|vomiting|weakness|withdrawn

32	digoxigenin	-0.69317006	2.39794	15478	
33	gibberellic aci	-0.62956142	2.39794	6466	
34	helveticoside	-0.62869244	2.39794	10503	
35	viomycin	-0.5300608	2.39255	3037981	
36	pheneticillin	-0.53000612	2.39184	272833	
37	**esculetin**	**-0.52049252**	**2.27109**	5281416	

38	trichlormethia	-0.517376	2.23244	5560	abdominal mass|Abnormal Gait|accelerated hypertension|actinomycosis|acute kidney failure|acute pancreatitis|acute promyelocytic leukaemia|adams-stokes syndrome|adenitis|adenocarcinoma|Adenopathy|ADH inappropriate|adrenal insufficiency|adynamic ileus|agranulocytoses|agraphia|alanine aminotransferase increased|alcohol interaction|allergic transfusion reaction|altered state of consciousness|alveolar soft part sarcoma|amentia|anaemia|angina|angioplasty|Anorexia|antibody test|anti-insulin antibody|anti-insulin antibody positive|aortic aneurysm|apathy|aphthous stomatitis|arteriosclerosis obliterans|Arthritis rheumatoid|ascites|Aspartate Aminotransferase Increase|atrioventricular block|atrophy|autoimmune thyroiditis|azotaemia|bacterial toxaemia|behcet's syndrome|bence jones protein urine|bicytopenia|black stools|blood albumin abnormal|blood albumin decreased|blood alkaline phosphatase increased|blood bilirubin increased|blood cholinesterase|blood corticotrophin|blood cortisol decreased|blood creatine phosphokinase increased|blood creatinine|blood creatinine increased|blood glucose decreased|blood immunoglobulin e|blood immunoglobulin g increased|blood lactate dehydrogenase increased|blood potassium decreased|blood potassium increased|blood pressure diastolic decreased|blood pressure inadequately controlled|blood pressure systolic increased|blood sodium|blood sodium increased|blood triglycerides increased|blood urea increased|blood uric acid|body temperature increased|bone cancer metastatic|bone fracture|bone inflammation|bone marrow failure|bradycardia|brain natriuretic peptide increased|brain oedema|breast enlargement|bronchoalveolar lavage|bronchostenosis|cardiac failure|cardiac failure acute|cardiac failure chronic|cardiac pacemaker insertion|cardiogenic shock|cardio-respiratory arrest|cartilage development disorder|cataract|cd4 lymphocytes decreased|cd8 lymphocytes decreased|cell marker increased|cerebral artery embolism|cerebral artery stenosis|cerebral infarct|chemotherapeutic drug level increased|cholangiolitis|chromaturia|Chronic Kidney Disease|colitis collagenous|color blindness|condition aggravated|constipated|contact dermatitis|coughing blood|c-reactive protein increased|csf monocyte count decreased|csf monocyte count increased|cubital tunnel syndrome|decreased activity|decreased interest|dehydration|dental abscess|depressed level of consciousness|dermatitis bullous|dermatitis exfoliative|dermatitis medicamentosa|detachment of retinal pigment epithelium|device interaction|Diabetes|diabetic gangrene|Diabetic Nephropathy|dialysis|disease of liver|Disorder Lung|disorder Renal|disseminated intravascular coagulation|disseminated

<table>
<tr><td>39</td><td>anisomycin</td><td>-0.65584313</td><td>2.22185</td><td>253602</td><td>abdominal discomfort|abdominal distress|ache|acute hepatitis|acute renal failure|aggressive|agranulocytosis|allergic pneumonitis|alopecia|alveolitis|anaphylactoid reactions|anaphylaxis|anemia|anorexia|aphasia|aplastic anemia|arterial thrombosis|arthralgia|aseptic meningitis|ataxia|azotemia|back pain|bilirubinemia|blindness|blurred vision|bone disorder|cerebral thrombosis|cerebrovascular accident|chest pain|chills|chills and fever|cirrhosis|CMV infection|cognitive dysfunction|coma|confusion|conjunctivitis|convulsions|cord|cough|cryptococcosis|cyanosis|cystitis|cytomegaloviral pneumonia|death|decreased hematocrit|decreased serum albumin|deep vein thrombosis|dementia|dermatitis|diabetes|diarrhea|discomfort|dizziness|drowsiness|dysarthria|dyspnea|dysuria|ecchymosis|edema|effusion|elevated liver enzymes|elevated liver function tests|elevated uric acid|embolism|embolus|encephalopathy|enteritis|eosinophilia|epidermal necrolysis|epistaxis|erythema|erythema multiforme|exfoliative dermatitis|extravasation|Eye Disorders|fatigue|febrile neutropenia|fetal death|fever|fibrosis|folliculitis|furunculosis|gastrointestinal reactions|Gastrointestinal Toxicity|gingivitis|glossitis|gynecomastia|headache|hematemesis|hematuria|hemiparesis|hemorrhage|hepatic failure|hepatitis|Hepatobiliary Disorders|herpes|histoplasmosis|hypersensitivity|hyperuricemia|Hypogammaglobulinemia|hypotension|impotence|infection|infertility|inflammation|interstitial pneumonitis|irritability|lethargy|leukoencephalopathy|leukopenia|loss of libido|lung disorder|lymphadenopathy|lymphoproliferative disorders|lysis|malaise|melena|meningitis|menstrual disorder|mood disturbances|myalgia|nausea|neck rigidity|necrosis|nephropathy|neutropenia|nocardiosis|obstruction|obstructive pulmonary disease|oligospermia|opportunistic infections|osteonecrosis|osteoporosis|pain|Pancreas|pancreatitis|pancytopenia|paresis|pericardial effusion|pericarditis|petechiae|pharyngitis|phlebitis|photosensitivity|plaque|pleural|pleural effusion|Pneumocystis|pneumonia|polyuria|productive cough|proteinuria|pruritus|pulmonary edema|pulmonary embolism|pulmonary fibrosis|pulmonary infections|Pulmonary lymphoma|pulmonary toxicity|radiculopathy|rales|rash|renal failure|respiratory failure|respiratory tract infection|retinal vein thrombosis|rigidity|secondary malignancy|seizures|sepsis|skin</td></tr>
<tr><td>40</td><td>chloropyrazine</td><td>-0.63107833</td><td>2.1549</td><td>73277</td><td></td></tr>
</table>

41	topiramate	-0.62603372	2.1549	5284627	gastritis:lymphadenopathy:ear pain:increased salivation:delusions:lymphopenia:aches:dry mouth:anemia:expressive language disorder:ejaculation disorder:urinary retention:visual field defect:tumor:alopecia:conjunctivitis:paranoid reaction:bradycardia:anorexia:rigors:abnormal electroencephalogram:incoordination:gingivitis:body odor:somnolence:myalgia:color blindness:Musculoskeletal System Disorders:fatigue:stupor:Endocrine Disorders:tubular:vertigo:parosmia:diabetes:bronchitis:thinking abnormal:testis disorder:embolism:tremor:Urinary System Disorders:menstrual disorder:eructation:paresthesia:dyskinesia:nephrolithiasis:gastrointestinal pain:glaucoma:tachycardia:angle closure glaucoma:lacrimation:abnormal hair texture:Abnormal thyroid:palpitations:obesity:pancytopenia:dysphagia:delirium:incontinence:dystonia:Reaction Emotional:manic:lymphocytosis:heat intolerance:Connective Tissue Disorders:rhinitis:hypocholesteremia:blisters:hiccup:aggressive:otitis:taste loss:alcohol intolerance:angina:prostate:hepatitis:epidermal necrolysis:respiratory disorder:neuropathy:bipolar disorder:vasodilation:impotence:mydriasis:fever:hyperchloremia:kidney pain:acne:esophagitis:leg pain:hypertension:pruritus:urticaria:arrhythmia:death:gastroenteritis:reflux:bronchospasm:Autonomic Nervous System Disorders:face edema:pain:memory impairment:tooth disorder:decreased libido:nausea:eczema:convulsions aggravated:xerophthalmia:hypokalemia:Oligohidrosis:kidney calculus:leukorrhea:hypesthesia:SGOT increased:aggravated depression:cough:alkaline phosphatase increased:hemorrhoids:insomnia:dizziness:ataxia:weakness:respiratory tract infection:decreased sweating:diarrhea:AV block:impaired concentration:encephalopathy:strabismus:diabetic peripheral neuropathy:apraxia:Eye Disorders:Ejaculation Premature:Eye Abnormality:arthrosis:peripheral neuropathy:drug withdrawal:weight loss:weight gain:tongue edema:constipation:photophobia:agitation:folliculitis:emotional lability:apathy:Nervous System Disorders:hyperlipidemia:myopia:grand mal:oliguria:dyspepsia:dysmenorrhea:peripheral edema:phlebitis:mental depression:abdomen enlarged:carcinoma:thrombocythemia:increased appetite:polyuria:upper motor neuron
42	etynodiol	-0.62292423	2.1549	9270	

43	buspirone	-0.50617838	2.09725	2477	loss of interest:erythema:flu:rectal hemorrhage:increased salivation:weight gain:alcohol abuse:myocardial infarction:acne:confusion:muscle weakness:hypertension:pruritus:chest pain:hostility:tenderness:enuresis:photophobia:altered taste:irritable bowel:delayed ejaculation:emotional lability:urinary retention:aphonia:face edema:pain:metrorrhagia:nausea:changes in ECG:syncope:alopecia:conjunctivitis:extrapyramidal symptoms:bradycardia:fever:musculoskeletal pain:infarction:leukopenia:incoordination:slurred:nocturia:numbness:restless legs:gastrointestinal distress:blurred vision:chills:sore throat:dissociative reaction:flatulence:neck rigidity:salivation:edema:nightmares:ataxia:akathisia:sore eyes:dystonic reactions:fatigue:weakness:stupor:vertigo:tinnitus:dysuria:seizures:spasm:thinning of nails:tremor:malaise:anger:weight loss:libido increased:rash:cerebrovascular accident:constipation:cardiomyopathy:dyskinesia:angioedema:pelvic inflammatory disease:hyperventilation:involuntary movements:heart failure:congestive heart failure:chest congestion:dry skin:nervousness:eye pain:excitement:rigidity:hesitancy:cogwheel rigidity:tunnel vision:inner ear:vivid dreams:tachycardia:dysphoria:burning tongue:palpitations:suicidal ideation:ecchymosis:anorexia:dystonia:cramps:increased appetite:redness:eosinophilia:drowsiness:galactorrhea:urticaria:eye itching:blisters:nasal congestion:amenorrhea:epistaxis:thrombocytopenia:hiccup:hemorrhage:claustrophobia:easy bruising:euphoria:hypersensitivity:cold intolerance:dyspnea:menstrual irregularities:depersonalization:vomiting:psychosis:thinking abnormal:arthralgia:hallucinations:impotence:hypotension:headache
44	pinacidil	-0.61945716	2.09691	4826	
45	molindone	-0.50566442	2.09118	23897	weight loss:libido increased:rash:urinary retention:galactorrhea:constipation:agitation:dyskinesia:weight gain:blurred vision:involuntary movements:amenorrhea:tightness of the throat:salivation:surgical intervention:rigidity:euphoria:lens opacities:protrusion of the tongue:akathisia:dyspnea:dry mouth:gynecomastia:tachycardia:akinesia:hypotension:spasm:dysphagia:adhere:nausea:tardive dyskinesia:hyperkinesia:motor restlessness:tremor:priapism:extrapyramidal symptoms:leukocytosis:dystonia:drowsiness:leukopenia

46	protriptyline	-0.50418817	2.07382	4976	fever:myocardial infarction:delusions:adenitis:confusion:hyperpyrexia:hypertension:urinary frequency:pruritus:urticaria:dry mouth:increased intraocular pressure:paralytic ileus:jaundice:arrhythmia:paresthesia:urinary retention:decreased libido:nausea:alopecia:extrapyramidal symptoms:flushing:leukopenia:cerebrovascular accident:nocturia:numbness:blurred vision:purpura:addiction:edema:nightmares:dizziness:ataxia:fatigue:weakness:diarrhea:tinnitus:seizures:enlargement:disorientation:tremor:black tongue:peripheral neuropathy:malaise:weight gain:rash:incoordination:constipation:agitation:marrow depression:stomatitis:tingling:heart block:agranulocytosis:breast enlargement:peculiar taste:gynecomastia:swelling:tachycardia:sweating:petechiae:hypomania:palpitations:panic:ileus:anorexia:cramps:drowsiness:abdominal cramps:drug fever:testicular swelling:galactorrhea:thrombocytopenia:infarction:postural hypotension:hypotension:insomnia:vomiting:anxiety:psychosis:parotid swelling:SIADH:neuropathy:hallucinations:impotence:mydriasis:eosinophilia:epigastric distress:headache
47	cefalonium	-0.50223078	2.05095	21743	
48	cefotetan	-0.50219797	2.05057	53025	superinfection:erythema:rash:erythema multiforme:renal impairment:positive direct Coombs test:fever:vaginal moniliasis:toxic nephropathy:hypersensitivity:thrombocytopenia:hemorrhage:moniliasis:neutropenia:pruritus:hemolytic anemia:agranulocytosis:urticaria:discomfort:anemia:abnormal liver function:aplastic anemia:cholestasis:diarrhea:epidermal necrolysis:vomiting:elevated bilirubin:seizures:phlebitis:pain:pancytopenia:anaphylaxis:colitis:pseudomembranous colitis:nausea:prolonged prothrombin time:abdominal pain:thrombocythemia:nephropathy:vaginitis:eosinophilia:leukopenia

49	gentamicin	-0.67324137	2.04576	3467	weight loss:erythema:rash:weakness:renal impairment:numbness:vein thrombosis:thrombosis:increased salivation:confusion:fever:pain at the injection:lethargy:myasthenia:purpura:hypocalcemia:stomatitis:hypersensitivity:pseudotumor:skin disorder:hyperemia:twitching:muscle weakness:necrosis:hypertension:decreased appetite:dehydration:tingling:dizziness:pruritus:respiratory depression:agranulocytosis:pseudotumor cerebri:urticaria:tetany:fat necrosis:anemia:myasthenia gravis:visual disturbances:hypomagnesemia:organic brain syndromes:hypervolemia:oliguria:anaphylactoid reactions:hypotension:convulsions:vertigo:hearing loss:tinnitus:decreased serum calcium:vomiting:atrophy:skin tingling:encephalopathy:phlebitis:laryngeal edema:fibrosis:thrombocytopenia:pain:pulmonary fibrosis:myasthenia gravislike syndrome:salivation:hepatomegaly:splenomegaly:nausea:neuropathy:edema:alopecia:sensitization:arthralgia:conjunctival hyperemia:hallucinations:injection site pain:eosinophilia:hypokalemia:thrombocytopenic purpura:peripheral neuropathy:extravasation:headache:leukopenia:roaring in the ears
50	sulfamonome	-0.63053021	2.04576	5332	
51	Prestwick-692	-0.62555098	2.04576		
52	mevalolacton	-0.62244932	2.04576	10428	
53	Prestwick-967	-0.60985647	2.04576	110737	
54	arcaine	-0.50143251	2.04168	26968	

55	atracurium be	-0.49977036	2.02246	47319	abdominal compartment syndrome|abdominal sepsis|abnormal EEG|abnormal movements|accelerated hypertension|accidental exposure|accidental overdose|Acidosis|activated partial thromboplastin time prolonged|activated partial thromboplastin time shortened|acute coronary syndrome|acute generalised exanthematous pustulosis|acute kidney failure|acute pancreatitis|acute pulmonary oedema|Acute Respiratory Distress Syndrome|adnexitis|adrenal mass|agitated|air embolism|airway complication of anaesthesia|akinesia|albuminuria|alkalosis|alkalosis hypochloraemic|allergic dermatitis|allergy test positive|alveolitis|amniotic fluid embolus|anaemia|anaesthetic complication|anaesthetic complication neurological|anaesthetic complication pulmonary|anal dilatation|anal fissure|anaphylactic reaction|angiitis|angioedema|anticholinergic syndrome|anuria|apallic syndrome|apgar score low|Apnea|areflexia|Arrhythmia|arterial pressure NOS decreased|arterial thrombosis|arteriosclerosis coronary artery|arteriovenous fistula thrombosis|ascites|ascites infection|aspiration bone marrow|asthmatic crisis|asystole|atonic urinary bladder|atrial tachycardia|autonomic instability|Bacteraemia|bacteroides infection|bile duct obstruction|biliary tract disorder|birth trauma|bleeding Vaginal|Blepharospasm|blood albumin decreased|blood chloride increased|blood creatine phosphokinase increased|blood creatinine increased|blood fibrinogen increased|blood glucose|blood glucose fluctuation|blood in urine|blood methaemoglobin present|blood ph decreased|blood phosphorus decreased|blood potassium decreased|blood potassium increased|blood pressure diastolic|blood pressure immeasurable|blood pressure systolic decreased|blood pressure systolic increased|bradycardia|bradycardia neonatal|brain contusion|brain death|brain herniation|brain neoplasm|brain oedema|brain stem haemorrhage|bronchiolitis|bulbar palsy|bundle branch block left|butterfly rash|Caesarean Section|carcinoid syndrome|cardiac disease|Cardiac ischemia|cardiac murmur|cardiogenic shock|Cardiomyopathy|cardio-respiratory arrest|cardiovascular disorder|catheter site haemorrhage|central venous pressure decreased|cerebral artery embolism|cerebral hyperperfusion syndrome|cerebral perfusion pressure decreased|cervical laser therapy|chest x-ray abnormal|chronic sinusitis|circumstance or information capable of leading to medication error|clonus|clostridial infection|colon polypectomy|coma|complications of transplanted liver|confabulation|congenital pyelocaliectasis|congenital tricuspid valve incompetence|conversion disorder|crackles lung|crystal urine|cyanosis|cyanosis neonatal|cytokine release syndrome|cytolytic hepatitis|Deafness neurosensory|delayed recovery from anaesthesia|depressed level of

56	nifuroxazide	-0.49788951	2.00086	5337997	abnormal behaviour|Abnormal Laboratory Findings|accident|Acidosis|acute generalised exanthematous pustulosis|acute vestibular syndrome|adenoma|agranulocytoses|anaemia|attempted suicide|aura|birth trauma|bladder retention|blood glucose|blood ketone body increased|blood ketone body present|blood sodium|blurred vision|Breast cancer|bundle branch block right|cardiac stress test abnormal|cardiogenic shock|chest pain|choking sensation|colitis collagenous|confabulation|coronary artery stenosis|dehydration|dermographism|diarrhea|dizziness|drug exposure during pregnancy|Dyspnea exertional|ecg signs of ventricular hypertrophy|elevated triglycerides|emesis|emphysema|eosinophilic cellulitis|erythema|esophageal ulcer|exercise electrocardiogram abnormal|gastrointestinal disorder|general physical health deterioration|henoch-schonlein purpura|leukopenia neonatal|lung edema|macrocytosis|metabolic disorder|motor retardation|nodule|obstructive airways disorder|pancreatic carcinoma metastatic|platelet count|productive cough|pseudomonal sepsis|septic shock|speech disorder developmental|spinning sensation|staphylococcal bacteraemia|superinfection lung|throat tightness
57	palmatine	-0.64549781	2	19009	

58	ramipril	-0.60595471	2	5362129	erythema:depressed mood:agitation:increased salivation:psoriasis:fever:conjunctivitis:vascular stenoses:confusion:increase in urinary output:constipation:hemolytic anemia:urticaria:renal failure:dry mouth:anemia:renal insufficiency:arrhythmia:anaphylactoid reactions:gastroenteritis:chest pain:artery stenosis:bronchospasm:hepatic failure:cough increased:pain:decreased libido:nausea:syncope:alopecia:photosensitivity:nephropathy:hyponatremia:renal artery stenosis:flushing:leukopenia:hypertensive:Raynaud:pneumonia:sinusitis:cerebrovascular disorder:purpura:cough:hypersensitivity:somnolence:salivation:edema:hypoglycemia:dizziness:polyneuritis:increased sweating:abnormal liver function:fatigue:hearing loss:respiratory tract infection:asthenia:vertigo:diarrhea:tinnitus:abnormal renal function:leukocytosis:infection:bronchitis:eosinophilic pneumonitis:pemphigoid:tremor:ischemic stroke:abdominal discomfort:malaise:vasculitis:weight gain:rash:jaundice:paresthesia:marrow depression:nervousness:pemphigus:pruritus:acute renal failure:acute liver failure:flu:ESS:enanthema:liver damage:tachycardia:cholestatic jaundice:liver function tests abnormal:dyspepsia:convulsions:nasal congestion:exacerbation of psoriasis:peripheral edema:palpitations:pancytopenia:dysphagia:anorexia:cramps:necrosis:elevated erythrocyte sedimentation rate:positive ANA:discomfort:erythema multiforme:amnesia:toxic epidermal necrolysis:pancreatitis:proteinuria:neuralgia:lightheadedness:epistaxis:thrombocytopenia:hepatitis:maculopapular rash:neutropenia:sleep disturbances:sweating:abdominal pain:taste loss:dyspnea:abnormal vision:postural hypotension:creatinine increased:angina:insomnia:taste perversion:epidermal necrolysis:vomiting:anxiety:onycholysis:hepatic necrosis:neuropathy:Lower Respiratory Tract Infections:myalgia:arthralgia:upper respiratory tract infection:impotence:arthritis:eosinophilia:headache

59	progesterone	-0.4970475	1.99125	5994	gastritis:erythema:back pain:ear pain:Immune System Disorders:rectal disorder:hypotension:Autonomic Nervous System Disorders:fever:feeling drunk:breast tenderness:acne:Genetic Disorders:cleft lip:Nervous System Disorders:urinary tract infection:aggressive:leg pain:Ovarian Hyperstimulation:Psychiatric Disorders:gallbladder disease:urinary frequency:septal defect:pruritus:genital pruritus:bone pain:urticaria:tenderness:transient ischemic attack:dry mouth:anemia:perineal pain:dysphagia:anaphylactoid reactions:death:gastroenteritis:vaginal discharge:sleep disorder:generalized rash:bronchitis:cleft palate:hand swelling:forgetful:hemangiomas:face edema:pain:dyspareunia:distention:decreased libido:Hepatobiliary Disorders:lymphadenopathy:metrorrhagia:nausea:syncope:melasma:tumor:alopecia:conjunctivitis:menorrhagia:xerophthalmia:vaginal dryness:tension:cerebral thrombosis:flushing:Perineal Pain Female:leukorrhea:ovarian cyst:SGOT increased:uterine spasm:vaginal bleeding:herpes:pneumonia:sinusitis:seborrhea:constipation:cardiovascular disease:speech disorder:blurred vision:feeling abnormal:purpura:ovarian cancer:cough:flatulence:hypersensitivity:drunk:somnolence:increased appetite:elevated blood pressure:night sweats:edema:optic neuritis:congenital heart disease:dizziness:Musculoskeletal System Disorders:discomfort:Nasopharyngitis:vaginitis:herpes simplex:pharyngitis:fatigue:tooth disorder:nocturia:stupor:vaginal irritation:respiratory tract infection:bloating:hiatal hernia:asthenia:vertigo:diarrhea:tinnitus:dysuria:porphyria:pulmonary embolism:confusion:chorea:enlargement:moniliasis:hirsutism:cervical erosion:myopathy:disorientation:anaphylaxis:impaired concentration:Eye Disorders:elevated liver enzymes:embolism:tremor:Gastrointestinal Disorders:viral infection:neuritis:shock:choking:cystitis:worry:menstrual disorder:spasm:weight loss:hypertension:rash:eructation:jaundice:SGPT increased:paresthesia:emotional lability:irritability:swollen tongue:Decreased carbohydrate tolerance:breast carcinoma:respiratory disorder:hyperventilation:hypocalcemia:acute pancreatitis:skin disorder:nervousness:slurred:blood glucose increased:abnormal gait:vascular disease:hernia:thrombosis:skin discoloration:flu:breast enlargement:breast discharge:heart disease:swelling:tachycardia:cholestatic jaundice:sweating:convulsions:cholestasis:glucose increased:dysmenorrhea:peripheral edema:ventricular septal defect:asthma:palpitations:difficulty in walking:mental depression:influenzalike symptoms:diplopia:musculoskeletal pain:Mediastinal
60	N6-methylade	-0.62662061	1.95861	102175	
61	succinylsulfat	-0.61217988	1.95861	5315	

62	folic acid	-0.59058994	1.95861	6037	abdominal pain|abnormal renal function|abnormal vision|acidosis|acute renal failure|agitation|amnesia|anaphylactoid reactions|anaphylaxis|angina|anosognosia|anoxia|anxiety|apnea|arrest|arrhythmia|asthma|atrial fibrillation|AV block|back pain|blindness|bradycardia|bronchitis|bundle branch block|BUN increased|cardiovascular disease|cerebrovascular disorder|chest pain|coma|confusion|conjunctivitis|convulsions|cortical blindness|cramps|diarrhea|diplopia|discomfort|disseminated intravascular coagulation|dizziness|drowsiness|dry mouth|dry skin|dyskinesia|dyspepsia|dyspnea|ear pain|edema|embolism|epilepsy|erythema|esophagitis|extrasystoles|face edema|fatigue|feeling of warmth|fever|fibrillation|flushing|headache|hearing loss|heart failure|hematuria|hypersensitivity|hypertension|hypesthesia|hypoglycemia|hypotension|increased salivation|increased sweating|inflammation|injection site inflammation|injection site pain|injection site reaction|insomnia|ischemia|leg cramps|malaise|migraine|mL i|myocardial infarction|nausea|nervousness|pain|paresthesia|parosmia|periorbital edema|peripheral edema|peripheral ischemia|pharyngeal edema|pharyngitis|polymyalgia|polymyalgia rheumatica|precordial chest pain|pruritus|pulmonary edema|pulmonary embolism|rash|rash erythematous|reactions to contrast media|renal failure|respiratory arrest|respiratory depression|respiratory disorder|respiratory tract infection|rhinitis|rigors|sensory disturbance|shock|sinus bradycardia|sinusitis|somnolence|stupor|sweating|syncope|taste perversion|tinnitus|upper respiratory tract infection|urinary tract infection|urticaria|vascular disease|ventricular fibrillation|vertigo|visual field defect|vomiting
63	methoxsalen	-0.49339515	1.94991	4114	
64	dicloxacillin	-0.49153617	1.9291	18381	esophageal:myoclonus:rash:angioedema:bronchospasm:hematuria:fever:lethargy:proteinuria:laryngospasm:cholestatic hepatitis:stomatitis:hypersensitivity:leukopenia:nephropathy:confusion:ulcer:gastrointestinal irritation:edema:SGOT increased:sickness:pruritus:hemolytic anemia:abdominal pain:esophagitis:interstitial nephritis:urticaria:agranulocytosis:anemia:abnormal liver function:renal insufficiency:thrombocytopenia:death:liver function tests abnormal:nephritis:tubular:diarrhea:vomiting:shock:seizures:twitching:hepatitis:laryngeal edema:pain:marrow depression:gastrointestinal reactions:hairy tongue:colitis:anaphylaxis:nausea:pseudomembranous colitis:myalgia:esophageal ulcer:sensitization:arthralgia:neutropenia:eosinophilia:hypotension:serum sickness:malaise
65	pepstatin	-0.61058791	1.92082	5478883	

66	phensuximide	-0.48923979	1.9036	6839	
67	L-methionine	-0.48898828	1.90083	16118	
68	heptaminol	-0.62869635	1.88606	10969	
69	PF-00539745-	-0.60163082	1.88606		
70	ciclosporin	-0.58579898	1.85387	5284373	abdominal cramps|abdominal discomfort|abdominal pain|abnormal dreams|abnormal ejaculation|abnormal gait|abnormal glucose|abnormal glucose tolerance|abnormality of accommodation|abnormal lacrimation|abnormal liver function|abnormal vision|abscess|accidental pregnancy|aches|acne|acute psychosis|acute renal failure|aggravated depression|aggravated hypertension|aggressive|aggressive reaction|agranulocytosis|akathisia|alcohol intolerance|alkaline phosphatase increased|alopecia|amenorrhea|amnesia|anaphylaxis|anemia|anger|angina|angioedema|anorexia|anorgasmia|anxiety|anxiety attack|apathy|aplastic anemia|appendicitis|arrest|arrhythmia|artery disease|arthralgia|arthritis|arthrosis|asthenia|asthma|ataxia|atrial fibrillation|auditory hallucinations|autonomic instability|Autonomic Nervous System Disorders|back discomfort|back pain|bacterial infections|bilirubinemia|bloating|blood in urine|blunted affect|blurred vision|bone disorder|bone pain|bradycardia|Breast Disorders|breast enlargement|breast neoplasm|Breast neoplasm malignant female|breast pain|bronchitis|bronchospasm|bruxism|bundle branch block|bursitis|cancer|carbohydrate craving|cardiovascular disease|carpal tunnel syndrome|cataract|catatonic|cellulitis|cerebrovascular accident|cerebrovascular disorder|cervical lymphadenopathy|chest pain|chest tightness|chills|cholecystitis|cholelithiasis|choreoathetosis|Clotting Disorders|colitis|Collagen Disorders|coma|completed suicide|confusion|conjunctivitis|Connective Tissue Disorders|constipation|convulsions|coronary artery disease|cough|cramps|crying abnormal|cystitis|death|decreased appetite|decreased drug level|decreased libido|decreased prothrombin time|decreased sweating|deep vein thrombosis|dehydration|delirium|delusions|Dependent edema|depersonalization|derealization|dermatitis|diabetes|diarrhea|diplopia|discomfort|disorientation|diverticulitis|dizziness|drug abuse|drug dependence|drug level increased|dry eyes|dry lips|dry mouth|dry skin|duodenal ulcer|dys|dysarthria|dyskinesia|dysmenorrhea|dyspepsia|dysphagia|dysphonia|dyspnea|dystonia|dysuria|ear pain|ecchymosis|ECG abnormal|eczema|edema|ejaculation disorder|ejaculation failure|elevated bilirubin|elevated liver enzymes|embolism|emotional lability|Endocrine Disorders|enlargement|eosinophilia|epidermal necrolysis|epistaxis|equilibrium disorders|eructation|erythema|erythema multiforme|esophagitis|euphoria|excitability|extrapyramidal syndrome|extrasystoles|Eye

71	vorinostat	-0.58165738	1.85387	5311	
72	deptropine	-0.57472572	1.85387	16576	
73	ikarugamycin	-0.57194823	1.85387	54714554	agranulocytosis|angioedema|confusion|diarrhea|dizziness|edema|epigastric distress|erythema|erythema multiforme|fatigue|headache|hypersensitivity|insomnia|leukopenia|nausea|oral moniliasis|paresthesia|proteinuria|rash|urticaria|vomiting
74	velnacrine	-0.48388155	1.84505	60576	
75	myricetin	-0.48285364	1.83397	5281672	
76	streptomycin	-0.60676265	1.82391	19649	rash:angioedema:paresthesia:deafness:fever:Amblyopia:azotemia:thrombocytopenia:muscle weakness:edema:inability to walk:hemolytic anemia:urticaria:anemia:weakness:exfoliative dermatitis:dermatitis:vertigo:vomiting:renal impairment:pancytopenia:anaphylaxis:nausea:eosinophilia:leukopenia
77	xamoterol	-0.59222975	1.82391	155774	
78	fursultiamine	-0.58495474	1.82391	3002119	

79	adenosine ph	-0.48186948	1.8234	6083	abdomen enlarged|abdominal discomfort|abdominal pain|abnormal gait|abnormal liver function|acne|acute renal failure|agitation|agranulocytosis|albuminuria|alkaline phosphatase increased|alopecia|Amblyopia|amenorrhea|amnesia|amyloidosis|anemia|angina|angioedema|anorexia|anxiety|aphthous stomatitis|aplastic anemia|apnea|arm pain|arrest|arrhythmia|arterial thrombosis|arthralgia|arthritis|arthrosis|ascites|asthenia|ataxia|atrial fibrillation|avitaminosis|back pain|bilirubinemia|bloating|blood glucose increased|bone pain|bone tenderness|bradycardia|breast discomfort|Breast swelling|bronchitis|BUN increased|burning sensation|causalgia|cellulitis|cerebral ischemia|cerebrovascular accident|cervical erosion|chills|chills and fever|cholangitis|cholestasis|cholestatic jaundice|chronic myeloid leukemia|circumoral paresthesia|colitis|confusion|congestive heart failure|conjunctivitis|constipation|convulsions|coronary thrombosis|cough|cramps|creatinine increased|cyanosis|cyst|cystitis|deafness|death|decreased creatinine|decreased glucose|decreased libido|decreased phosphorus|deep thrombophlebitis|dehydration|dementia|demyelination|dermatitis|desquamation|diabetes|diarrhea|diplopia|disability|discomfort|dry eyes|dry mouth|dry skin|dysesthesia|dyskinesia|dysmenorrhea|dyspareunia|dyspepsia|dysphagia|dysuria|eczema|edema|effusion|electrolyte imbalance|elevated blood pressure|elevated liver function tests|elevated serum creatinine|emotional lability|emphysema|Endocrine Disorders|endometriosis|enlargement|enuresis|eosinophilia|epidermal necrolysis|epistaxis|eructation|eruptions|erythema multiforme|erythroleukemia|esophagitis|euphoria|exfoliative dermatitis|Eye Disorders|eye pain|face edema|fatigue|febrile neutropenia|fever|fibrillation|flatulence|flu|fluid retention|flushing|foot drop|fungal dermatitis|galactorrhea|gallbladder disease|gastric ulcer|gastroenteritis|Gastrointestinal Disorders|gastrointestinal hemorrhage|glossitis|glucose tolerance decreased|grand mal|gum disorder|gynecomastia|hangover|headache|heart failure|hematuria|hemoptysis|hemorrhagic eruption|hepatitis|hepatomegaly|hernia|herpes|herpes simplex|hirsutism|hormone level altered|hostility|hypalgesia|hypercalcemia|hypercholesterolemia|hyperesthesia|hyperglycemia|hyperkalemia|hyperkinesia|hyperlipidemia|hypernatremia|hypertension|hypertonia|hyperuricemia|hypesthesia|hypocalcemia|hypochromic anemia|hypoglycemia|hypokalemia|hypomagnesemia|hyponatremia|hypoproteinemia|h
80	fluorocurarine	-0.47762663	1.77833	201810	

81	vanoxerine	-0.57371383	1.76955	5361022	acne|acneiform eruptions|allergic contact dermatitis|application site reaction|atopic|atopic dermatitis|atrophy|Burning Itching|condition aggravated|contact dermatitis|desquamation|dizziness|dry skin|eruptions|erythema|folliculitis|glycosuria|headache|hyperglycemia|hypersensitivity|hypertrichosis|hypopigmentation|Immune System Disorders|infection|miliaria|nasal congestion|Nasopharyngitis|Nervous System Disorders|pain|pruritus|rash|secondary infection|skin atrophy|striae|swelling|tightness of skin
82	colistin	-0.47640189	1.76547	5311054	abdominal discomfort|abdominal wall disorder|acute hepatic failure|acute kidney failure|adenovirus infection|alpha haemolytic streptococcal infection|angiopathy|anterior chamber cell|anterior chamber flare|appendectomy|appendicitis|appendix disorder|bacterial toxaemia|blepharitis|blood bilirubin increased|blood creatinine increased|Bone marrow fibrosis|burkholderia cepacia infection|cardiogenic shock|chest wall abscess|chorioretinal disorder|chronic obstructive airway disease|CMV infection|conjunctival deposit|corynebacterium infection|cytolytic hepatitis|cytomegalovirus hepatitis|death|drug resistance|erosive gastritis|eruption|eyelid infection|eye pain|fat tissue increased|fungal endocarditis|gamma-glutamyltransferase increased|gastroenteritis|gastrointestinal toxicity|haemodialysis|haemolytic anaemia|haemorrhoids|hepatic artery thrombosis|Hepatic failure|hepatotoxicity|international normalised ratio increased|intracardiac thrombus|klebsiella infection|lacrimation increased|lung infiltration|maculopathy|metabolic acidosis|multi-organ disorder|neumonia|neutropenic colitis|neutropenic sepsis|non-cardiac chest pain|pathogen resistance|pericardial rub|post procedural bile leak|pseudomonas infection|renal cyst|respiratory failure|retinal exudates|retinal infarction|retinal scar|scotoma|septic shock|therapeutic response decreased|treatment failure|vitrectomy
83	naftifine	-0.47446636	1.74528	47641	erythema:rash:skin tenderness:pruritus:tenderness:stinging
84	valinomycin	-0.59175463	1.74473	8909	
85	**kaempferol**	**-0.57972207**	**1.74473**	5280863	
86	5213008	-0.59259784	1.72125	5213008	

87	sulmazole	-0.47199501	1.71975	5353	abdominal pain|abnormal liver function|agranulocytosis|alkaline phosphatase increased|anaphylaxis|anemia|aplastic anemia|chills|cholestasis|colitis|coma|confusion|constipation|convulsive status epilepticus|decreased calcium|decreased phosphorus|diarrhea|disability|dizziness|dyspnea|encephalopathy|eosinophilia|epidermal necrolysis|erythema|erythema multiforme|febrile neutropenia|fever|gastrointestinal symptoms|genital pruritus|hallucinations|headache|hemolysis|hemolytic anemia|hemorrhage|hepatic failure|hepatic impairment|hypersensitivity|hypocalcemia|increased calcium|inflammation|injection site inflammation|leukopenia|Local Reactions|moniliasis|myoclonus|nausea|nephropathy|nonconvulsive status epilepticus|oral moniliasis|pain|pancytopenia|paresthesia|phlebitis|potassium increased|prolonged prothrombin time|pruritus|pseudomembranous colitis|rash|renal failure|renal impairment|renal insufficiency|seizures|SGPT increased|status epilepticus|stupor|taste perversion|thrombocytopenia|toxic epidermal necrolysis|toxic nephropathy|urticaria|vaginal moniliasis|vaginitis|vasodilation|vomiting
88	probenecid	-0.47037661	1.70318	4911	alopecia:hematuria:fever:hypersensitivity:nephrotic syndrome:urinary frequency:dizziness:pruritus:hemolytic anemia:urticaria:tenderness:anemia:aplastic anemia:dermatitis:sore gums:vomiting:pain:hepatic necrosis:anaphylaxis:nausea:anorexia:renal colic:colic:necrosis:flushing:leukopenia:headache
89	amantadine	-0.59880941	1.69897	2130	decreased visual acuity:fever:delusions:confusion:insomnia:hypertension:pruritus:dry mouth:arrhythmia:tachypnea:manic reaction:urinary retention:suicidal ideation:decreased libido:nausea:corneal edema:corneal opacity:eczema:paranoid reaction:paresthesia:leukopenia:slurred:acute respiratory failure:involuntary muscle contractions:hypersensitivity:somnolence:optic neuropathy:edema:dizziness:ataxia:fatigue:weakness:stupor:nasal dryness:diarrhea:hypersexuality:pulmonary edema:anaphylaxis:tremor:keratitis:rash:constipation:agitation:irritability:heart failure:congestive heart failure:nervousness:abnormal gait:libido increased:arrest:agranulocytosis:tachycardia:dermatitis:convulsions:peripheral edema:livedo reticularis:respiratory failure:dysphagia:delirium:anorexia:hypertonia:hyperkinesia:manic:livedo:pathological gambling:amnesia:coma:malignant syndrome:suicide:lightheadedness:neutropenia:euphoria:aggressive:dyspnea:postural hypotension:neuroleptic malignant:visual disturbances:vomiting:anxiety:psychosis:hypokinesia:leukocytosis:hallucinations:nerve palsy:increased sweating:mydriasis:hypotension:headache

90	naftopidil	-0.59056952	1.69897	11957660	
91	5186223	-0.58736331	1.67778	5186223	
92	piretanide	-0.46686642	1.66763	4849	abdominal discomfort|abdominal pain|abnormal ECG|acholia|agranulocytoses|allergic vasculitis|allergies|anaemia|antidepressant drug level increased|aortic dilatation|apathy|aphthous stomatitis|arteriosclerotic heart disease|ascites|Atrioventricular block complete|atrophy|benign gastric neoplasm|biopsy bone marrow|bladder cancer|bladder disorder|blood glucose decreased|body temperature|bone marrow failure|bone marrow tumour cell infiltration|bone pain|bradycardia|bundle branch block right|cardiac enlargement|cardiac failure|cardiogenic shock|cardiovascular disorder|central venous catheterisation|cerebral artery occlusion|cerebral infarct|chemotherapeutic drug level increased|choluria|colitis pseudomembranous|condition aggravated|congenital mitral valve incompetence|conjunctival deposit|coronary artery occlusion|coronary artery stenosis|coughing blood|dermatitis seborrheic|Diabetes|Diabetic neuropathy|diarrhea|difficulty in walking|disease recurrence|dizziness exertional|drug interaction|dry skin|edema extremities|Electrolyte disorder|endoscopy abnormal|erosive gastritis|Excess potassium|exercise electrocardiogram abnormal|fatty liver|gastric disorder|Glucose intolerance|goiter|heart attack|heart valve replacement|helicobacter gastritis|Hepatic failure|hepatosplenic t-cell lymphoma|hypothyroid|idiopathic thrombocytopenic purpura|inflammatory bowel disease|Interstitial nephritis|intervertebral disc disorder|intervertebral disc operation|intra-abdominal haemorrhage|itch|kidney failure|leg amputation|leucopenia|leukopenia neonatal|lung infiltration|Lymphocytes decreased|micturition disorder|monoplegia|mucosal inflammation|muscle weakness|neurological examination abnormal|oesophageal candidiasis|pain in throat|pancreas lipomatosis|pancreatitis|peritonitis bacterial|Phlebitis|plasmacytoma|pneumonia fungal|polyneuropathy|renal impairment|renal lipomatosis|renal tubular disorder|renal tubular necrosis|rhabdomyolysis|Rhagades|sjogren's syndrome|spinal disorder|splenectomy|sputum discoloured|Supraventricular tachycardia|uterine prolapse|venous thrombosis limb|vitamin b1 decreased|vitamin b1 deficiency
93	trimethadione	-0.46673519	1.66632	5576	
94	clotrimazole	-0.58103668	1.65758	2812	erythema:rash:blisters:peeling:hemorrhage:edema:SGOT increased:urinary frequency:skin irritation:pruritus:urticaria:abnormal liver function:bloating:liver function tests abnormal:burning sensation:vomiting:stinging:nausea:vaginal burning:cramps:redness:abdominal cramps:cystitis
95	chenodeoxych	-0.56805265	1.65758	10133	
96	clofilium tosyl	-0.56235682	1.65758	175533	

97	glimepiride	-0.4654011	1.65296	3476	vasculitis:porphyria:jaundice:photosensitivity reaction:paresthesia:hot flushes:erythema:gastrointestinal pain:erythrocytopenia:eruptions:hypersensitivity:thrombocytopenia:hepatitis:nervousness:hypoglycemic episodes:increased appetite:laboratory test abnormal:allergic skin reaction:urinary frequency:dizziness:pruritus:hemolytic anemia:hypersensitivity vasculitis:agranulocytosis:cutaneous hypersensitivity:urticaria:dyspnea:dry mouth:anemia:Hepatic porphyria:aplastic anemia:nocturia:increased sweating:insomnia:dyspepsia:asthenia:cholestasis:diarrhea:vomiting:sweating:hepatic failure:palpitations:pain:pancytopenia:hepatic impairment:Pain in extremity:anorexia:SIADH:gastrointestinal reactions:nausea:abdominal pain:porphyria cutanea tarda:photosensitivity:tremor:blurred vision:vasodilation:hyponatremia:hypoglycemic reaction:hypotension:shock:leukopenia:headache
98	cefalotin	-0.57499442	1.63827	6024	
99	resveratrol	-0.46374989	1.63653	445154	
100	STOCK1N-356	-0.46306098	1.62972		
101	tranexamic ac	-0.46147537	1.6141	5526	chromatopsia:atopic dermatitis:migraine:vein thrombosis:gastrointestinal symptoms:necrosis:back pain:thrombosis:dizziness:vein occlusion:abdominal pain:abnormal vision:musculoskeletal pain:obstruction:dermatitis:convulsions:visual disturbances:diarrhea:vomiting:pulmonary embolism:giddiness:pain:anemia:thromboembolic events:nausea:embolism:cramps:arthralgia:fatigue:hypotension:deep vein thrombosis:cerebral thrombosis:retinopathy:renal cortical necrosis:headache
102	ticarcillin	-0.5603848	1.60206	36921	
103	hydroflumeth	-0.45951798	1.59499	3647	vasculitis:withdrawn:rash:constipation:agitation:pancreatitis:purpura:hypersensitivity:thrombocytopenia:hyperglycemia:hyperuricemia:dizziness:jaundice:agranulocytosis:urticaria:anemia:gastric irritation:aplastic anemia:postural hypotension:weakness:cholestatic jaundice:vertigo:diarrhea:vomiting:glycosuria:spasm:necrotizing angiitis:nausea:anorexia:photosensitivity:cramps:xanthopsia:cutaneous vasculitis:hypotension:leukopenia:headache
104	scopoletin	-0.45910244	1.59095	5280460	
105	lasalocid	-0.592351	1.58503	2724110	
106	estropipate	-0.52666331	1.58503	5284555	

107	cefotiam	-0.45849007	1.58501	43708	abdominal tenderness|abnormal behaviour|abnormal sensation in eye|Abnormal vision|abortion|abscess|abscess drainage|accident|acute abdomen|acute brain syndrome|acute bronchitis|acute pancreatitis|acute respiratory failure|acute tonsillitis|adenosquamous cell lung cancer|ADH inappropriate|adrenal insufficiency neonatal|adynamic ileus|affect lability|agranulocytoses|alanine aminotransferase increased|altered state of consciousness|alveolar proteinosis|anaemia|anaemia postoperative|anal haemorrhage|anaphylactic reaction|anorectal operation|aphagia|Apnea|appendicitis|arteriospasm coronary|Aspartate Aminotransferase Increase|aspergillosis|aspiration pneumonia|athetosis|autoimmune hepatitis|automatism|Bacterial infection|bacterial toxaemia|bacteria sputum identified|bacteria urine identified|band neutrophil percentage decreased|bence jones protein urine|black stools|Bladder inflammation|bleeding time prolonged|blood albumin decreased|blood alkaline phosphatase increased|blood amylase decreased|blood bilirubin increased|blood brain barrier defect|blood cholinesterase decreased|blood creatine phosphokinase increased|blood immunoglobulin m|blood lactate dehydrogenase decreased|blood lactate dehydrogenase increased|blood potassium increased|blood pressure immeasurable|blood pressure systolic decreased|blood pressure systolic increased|blood sodium abnormal|body temperature increased|bone marrow depression|bone marrow failure|bovine tuberculosis|brain death|brain herniation|bronchitis|bulging|burning sensation mucosal|cancer pain|candida sepsis|Carcinoma of Prostate|cardiac failure|cardiac failure chronic|cardiac output decreased|cardiogenic shock|catheter placement|catheter related infection|cellulitis|cerebral haemorrhage|cerebral infarct|chill|chorioamnionitis|clamping of blood vessel|clostridium difficile colitis|CMV infection|colitis ischaemic|colitis pseudomembranous|condition aggravated|convulsion|c-reactive protein increased|cryptogenic organising pneumonia|csf monocyte count increased|csf neutrophil count increased|cyanosis|decubitus ulcer|delayed recovery from anaesthesia|dental pain|depressed level of consciousness|dermatitis medicamentosa|Diabetic Nephropathy|disease of liver|disease recurrence|disseminated intravascular coagulation|disseminated intravascular coagulation in newborn|drug effect prolonged|drug exposure during pregnancy|dysuria|electrocardiogram qt prolonged|electrocardiogram st segment depression|electrocardiogram st segment elevation|encephalitis herpes|endoscopy abnormal|enterococcal infection|Eosinophil Count Increased|erythema|erythema annulare|escherichia sepsis|esophageal ulcer|excessive thirst|Excess potassium|fibrin degradation products

108	nicotinic acid	-0.4582823	1.583	938	bilirubinemia:fever:myasthenia:contact dermatitis:acne:esophagitis:twitching:mucous membrane:hyperuricemia:gallstone:acanthosis:urticaria:dry mouth:arrhythmia:generalized edema:urinary retention:cough increased:face edema:macular edema:abnormal vision:nausea:syncope:eczema:alopecia:herpes:anorexia:atrial fibrillation:pigmentation:flushing:tumor:leukopenia:acanthosis nigricans:tongue edema:bursitis:fungal dermatitis:hyperpigmentation:blurred vision:chills:cough:Amblyopia:hypersensitivity:skin ulcer:somnolence:edema:xanthomas:dizziness:bone disorder:vaginitis:abnormal liver function:asthenia:vertigo:diarrhea:tinnitus:burning sensation:bronchitis:gastrointestinal hemorrhage:skin neoplasm:myopathy:zoster:hemoptysis:skin burning sensation:anaphylaxis:Eye Disorders:Gastrointestinal Disorders:lung disorder:hypesthesia:shock:rhabdomyolysis:gout:rash:eructation:glucose tolerance decreased:jaundice:paresthesia:tongue disorder:hyperventilation:laryngospasm:laryngitis:stomatitis:dry skin:nervousness:ulcer:pruritus:libido increased:hernia:skin discoloration:tingling:flu:glaucoma:tachycardia:dermatitis:liver function tests abnormal:sweating:vesiculobullous rash:decreased glucose:peripheral edema:palpitations:abdomen enlarged:dysphagia:incontinence:hypertonia:hyperkinesia:migraine:cramps:cystoid macular edema:application site reaction:polyuria:redness:angioedema:rhinitis:gastrointestinal symptoms:hepatitis:maculopapular rash:peptic ulcer:flatulence:dyspepsia:cardiovascular disease:dyspnea:fecal incontinence:postural hypotension:angina:prostate:insomnia:vomiting:hemorrhage:breast pain:laryngeal edema:cyst:myalgia:hypokinesia:arthralgia:fibrillation:impotence:arthritis:hypotension:headache:leg cramps

109	ketoconazole	-0.45800892	1.58036	456201	erythema:contact dermatitis:photophobia:paresthesia:skin irritation:chills and fever:hypertriglyceridemia:fever:chills:pyogenic granuloma:rash:eye irritation:hypersensitivity:sicca:thrombocytopenia:application site reaction:acne:somnolence:keratoconjunctivitis:keratoconjunctivitis sicca:dizziness:pruritus:hemolytic anemia:abdominal pain:granuloma:urticaria:anemia:impetigo:abnormal liver function:gynecomastia:oligospermia:swelling:arrhythmia:dry skin:abnormal hair texture:papilledema:intracranial hypertension:diarrhea:burning sensation:vomiting:eye swelling:stinging:pain:swelling of the face:pustules:skin burning sensation:anaphylaxis:nausea:alopecia:QT prolonged:sensitization:Bulging fontanels:nail discoloration:impotence:hair discoloration:scalp seborrhea:leukopenia:headache
110	ajmaline	-0.54490126	1.55284	11972341	
111	verteporfin	-0.45437845	1.54559	5362420	decreased visual acuity:extravasation:back pain:diplopia:fever:myasthenia:blindness:hypertension:pruritus:urticaria:blepharitis:anemia:injection site pain:chest pain:sleep disorder:urogenital disorder:visual field defect:pain:nausea:syncope:eczema:conjunctivitis:atrial fibrillation:varicose vein:glycosuria:elevated liver function tests:flushing:hypesthesia:injection site reaction:photosensitivity:pneumonia:body odor:blurred vision:cough:hypersensitivity:edema:dizziness:detachment:injection site hypersensitivity:pharyngitis:musculoskeletal pain:asthenia:vertigo:hypercholesterolemia:injection site hemorrhage:arthrosis:gastrointestinal cancers:conjunctival hyperemia:injection site inflammation:retinal detachment:malaise:rash:constipation:changes in blood pressure:skin disorder:lacrimation disorder:inflammation:vascular disease:General symptoms:flu:cataract:photosensitivity reaction:lacrimation:creatinine increased:vitreous hemorrhage:eye itching:albuminuria:hemorrhage:sweating:WBC Decreased:dyspnea:white blood cell count increased:cancer:prostate:visual disturbances:hearing loss:peripheral vascular disorder:dry eyes:ketosis:arthralgia:fibrillation:eosinophilia:headache
112	amikacin	-0.55975523	1.5376	37768	drug fever:rash:renal impairment:paresthesia:deafness:fever:apnea:toxic nephropathy:azotemia:albuminuria:nephropathy:blindness:acute renal failure:infarction:renal failure:anemia:hypomagnesemia:oliguria:hearing loss:vomiting:hypotension:paralysis:nausea:tremor:arthralgia:high frequency hearing loss:eosinophilia:headache
113	furaltadone	-0.45315371	1.53399	9553856	
114	meglumine	-0.55038159	1.52288	8567	
115	caffeic acid	-0.44924985	1.49745	3449	

116	iohexol	-0.55113076	1.48149	3730	swelling:hematoma:fever:lightheadedness:somnolence:caries:muscle weakness:hypertension:reactions to contrast media:flatulence:weakness:diarrhea:vomiting:pain:distention:gastrointestinal reactions:nausea:syncope:tremor:uterus:headache
117	ethionamide	-0.53969828	1.48149	2761171	weight loss:rash:jaundice:agitation:pellagra:increased salivation:blurred vision:purpura:acne:stomatitis:hypersensitivity:thrombocytopenia:hepatitis:salivation:optic neuritis:hypoglycemia:dizziness:abdominal pain:hypothyroidism:postural hypotension:diplopia:diarrhea:diabetes:vomiting:drowsiness:metallic taste:mental depression:pain:nausea:anorexia:photosensitivity:impotence:hypotension:gynecomastia:neuritis:headache
118	azapropazone	-0.44741274	1.48048	46937068	
119	tocainide	-0.56078321	1.46852	38945	
120	altizide	-0.51898804	1.4437	2122	

121	debrisoquine	-0.44203263	1.43164	2966	abdomen enlarged|abdominal pain|abnormal gait|abnormal renal function|abnormal vision|abscess|acne|acral erythema|acute renal failure|aggressive|agitation|agranulocytosis|AIDSrelated Kaposi Sarcoma|albuminuria|alkaline phosphatase increased|alopecia|Amblyopia|amenorrhea|AML|anaphylactoid reactions|anaphylaxis|anemia|angina|anorexia|anxiety|aphthous stomatitis|arrest|arrhythmia|arthralgia|ascites|aspiration|asthenia|ataxia|azoospermia|balanitis|bilirubinemia|blepharitis|blindness|blisters|bone pain|bradycardia|breast pain|bronchitis|bundle branch block|burning sensation|cachexia|cardiomegaly|cardiomyopathy|cellulitis|chills|chills and fever|cholangitis|cholestatic jaundice|colitis|coma|confusion|congestive heart failure|conjunctivitis|Connective Tissue Disorders|constipation|contact dermatitis|convulsions|cough|cough increased|cryptococcosis|cutaneous candidiasis|death|deep thrombophlebitis|dehydration|delirium|dermatitis|diabetes|diarrhea|dizziness|drug abuse|dry eyes|dry mouth|dry skin|dysesthesia|dyspepsia|dysphagia|dysphonia|dysuria|early menopause|ear pain|ecchymosis|edema|effusion|elevated bilirubin|elevated transaminases|elevated uric acid|embolism|emotional lability|encephalopathy|eosinophilia|epidermal necrolysis|epistaxis|eructation|erythema|erythema multiforme|erythema nodosum|esophageal|esophageal ulcer|esophagitis|exfoliative dermatitis|extravasation|eye pain|face edema|fatigue|febrile neutropenia|fecal impaction|fever|flatulence|flu|flushing|folliculitis|fungal dermatitis|furunculosis|gastric erosions|gastric ulcer|gastritis|Gastrointestinal Disorders|gastrointestinal hemorrhage|generalized edema|generalized rash|genital edema|gingivitis|glossitis|glycosuria|gum hemorrhage|hand and foot syndrome|heart failure|Hematologic Disorders|hematuria|hemiplegia|hemolysis|hemoptysis|hemorrhage|hepatic failure|hepatitis|hepatosplenomegaly|herpes|herpes simplex|hiccup|hot flushes|hydronephrosis|hypercalcemia|hyperesthesia|hyperglycemia|hyperkalemia|hyperlipidemia|hypermagnesemia|hypernatremia|hyperpigmentation|hypertension|hypertonia|hyperuricemia|hyperventilation|hypesthesia|hypocalcemia|hypochloremia|hypochromic anemia|hypoglycemia|hypokalemia|hypokinesia|hypolipemia|hypomagnesemia|hyponatremia|hypophosphatemia|hypoproteinemia|hypothermia|hypotonia|hypoxia|ileus|impaction|incontinence|incoordination|increased appetite|increased

122	nabumetone	-0.44197796	1.43115	4409	gastritis:erythema:rectal hemorrhage:fever:myocardial infarction:bullous eruption:acne:azotemia:nephrotic syndrome:thrombocytopenia:Stevens Johnson Syndrome:hypertension:hyperuricemia:pruritus:urticaria:renal failure:dry mouth:anemia:jaundice:arrhythmia:anaphylactoid reactions:gastroenteritis:paresthesia:hepatic failure:pain:nausea:syncope:alopecia:photosensitivity:interstitial pneumonitis:eosinophilic pneumonia:duodenal ulcer:hypokalemia:duodenitis:elevated liver function tests:leukopenia:vaginal bleeding:gingivitis:pneumonia:chills:interstitial nephritis:flatulence:hypersensitivity:somnolence:hyperglycemia:edema:nightmares:dizziness:nephritic syndrome:abnormal liver function:fatigue:asthenia:vertigo:diarrhea:tinnitus:dysuria:kidney calculus:confusion:gastrointestinal hemorrhage:anaphylaxis:tremor:peptic ulcer:allergic pneumonitis:glossitis:malaise:vasculitis:weight loss:weight gain:rash:eructation:constipation:agitation:hematuria:stomatitis:nervousness:ulcer:agranulocytosis:stool guaiac:cholestatic jaundice:sweating:nephritis:asthma:palpitations:gastric ulcer:pseudoporphyria:dysphagia:thrombophlebitis:anorexia:increased appetite:aplastic anemia:erythema multiforme:angioedema:toxic epidermal necrolysis:pancreatitis:eruptions:cough:albuminuria:hemorrhage:dyspepsia:infarction:abdominal pain:dyspnea:abnormal vision:cholelithiasis:angina:insomnia:epidermal necrolysis:vomiting:anxiety:impotence:increased sweating:melena:headache

123	indapamide	-0.44180299	1.42959	3702	gastritis:erythema:back pain:ear pain:ventricular extrasystoles:fever:myasthenia:bullous eruption:kidney pain:acne:esophagitis:respiratory distress:twitching:urinary tract infection:hyperuricemia:urinary frequency:pruritus:bone pain:urticaria:renal failure:dry mouth:anemia:gastric irritation:jaundice:chondrodystrophy:arrhythmia:gastroenteritis:proctitis:chest pain:sialadenitis:bronchitis:nodules:face edema:pain:decreased libido:bundle branch block:tooth disorder:nausea:photosensitivity:pharyngitis:bradycardia:syncope:hyponatremia:atrial flutter:tension:hypokalemia:U wave:glycosuria:flushing:flutter:leukopenia:gastrointestinal pain:cerebrovascular accident:nocturia:conjunctivitis:numbness:pneumonia:sinusitis:irregular heart rate:blurred vision:sinus bradycardia:purpura:duodenitis:cough:flatulence:myopia:hyperesthesia:somnolence:hyperglycemia:atrial fibrillation:nail disorder:optic neuritis:dizziness:ataxia:bone disorder:gastric pain:hypochloremia:abnormal liver function:fatigue:rhinorrhea:weakness:asthenia:vertigo:diarrhea:tinnitus:skin nodule:dysuria:diabetes:carcinoma:oral moniliasis:spasm:moniliasis:myopathy:anaphylaxis:arthrosis:xanthopsia:lung disorder:rhabdomyolysis:malaise:vasculitis:weight loss:gout:rash:constipation:photophobia:agitation:irritability:lethargy:laryngitis:stomatitis:nervousness:mouth Tingling:hernia:tingling:supraventricular tachycardia:flu:agranulocytosis:extrasystoles:oliguria:tachycardia:cholestatic jaundice:dermatitis:liver function tests abnormal:dyspepsia:electrolyte imbalance:dysmenorrhea:nephritis:peripheral edema:Abnormal thyroid:palpitations:dysphagia:ecchymosis:anorexia:hypertonia:migraine:cramps:cataract:application site reaction:increased appetite:polyuria:vaginitis:aplastic anemia:drowsiness:infection:neuritis:ventricular arrhythmia:erythema multiforme:angioedema:rhinitis:amnesia:blisters:coma:exfoliative dermatitis:pancreatitis:eruptions:lightheadedness:interstitial nephritis:thrombocytopenia:hepatitis:ear disorder:Amblyopia:sweating:otitis:abdominal pain:dyspnea:necrotizing angiitis:postural hypotension:angina:prostate:insomnia:visual disturbances:taste perversion:epidermal necrolysis:vomiting:anxiety:numbness of extremities:paresthesia:myalgia:hyperosmolar coma:loss of consciousness:arthralgia:vasodilation:fibrillation:impotence:arthritis:hypotension:headache

124	selegiline	-0.44137652	1.42577	26757	gastritis:lymphadenopathy:ear pain:rectal hemorrhage:yawning:myasthenia:delusions:twitching:aches:dry mouth:anemia:hypoproteinemia:lung carcinoma:urogenital disorder:urinary retention:visual field defect:circumoral paresthesia:tumor:alopecia:conjunctivitis:paranoid reaction:bradycardia:burning lips:cataract specified:cerebrovascular accident:gingivitis:lactic dehydrogenase increased:body odor:chills:somnolence:myalgia:nightmares:ankle edema:vertigo:tinnitus:diabetes:bronchitis:flank pain:enlargement:hypercholesterolemia:disorientation:pneumothorax:gout:eructation:Female Sexual Dysfunction:aphasia:paresthesia:dyskinesia:cheilitis:breast carcinoma:Falling Down:changes in blood pressure:involuntary movements:mean blood pressure:cerebral ischemia:chronic renal failure:glaucoma:tachycardia:ischemia:palpitations:dysphagia:delirium:incontinence:dystonia:esophageal ulcer:application site reaction:manic:drowsiness:neuritis:skin hypertrophy:rhinitis:hiccup:otitis:bradykinesia:renal failure:taste loss:alcohol intolerance:cholelithiasis:angina:artery occlusion:hepatitis:peripheral vascular disorder:neuropathy:vasodilation:impotence:mydriasis:fibrillation:parasitic infection:fever:geriatric:cellulitis:acne:esophagitis:prostate hypertrophy:hyperpyrexia:leg pain:hypertension:pruritus:urticaria:arrhythmia:death:gastroenteritis:face edema:pain:memory impairment:metrorrhagia:decreased libido:benign neoplasm:nausea:eczema:otitis externa:hyponatremia:hypokalemia:pleural effusion:kidney calculus:leukopenia:low back pain:ovary:urination impaired:cough:skin ulcer:pharyngitis:caries:dizziness:ataxia:detachment:visual hallucinations:abnormal liver function:weakness:gamma glutamyl transpeptidase increased:diarrhea:AV block:myocardial ischemia:encephalopathy:hypersexuality:apraxia:myopathy:zoster:melanoma:arthrosis:bacterial infections:weight loss:weight gain:epididymitis:tongue edema:constipation:emotional lability:deafness:tongue disorder:hyperlipidemia:trismus:arrest:vivid dreams:dementia:dyspepsia:fungal dermatitis:dysmenorrhea:peripheral edema:voice alteration:ecchymosis:carcinoma:increased appetite:polyuria:hematoma:subdural hematoma:skin benign neoplasm:amenorrhea:vaginal moniliasis:albuminuria:leukocytosis:Amblyopia:dehydration:sweating:abdominal pain:dyspnea:postural hypotension:grimacing:neck rigidity:periodontal abscess:vomiting:apathy:increased angina:tenosynovitis:incoordination:increased
125	cantharidin	-0.54157565	1.42022	2545	

126	levodopa	-0.44044703	1.41749	6047	abdomen enlarged|abdominal cramps|abdominal discomfort|abdominal distention|abdominal pain|abdominal pain upper|abnormal liver function|abnormal vision|abscess|aches|acne|agitation|alkaline phosphatase increased|allergic contact dermatitis|amenorrhea|amnesia|anaphylactoid reactions|anaphylaxis|angioedema|anxiety|application site reaction|arthralgia|arthritis|arthritis aggravated|arthrosis|asthenia|asthma|back pain|bacterial infections|basal cell carcinoma|bladder disorder|bladder infection|blepharitis|bloating|blood glucose increased|bone disorder|bone pain|breast discharge|breast discomfort|Breast Disorders|breast engorgement|breast enlargement|breast malformation|breast mass|breast neoplasm|breast pain|Breast swelling|breast tenderness|bronchitis|bronchospasm|carpal tunnel syndrome|cerebrovascular disorder|cervical ectropion|cervical erosion|chest pain|cholecystitis|cholelithiasis|cholestatic jaundice|chorea|clammy|common cold|conjunctivitis|Connective Tissue Disorders|constipation|contact dermatitis|contact lens intolerance|coronary thrombosis|cough|cough increased|cramps|cyst|cystitis|Decreased carbohydrate tolerance|decreased glucose|decreased libido|dermatitis|device complication|diarrhea|discomfort|diverticulitis|dizziness|dysmenorrhea|dyspepsia|ear disorder|ear infection|ear pain|ectropion|edema|elevated blood pressure|elevated liver function tests|emotional disturbances|emotional lability|Endocrine Disorders|endometrial disorder|endometriosis|enlarged uterus|enlargement|epilepsy|eruptions|erythema|erythema multiforme|erythema nodosum|exacerbation of asthma|Eye Disorders|eye infection|eyelid edema|facial rash|Family Stress|fatigue|female breast pain|Female Genital Disorders|Fetal Disorders|fever|fibrocystic breast|flatulence|flu|fluid retention|fungal infection|galactorrhea|gallbladder disease|gastritis|gastroenteritis|Gastrointestinal Disorders|gastrointestinal symptoms|generalized pruritus|Genital Disorders|genital edema|genital moniliasis|genital pruritus|glucose tolerance decreased|goiter|headache|hemangiomas|hematoma|hematuria|hemiparesis|hemorrhagic eruption|hemorrhoids|hepatitis|Hepatobiliary Disorders|hereditary angioedema|herpes|herpes infections|herpes simplex|hirsutism|hypercalcemia|hyperemia|hyperesthesia|hyperglycemia|hypernatremia|hypersensitivity|hypertonia|hypertriglyceridemia|hypesthesia|hypocalcemia|Immune System Disorders|incontinence|increased appetite|increased sweating|infection|Infestations|Influenza like illness|insomnia|Interactions|intestinal
127	edrophonium	-0.4392551	1.40692	3202	

128	brinzolamide	-0.43741799	1.39075	68844	foreign body sensation:eye itching:rhinitis:diplopia:blurred vision:kidney pain:hypersensitivity:keratopathy:hyperemia:eye pain:keratoconjunctivitis:dizziness:pruritus:discomfort:urticaria:blepharitis:dyspnea:dry mouth:pharyngitis:fatigue:dermatitis:lacrimation:dyspepsia:chest pain:diarrhea:crusting:Asthenopia:pain:dry eyes:nausea:hypertonia:alopecia:conjunctivitis:keratitis:headache
129	physostigmine	-0.52772408	1.38722	5983	bradycardia:convulsions:salivation:nausea:vomiting

130	testosterone	-0.51279401	1.38722	6013	nipple disorder:erythema:hepatic neoplasm:depressed mood:decrease in diastolic blood pressure:back pain:Eye Disorders:Nasopharyngitis:pain at the injection:Influenza like illness:breast tenderness:rash:acne:hemarthrosis:vein thrombosis:suicidal ideation:urinary tract infection:difficulty in micturition:aggressive:urinary incontinence:hypertension:abnormal dreams:urinary frequency:pruritus:gum irritation:testicular atrophy:Habituation:hostility:tenderness:abdominal pain:dry mouth:anemia:injection site inflammation:injection site pain:virilization:anaphylactoid reactions:hepatoma:polyps:allergic contact dermatitis:diabetes:application site edema:reflux:urinary urgency:nodules:urogenital disorder:priapism:urinary retention:enlarged prostate:pain:Pain in extremity:impatience:decreased libido:hyperplasia:nausea:syncope:induration:alopecia:elevated serum cholesterol:increase in systolic blood pressure:nephropathy:incontinence:gustatory sense diminished:hypokalemia:vasovagal reaction:elevated total bilirubin:deep vein thrombosis:hair discoloration:carcinoma of the prostate:leukopenia:leukoplakia:red blood cell count increased:SGOT increased:gingivitis:pneumonia:sinusitis:seborrhea:urination impaired:apnea:precocious puberty:necrosis:atrophy:chills:purpura:scarring:hypersensitivity:gamma glutamyl transpeptidase increased:varicocele:elevated blood pressure:edema:Oral irritation:skin disorder:dizziness:accelerated growth:detachment:Penile Pain:abnormal liver function:fatigue:prostatic specific antigen increase:nocturia:neoplasm prostate:Endocrine Disorders:papular rash:dry skin:sore gums:vertigo:dermatitis acneiform:asthma:abnormal renal function:skin nodule:dysuria:parosmia:confusion:gastrointestinal hemorrhage:hepatitis:infection:enlargement:oral lesions:respiratory disorder:hirsutism:ulcer:desquamation:cerebral hemorrhage:flushing:low HDL:elevated hemoglobin:abnormal hair growth:sensitization:buccal inflammation:Gastrointestinal Disorders:hyperlipidemia:hematocrit increased:Urinary System Disorders:fasting:elevated serum creatinine:anger:pattern baldness:contact dermatitis:coronary artery disease:LIPID ABNORMALITY:atopic dermatitis:jaundice:prostatitis:paresthesia:folliculitis:emotional lability:gum redness:benign prostatic hyperplasia:hematuria:Nervous System Disorders:prostate hypertrophy:stomatitis:prostate cancer:depression with suicidal:nasal edema:nervousness:inflammation:blood glucose increased:surgical intervention:nipple pain:thrombosis:vascular disease:libido increased:SGPT increased:hesitancy:Hepatobiliary Disorders:sickness:skin irritation:soft tissue
131	dihydroergocr	-0.43647756	1.38253	444034	
132	salsolinol	-0.43647756	1.38253	91588	

133	**azathioprine**	**-0.53392059**	**1.37675**	2265	vasculitis:withdrawn:rash:pneumonia:peliosis hepatis:marrow depression:panuveitis:myelosuppression:fever:pancreatitis:nodular hyperplasia:purpura:neutrophilic dermatosis:toxic nephropathy:leukopenia:hypersensitivity:thrombocytopenia:Negative nitrogen balance:hemorrhage:lymphoproliferative disorders:lymphoma:abdominal pain:agranulocytosis:anemia:abnormal liver function:aplastic anemia:skin disorder:hepatic insufficiency:death:Infestations:cancer:diarrhea:secondary infection:vomiting:steatorrhea:febrile neutrophilic dermatosis:pain:pancytopenia:distention:hyperplasia:nausea:myalgia:tumor:alopecia:arthralgia:interstitial pneumonitis:macrocytic anemia:infection:liver damage:malaise
134	tremorine	-0.43565742	1.37539	5534	abdominal distention|agitation|ataxia|blepharoconjunctivitis|blurred vision|coma|conjunctivitis|death|disorientation|distention|drowsiness|hallucinations|hyperemia|hyperkinesia|hyperpyrexia|incoherent|increased intraocular pressure|keratitis|paralysis|photophobia|punctate keratitis|rash|seizures|sweating|synechiae|tachycardia|urinary retention|vasodilation
135	5194442	-0.43531843	1.37245	5194442	
136	proscillaridin	-0.52436981	1.36653	4951	
137	homosalate	-0.43269399	1.34983	8362	

138	clebopride	-0.54078152	1.34679	2780	abdominal distention|abdominal pain|abdominal tenderness|abscess|acute renal failure|agranulocytosis|allergic rhinitis|alopecia|alveolitis|analgesia|anaphylactoid reactions|anemia|angina|angioedema|anxiety|aphthous stomatitis|aplastic anemia|arm pain|arthralgia|arthrosis|aseptic meningitis|asthenia|asthma|atopic|atopic dermatitis|atrial fibrillation|back pain|basal cell carcinoma|blisters|bloody stools|blurred vision|bradycardia|breast cancer|breast mass|bronchitis|bronchospasm|bursitis|cancer|carcinoma|caries|cellulitis|cerebrovascular accident|cerumen impaction|chest congestion|chest pain|chills|cholecystitis|chronic renal failure|colitis|confusion|congenital anomalies|congestive heart failure|conjunctivitis|constipation|contact dermatitis|cough|cramps|cyst|cystitis|death|deep vein thrombosis|dermatitis|diarrhea|disability|discomfort|dizziness|dry mouth|dry socket|dry throat|duodenal perforation|duodenal ulcer|dyspepsia|dyspnea|dysuria|edema|embolism|epidermal necrolysis|epigastric discomfort|epigastric pain|epilepsy|epilepsy aggravated|epistaxis|erythema|esophageal|esophageal ulcer|esophagitis|fatigue|fever|fibrillation|flatulence|flu|fluid retention|flushing|fungal infection|gas|gastric perforation|gastric ulcer|gastritis|gastroenteritis|gastrointestinal hemorrhage|gastrointestinal symptoms|hallucinations|heartburn|heart failure|hematoma|hemorrhage|hemorrhoids|hepatic failure|hepatitis|hernia|herpes|herpes simplex|hiatal hernia|hypercholesterolemia|hyperkalemia|hypersensitivity|hypersensitivity vasculitis|hypertension|hypertensive|hypertensive crisis|hypesthesia|hyponatremia|impaction|infarction|infection|infectious gastroenteritis|insomnia|interstitial nephritis|intestinal obstruction|irregular heart rate|jaundice|joint swelling|laryngitis|leukopenia|lower extremity edema|lymphoma|meningitis|menopausal symptoms|menopause|menstrual disorder|mouth ulceration|muscle weakness|musculoskeletal pain|myalgia|myocardial infarction|myopathy|nasal congestion|nausea|nephritis|neuropathy|nocturia|obstruction|oral infection|oral lesions|otitis|otitis media|palpitations|pancreatitis|pancytopenia|paresthesia|pelvic pain|peripheral edema|pharyngitis|photosensitivity|photosensitivity reaction|pneumonia|postoperative pain|pruritus|Psychiatric Disorders|pulmonary edema|pulmonary embolism|rash|reflux|renal failure|respiratory tract infection|rhinitis|sciatica|sinusitis|somnolence|spasm|stiffness|stomach|stomatitis|sweati
139	dydrogesteror	-0.43164421	1.34087	9051	abdominal cramps|cramps|diarrhea|nausea

140	diphenhydram	-0.53153779	1.33724	3100	rash:constipation:agitation:nausea:irritability:nasal congestion:blurred vision:chills:thrombocytopenia:nervousness:confusion:euphoria:wheezing:urinary frequency:dizziness:hemolytic anemia:agranulocytosis:dry mouth:anemia:fatigue:tachycardia:insomnia:sweating:paresthesia:vertigo:drug eruption:tinnitus:vomiting:hypotension:palpitations:urinary retention:convulsions:epigastric distress:chest tightness:diplopia:diarrhea:extrasystoles:anorexia:photosensitivity:tremor:labyrinthitis:sedation:increased sweating:drowsiness:shock:neuritis:headache
141	trimethobenza	-0.43100997	1.33548	5577	abdominal cramps|abdominal distention|abdominal pain|anaphylactoid reactions|anaphylaxis|angioedema|bloating|bullous eruption|colic|constipation|cramps|depressed level of consciousness|discomfort|distention|dizziness|drowsiness|dry mouth|dyspepsia|epidermal necrolysis|eruptions|erythema|erythema multiforme|fatigue|flatulence|Gastrointestinal Disorders|headache|hypersensitivity|ileus|Immune System Disorders|loss of consciousness|megacolon|nausea|Nervous System Disorders|pain|paralytic ileus|pruritus|rash|shock|toxic epidermal necrolysis|toxic megacolon|urinary retention|Urinary System Disorders|urticaria|vomiting
142	ioxaglic acid	-0.53251772	1.3279	3742	
143	acetazolamide	-0.52092233	1.3279	1986	jaundice:urticaria:numbness:paresthesia:toxic epidermal necrolysis:fatigue:hematuria:fever:pain at the injection:purpura:glycosuria:hypoglycemia:leukopenia:myopia:polyuria:tingling:confusion:excitement:nephrolithiasis:loss of appetite:growth suppression:ataxia:agranulocytosis:electrolyte imbalance:renal failure:anemia:abnormal liver function:injection site pain:hepatic insufficiency:acidosis:cholestatic jaundice:convulsions:flaccid paralysis:taste perversion:diarrhea:tinnitus:epidermal necrolysis:vomiting:dizziness:Hematologic Disorders:osteomalacia:pain:paralysis:hepatic necrosis:anaphylaxis:nausea:hyperkinesia:photosensitivity:hyponatremia:necrosis:aplastic anemia:hypokalemia:thrombocytopenic purpura:drowsiness:metabolic acidosis:melena:flushing:crystalluria:malaise:headache
144	cefoperazone	-0.53458377	1.31876	44185	hepatitis:pain:drug fever:pseudomembranous colitis:nausea:neutropenia:diarrhea:liver disease:vomiting:Local Reactions:fever:phlebitis:eosinophilia:colitis:abnormal liver function:hypersensitivity

145	chlorprothixer	-0.52808825	1.31876	667467	anemia|anorexia|anxiety|breast neoplasm|bullous eruption|cholestatic jaundice|confusion|cystitis|death|diarrhea|drowsiness|edema|encephalitis|encephalopathy|epidermal necrolysis|eruptions|erythema|gynecomastia|hematuria|hemolytic anemia|hemorrhage|hepatic encephalopathy|hepatic necrosis|hepatitis|hot flushes|hypertension|impotence|jaundice|laboratory test abnormal|leukopenia|loss of libido|macrocytic anemia|methemoglobinemia|nausea|necrosis|nervousness|photosensitivity|photosensitivity reaction|proctitis|rash|rectal disorder|rectal hemorrhage|SGOT increased|Sulfhemoglobinemia|thrombocytopenia|tumor|ulcer|urine discoloration|vomiting
146	esculin	-0.52174533	1.31876	5281417	
147	propranolol	-0.42888855	1.31757	62882	erythema:claudications:fever:respiratory distress:arterial thrombosis:aches:urticaria:tenderness:anaphylactoid reactions:paresthesia of hands:catatonic:bloating:pain:nausea:alopecia:Raynaud:arrest:flushing:injection site reaction:incoordination:conjunctivitis:purpura:sore throat:laryngospasm:hypersensitivity:skin ulcer:poor concentration:loss of libido:dizziness:BUN increased:pharyngitis:fatigue:weakness:lupus:vertigo:diarrhea:low cardiac output:AV block:tinnitus:disorientation:antibody positive:growth hormone deficiency:ischemic colitis:rash:constipation:paresthesia:bronchospasm:emotional lability:leukemia:lethargy:heart failure:congestive heart failure:ulcer:thrombosis:heart block:agranulocytosis:vivid dreams:arterial insufficiency:TEV:exfoliative dermatitis:dermatitis:nonthrombocytopenic purpura:mental depression:peripheral coldness:memory loss:colitis:anorexia:cramps:heart disease:thrombocytopenic purpura:drowsiness:abdominal cramps:mesenteric arterial thrombosis:erythema multiforme:amnesia:toxic epidermal necrolysis:precordial chest pain:lightheadedness:eruptions:systemic lupus erythematosus:sleep disturbances:lassitude:abdominal pain:dyspnea:abnormal vision:postural hypotension:insomnia:visual disturbances:hearing loss:epidermal necrolysis:vomiting:anxiety:rash erythematous:dry eyes:hallucinations:impotence:hypotension:epigastric distress

148	harmaline	-0.42848395	1.31417	6097179	abnormal liver function|acne|adenomas|alopecia|amenorrhea|anxiety|arthralgia|back pain|breast discharge|carpal tunnel syndrome|cataract|chills|cholestatic jaundice|constipation|convulsions|cramps|dizziness|edema|emotional lability|enlargement|eosinophilia|erythema|erythema multiforme|erythrocytosis|fatigue|fever|fluid retention|flushing|gastroenteritis|gum hemorrhage|headache|hematuria|hemorrhage|hepatic adenoma|hepatic neoplasm|hirsutism|hoarseness|hypertension|increased insulin|infarction|interstitial pneumonitis|intracranial hypertension|jaundice|joint swelling|laboratory test abnormal|leukocytosis|leukopenia|metrorrhagia|myocardial infarction|nasal congestion|nausea|nervousness|palpitations|pancreatitis|paresthesia|peliosis hepatis|pelvic pain|photosensitivity|pneumonia|polycythemia|pruritus|pseudotumor cerebri|purpura|rash|seborrhea|sleep disorder|sore throat|spasm|sweating|swelling|syncope|tachycardia|thrombocytopenia|tremor|tumor|urticaria|vaginal dryness|visual disturbances|vomiting|weakness|weight gain

S. Table 4: A list of primers used for RT PCR experiments.

Gene Name	Forward Primer	Reverse Primer
Ccl2(mouse)	GACCCGTAAATCTGAAGCTAA	CACACTGGTCACTCCTACAGAA
Cyclin D1 (mouse)	GCGTACCCTGACACCAATCTC	CTCCTCTTCGCACTTCTGCTC
Snai1 (mouse)	TGGTTCCTGCTTGGCTCTCT	TCTGGGCGGGTACAAAGG
Gapdh (mouse)	GCCATCAACGACCCCTTCAT	ATGATGACCCGTTTGGCTCC
CCL2 (human)	CAGCCAGATGCAATCAATGCC	TGGAATCCTGAACCCACTTCT
SNAI1 (human)	ACCACTATGCCGCGCTCTT	GGTCGTAGGGCTGCTGGAA
CDH1 (human)	GCTTGCGGAAGTCAGTTCAGA	CAAAATTCACTCTGCCCAGGAC
CCND1 (human)	ATGCCAACCTCCTCAACGAC	GGCTCTTTTTCACGGGCTCC
MYC (human)	AAGACTCCAGCGCCTTCTCTCCGT	TGGGCTGTGAGGAGGTTTGCTGTG
GAPDH (human)	AATTGAGCCCGCAGCCTCCC	CCAGGCGCCCAATACGACCA

S. Figure 1: Heatmaps of 85 IF/TA genes' expression across 6 different datasets.

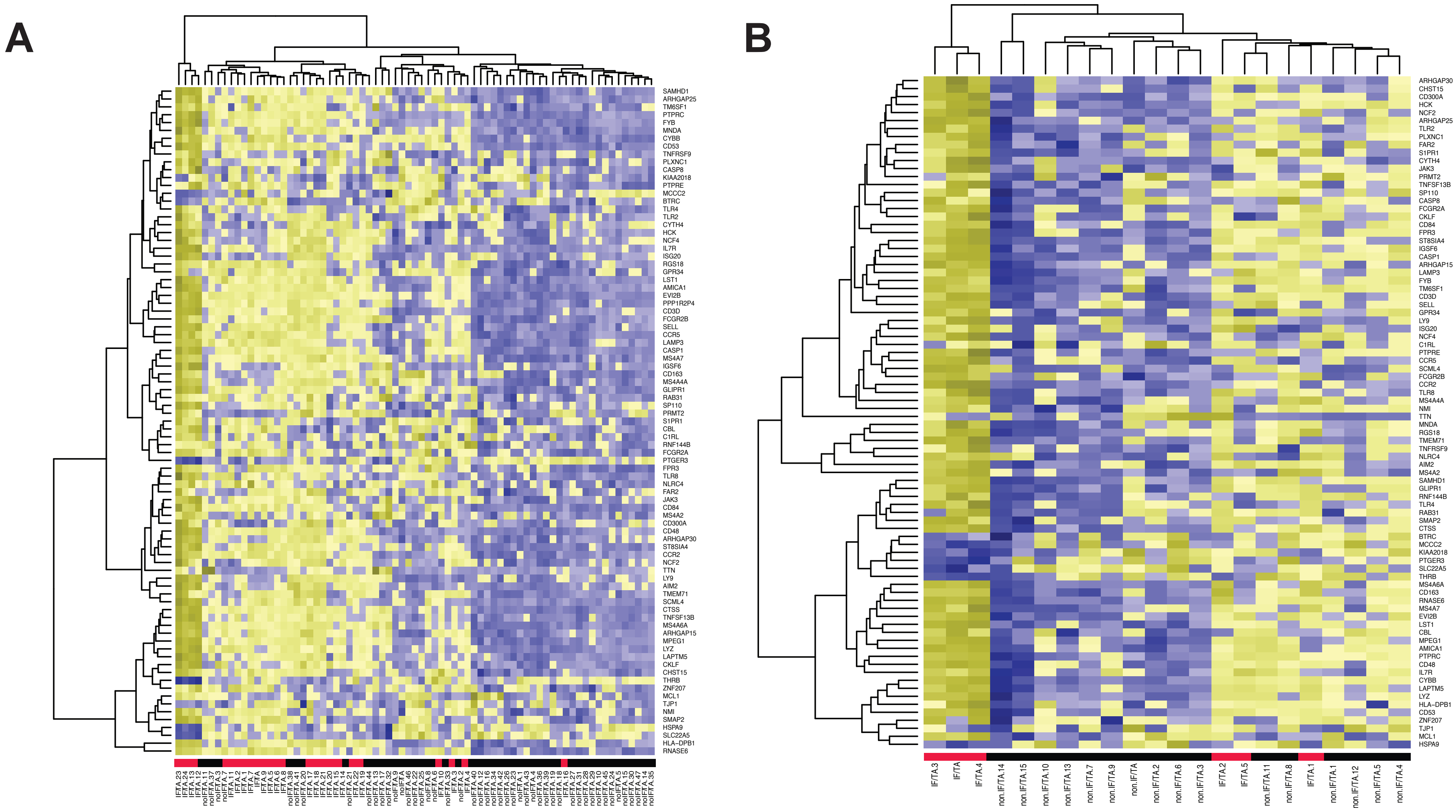

C

D

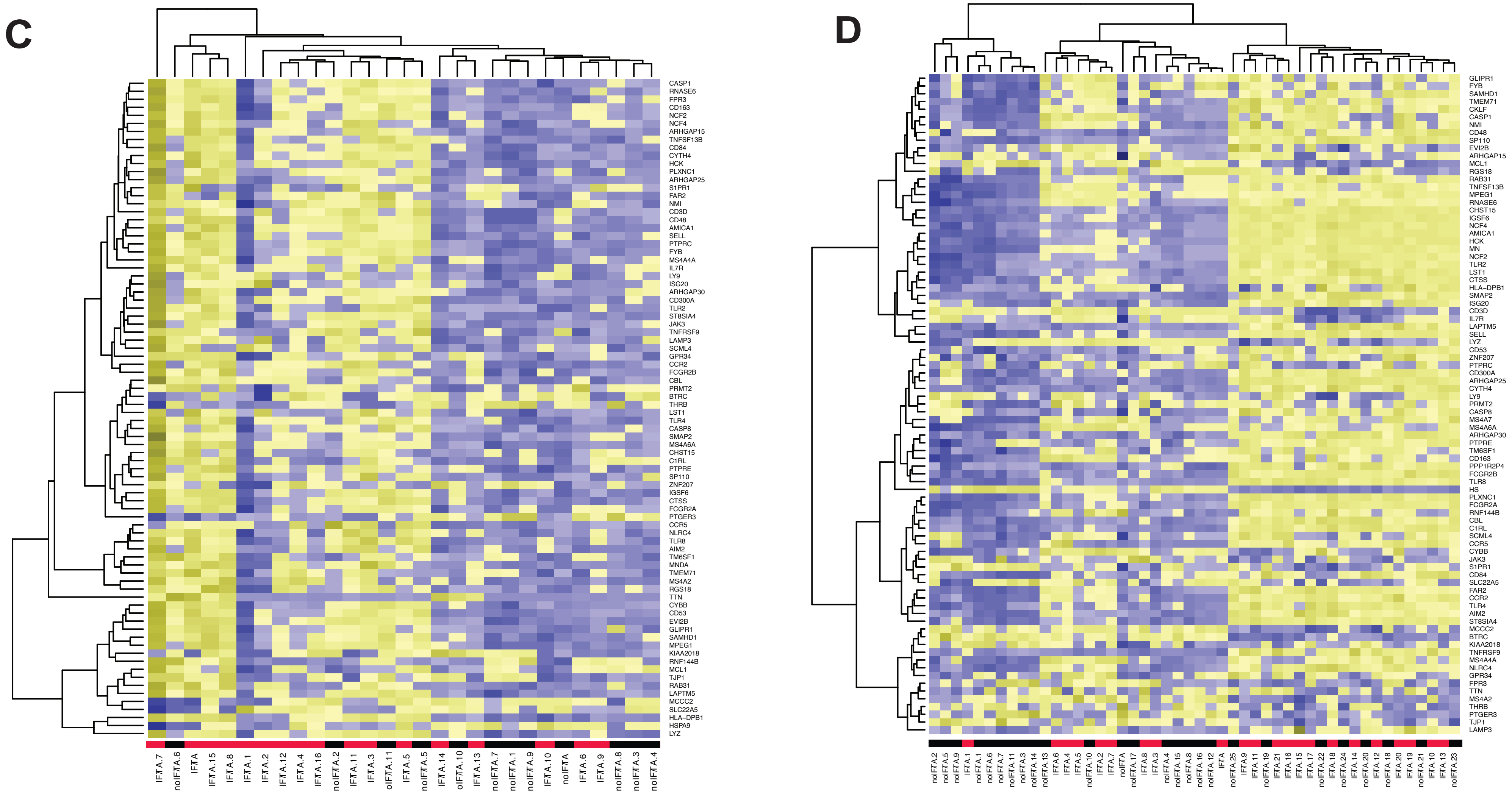
C
CASP1
RNASE6
FPR3
CD163
NCF2
NCF4
ARHGAP15
TNFSF13B
CD84
CYTH4
HCK
PLXNC1
ARHGAP25
S1PR1
FAR2
NMI
CD3D
CD48
AMICA1
SELL
PTPRC
FYB
MS4A4A
IL7R
LY9
ISG20
ARHGAP30
CD300A
TLR2
ST8SIA4
JAK3
TNFRSF9
LAMP3
SCML4
GPR34
CCR2
FCGR2B
CBL
PRMT2
BTRC
THRB
LST1
TLR4
CASP8
SMAP2
MS4A6A
CHST15
C1RL
PTPRE
SP110
ZNF207
IGSF6
CTSS
FCGR2A
PTGER3
CCR5
NLRC4
TLR8
AIM2
TM6SF1
MNDA
TMEM71
MS4A2
RGS18
TTN
CYBB
CD53
EVI2B
GLIPR1
SAMHD1
MPEG1
KIAA2018
RNF144B
MCL1
TJP1
RAB31
LAPTM5
MCCC2
SLC22A5
HLA–DPB1
HSPA9
LYZ
D
GLIPR1
FYB
SAMHD1
TMEM71
CKLF
CASP1
NMI
CD48
SP110
EVI2B
ARHGAP15
MCL1
RGS18
RAB31
TNFSF13B
MPEG1
RNASE6
CHST15
IGSF6
NCF4
AMICA1
HCK
MN
NCF2
TLR2
LST1
CTSS
HLA–DPB1
SMAP2
ISG20
CD3D
IL7R
LAPTM5
SELL
LYZ
CD53
ZNF207
PTPRC
CD300A
ARHGAP25
CYTH4
LY9
PRMT2
CASP8
MS4A7
MS4A6A
ARHGAP30
PTPRE
TM6SF1
CD163
PPP1R2P4
FCGR2B
TLR8
HS
PLXNC1
FCGR2A
RNF144B
CBL
C1RL
SCML4
CCR5
CYBB
JAK3
S1PR1
CD84
SLC22A5
FAR2
CCR2
TLR4
AIM2
ST8SIA4
MCCC2
BTRC
KIAA2018
TNFRSF9
MS4A4A
NLRC4
GPR34
FPR3
TTN
MS4A2
THRB
PTGER3
TJP1
LAMP3

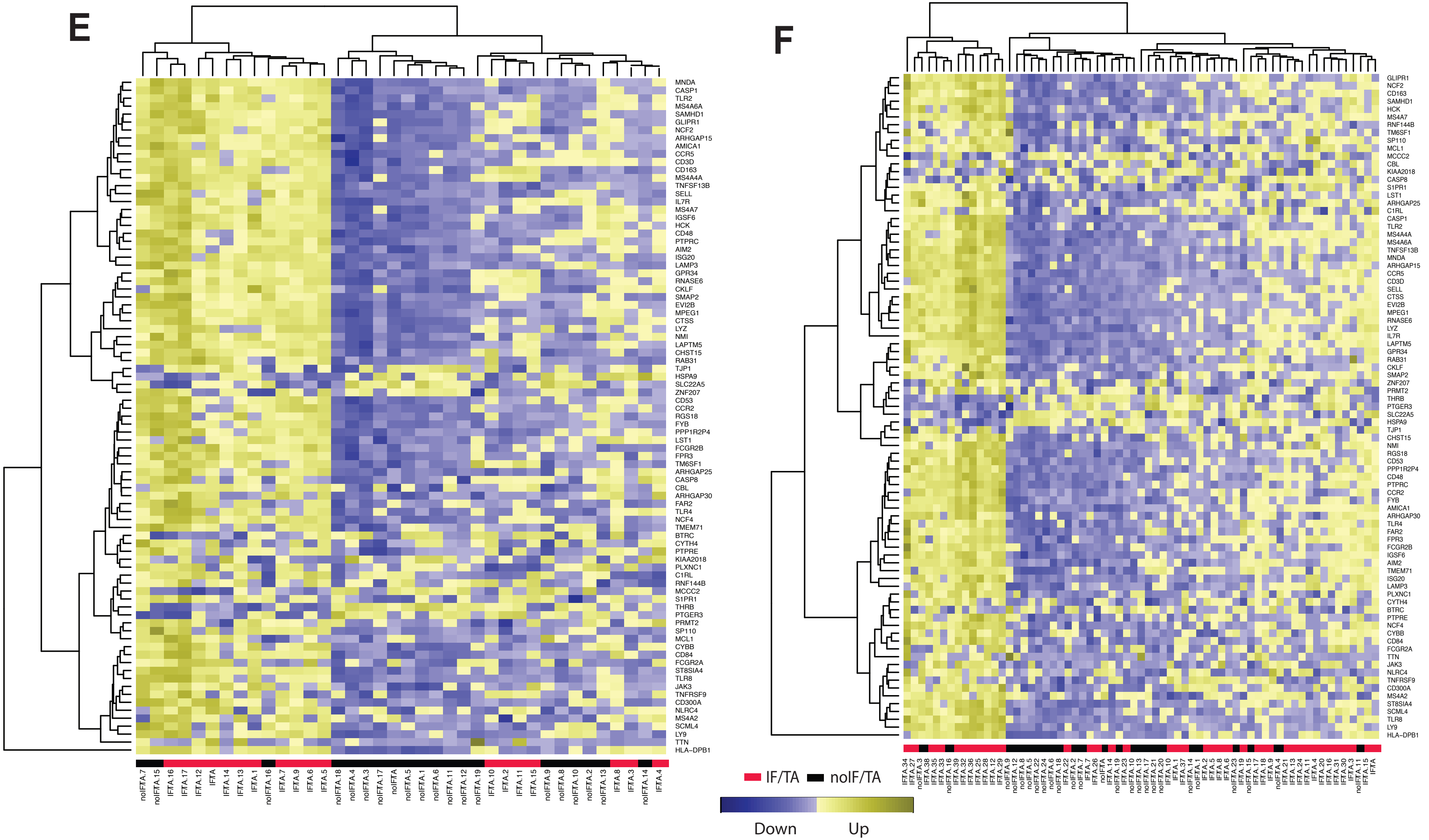

Heatmaps of 85 IF/TA genes' expressions were shown for all 6 independent datasets. (A). GSE25902 (Naesens 2010); (B). GSE74313 (In House 2016); (C). GSE44131 (Hayde 2013); (D). GSE12187 (Kurian 2009); (E). GSE9493 (Rodder 2009); (F). GSE22459 (Park 2010). Yellow color indicates up-regulation in IF/TA, and blue color indicates down-regulation in IF/TA samples. Red square indicates IF/TA samples, and black square indicates no IF/TA samples.

S. Figure 2: 1309 compounds ranking score driven by 85 IF/TA genes.

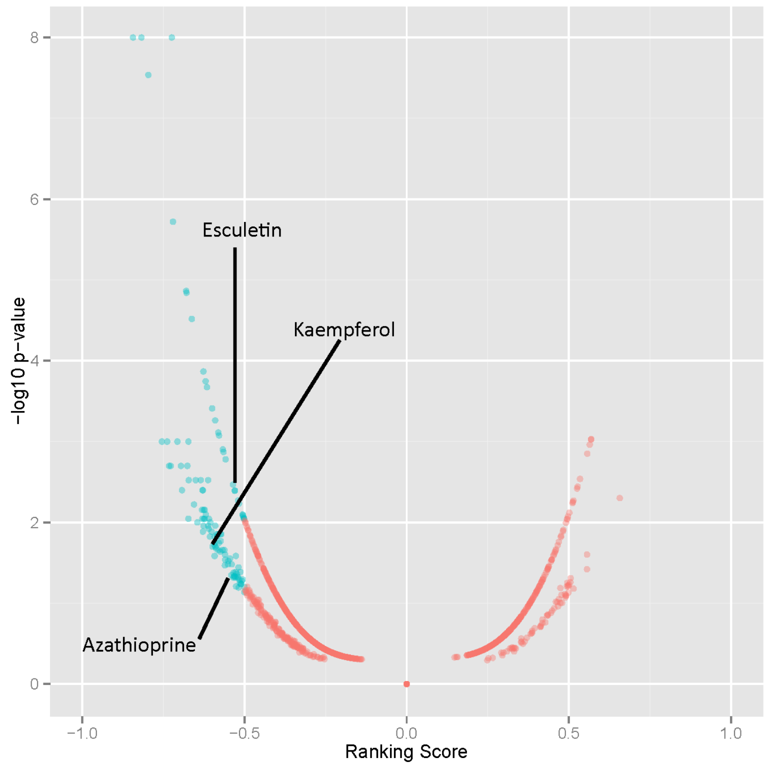

Compounds were shown in volcano plot (-log P-value against ranking score) with the significant top ranking (green, $P<0.05$) and non-significant ranking (red, $P\geq0.05$). We highlighted the one known drug to treat acute rejection after kidney transplantation and two proposed novel compounds.

S. Figure 3: Key genes out of 85 IF/TA genes drive the identification of kaempferol and esculetin.

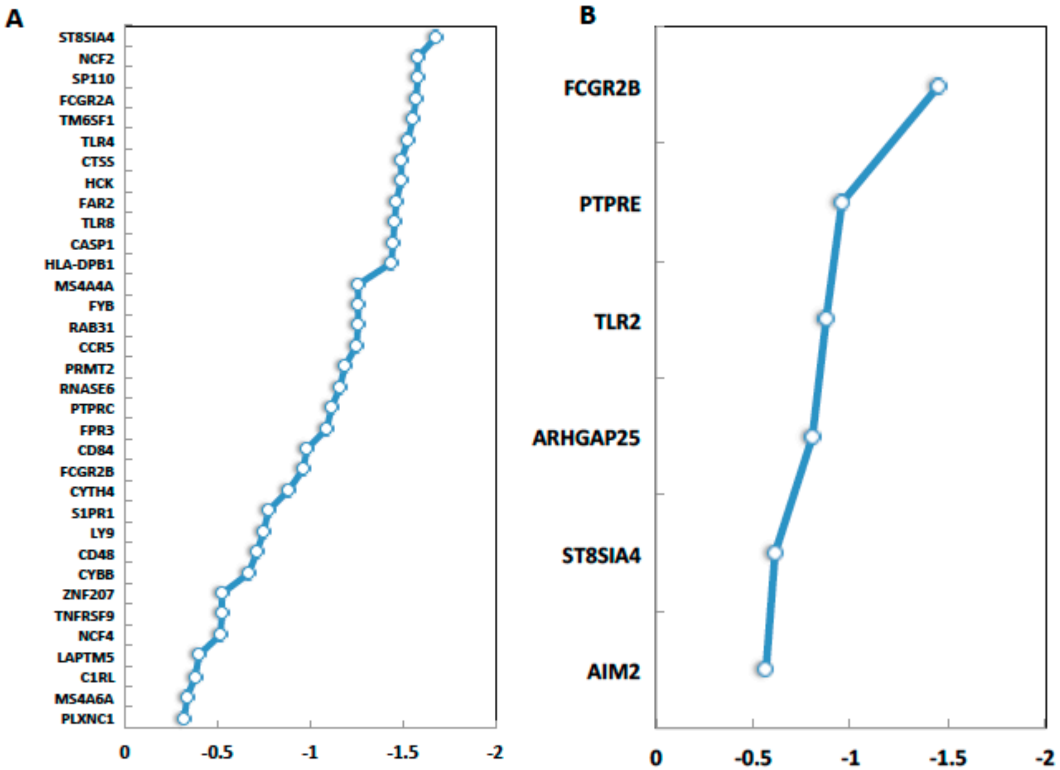

Key genes perturbed in kidney IF/TA, within the leading edge of the running sum enrichment score calculated for Kaempferol (A), and Esculetin (B). Gene expression shown as Z-score of ranked drug induced gene expression fold-change.

S. Figure 4: HK2 cell viability by Kaempferol and Esculetin.

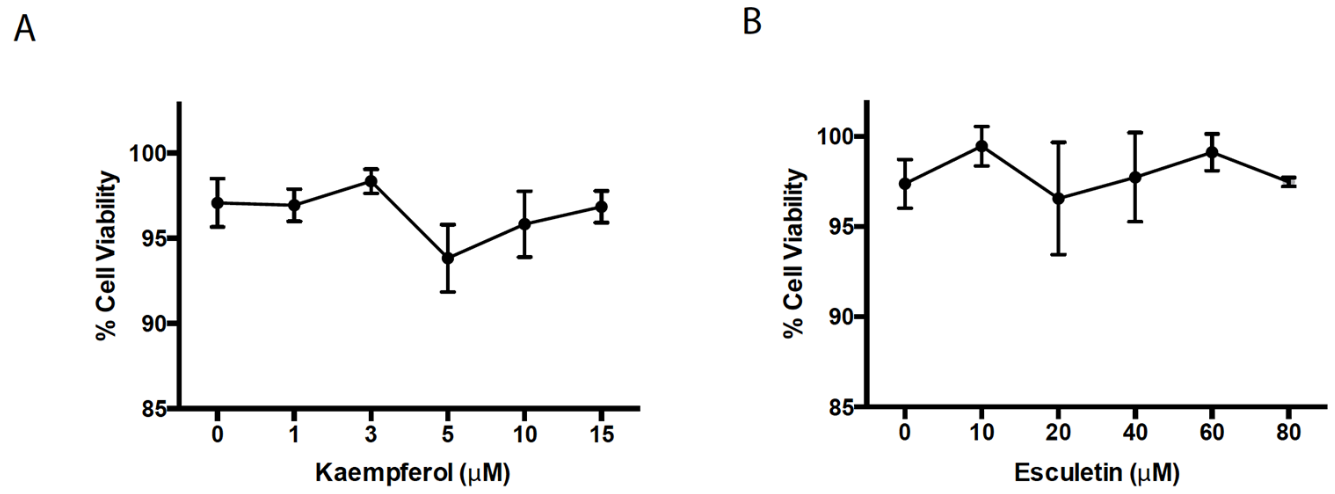

HK2 cells were treated with **(A)** kaempferol (1-15 μM) or **(B)** esculetin (10-80 μM) for 24 hours. Cell viability assayed using trypan blue exclusion method. Results were shown as duplicate cultures measured twice. No significant difference in cell viabilities for any concentration of kaempferol ≤ 15 μM or esculetin ≤ 80 μM. N=4 in each arm. Data was shown in mean and standard deviation.

S. Figure 5: Pro-fibrotic mediators on control samples by western blot.

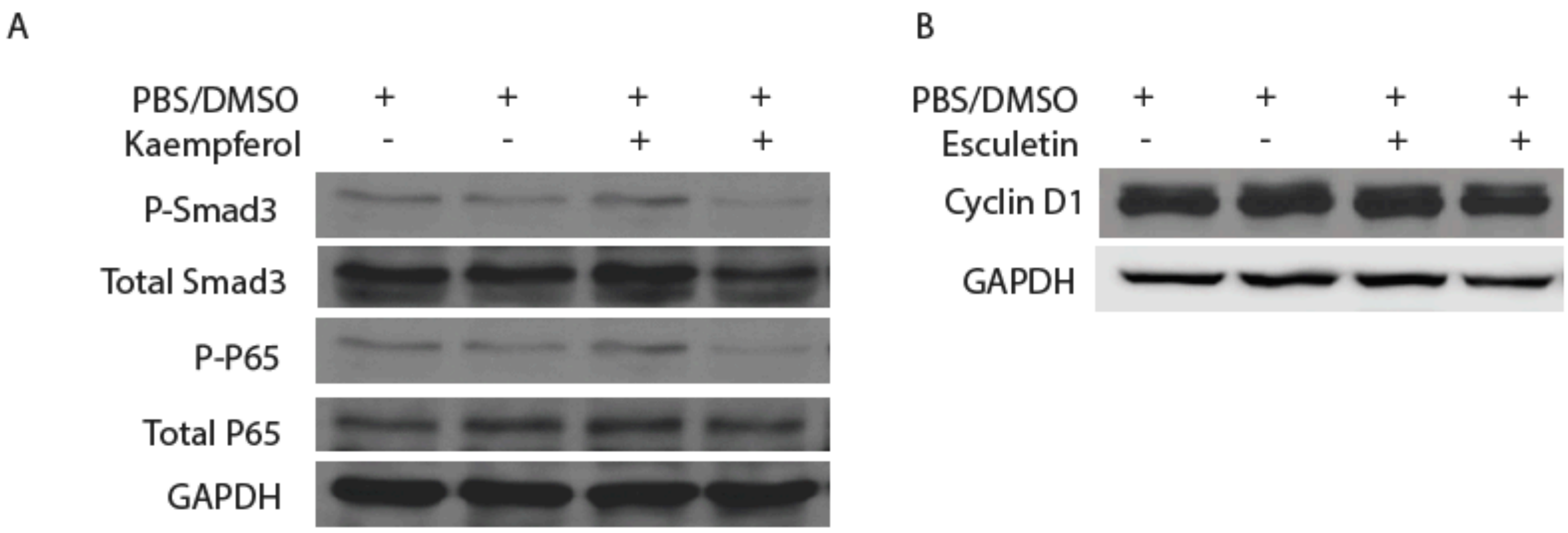

(A) Western blots in PSB/DMSO control mice for *P-Smad3*, total *Smad3, P-p65,* total *p65,* and *GAPDH* between kaempferol treated mice (N=2) and not treated mice (N=2). **(B)** Western blots in PSB/DMSO for *Cyclin d1* and *GAPDH* between esculetin treated mice (N=2) and not treated mice (N=2).

S. Figure 6: Venn diagram for overlapping drugs across 6 drug lists specific to each dataset.

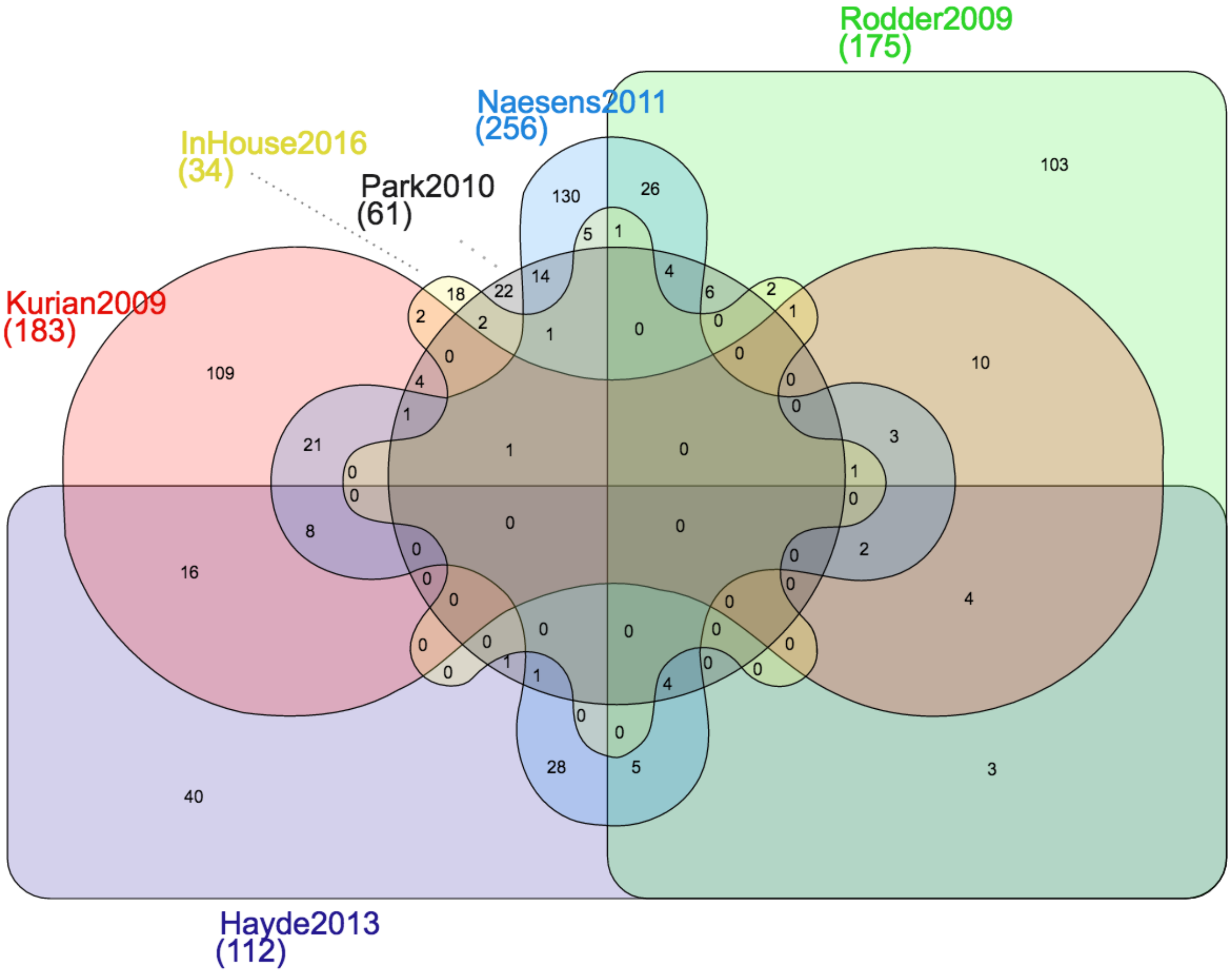

Venn diagram for overlapping drugs across 6 drug lists specific to each dataset. We identified 183, 256, 112, 175, 61, and 34 drugs with p<0.05 for Kurian2009, Naesens2011, Hayde2013, Rodder2009, Park2010, and In House2016 dataset respectively. Venn diagram was created by InteractiVenn[4].

REFERENCES:

1 Kuhn, M., Campillos, M., Letunic, I., Jensen, L. J. & Bork, P. A side effect resource to capture phenotypic effects of drugs. *Mol Syst Biol* **6**, 343, doi:10.1038/msb.2009.98 (2010).

2 Kuhn, M., Letunic, I., Jensen, L. J. & Bork, P. The SIDER database of drugs and side effects. *Nucleic Acids Res* **44**, D1075-1079, doi:10.1093/nar/gkv1075 (2016).

3 Tatonetti, N. P., Ye, P. P., Daneshjou, R. & Altman, R. B. Data-driven prediction of drug effects and interactions. *Science translational medicine* **4**, 125ra131, doi:10.1126/scitranslmed.3003377 (2012).

4 Heberle, H., Meirelles, G. V., da Silva, F. R., Telles, G. P. & Minghim, R. InteractiVenn: a web-based tool for the analysis of sets through Venn diagrams. *BMC bioinformatics* **16**, 169, doi:10.1186/s12859-015-0611-3 (2015).
